# The wheat *Seven in absentia* gene is associated with increases in biomass and yield in hot climates

**DOI:** 10.1093/jxb/erab044

**Published:** 2021-02-05

**Authors:** Pauline Thomelin, Julien Bonneau, Chris Brien, Radoslaw Suchecki, Ute Baumann, Priyanka Kalambettu, Peter Langridge, Penny Tricker, Delphine Fleury

**Affiliations:** 1 School of Agriculture, Food and Wine, The University of Adelaide, PMB1, Glen Osmond, SA, 5064, Australia; 5 John Innes Centre, UK

**Keywords:** Cereal, drought, E3 ligase, grain, heat stress, positional cloning, *Triticum aestivum*

## Abstract

Wheat (*Triticum aestivum* L.) productivity is severely reduced by high temperatures. Breeding of heat-tolerant cultivars can be achieved by identifying genes controlling physiological and agronomical traits when high temperatures occur and using these to select superior genotypes, but no gene underlying genetic variation for heat tolerance has previously been described. We advanced the positional cloning of *qYDH.3BL*, a quantitative trait locus (QTL) on bread wheat chromosome 3B associated with increased yield in hot and dry climates. The delimited genomic region contained 12 putative genes and a sequence variant in the promoter region of one gene, *Seven in absentia*, *TaSINA*. This was associated with the QTL’s effects on early vigour, root growth, plant biomass, and yield components in two distinct wheat populations grown under various growth conditions. Near isogenic lines carrying the positive allele at *qYDH.3BL* underexpressed *TaSINA* and had increased vigour and water use efficiency early in development, as well as increased biomass, grain number, and grain weight following heat stress. A survey of worldwide distribution indicated that the positive allele became widespread from the 1950s through the CIMMYT wheat breeding programme but, to date, has been selected only in breeding programmes in Mexico and Australia.

## Teaser

We narrowed down a genetic locus associated with improved physiology and yield components in wheat under heat stress to a gene showing sequence and expression variation.

## Introduction

As one of the world’s major crops providing 20% of human food, bread wheat (*Triticum aestivum* L.) has a vital role in food security (Shiferaw *et al.*, 2013). It is also the most widely produced crop, grown worldwide in a variety of climates, including harsh environments. Wheat productivity is affected by growing season drought and heat stresses, often in combination, which can cause almost complete yield loss in some cases (Fischer and Maurer, 1978; Prasad *et al.*, 2011). Due to the increasing occurrence of drought and heat stress, particularly in the Mediterranean region, the USA, India, and Australia, which are amongst the largest producers of bread wheat in the world, improving yield under these stresses is a priority (Asseng *et al.*, 2011; Lobell *et al.*, 2012). Targeting crop productivity in regions affected by drought and heat is believed to be the best strategy to reach the 1.6% yield improvement per year required to meet the food needs of an increasing world population (Lucas, 2013).

One way to deliver new, high-yielding varieties is to discover and deploy genes associated with grain yield variation in stress-prone environments through breeding. Many genetic studies have focused on the identification of quantitative trait loci (QTLs) associated with yield variation under abiotic stress, and, in wheat, several genes have been identified for tolerance of salinity, cold, aluminium, and boron toxicity (Sasaki *et al.*, 2004; Brini *et al.*, 2005; Pallotta *et al.*, 2014). Genetic variation in wheat yields in dry and hot conditions has been extensively studied using bi-parental populations (reviewed by Tricker *et al.*, 2018), but no genes underlying QTLs for heat and drought tolerance have yet been successfully identified. Grain yield is a complex trait, highly influenced by the environment and, although QTLs for yield have been identified, only a few have been used in breeding programmes due to inconsistent performance of the QTLs in different environmental conditions.

We focused on a QTL located on the long arm of chromosome 3B, *qYDH.3BL*, identified in a doubled-haploid (DH) population from the cross between the Australian wheats RAC875 and Kukri. These lines were selected for their contrasting physiological responses in Mediterranean-like climates: RAC875 is a water conservative line, whereas Kukri depletes water more quickly under stress (Izanloo *et al.*, 2008). In the RAC875×Kukri DH population, a multienvironment analysis of 21 field trials showed a strong genotype×environment interaction at the locus (Bennett *et al.*, 2012; Bonneau *et al.*, 2013). The QTL was expressed in the deep soil of northern Mexico, with the RAC875 allele positively contributing to yield, thousand grain weight, and early vigour. The Kukri allele at *qYDH.3BL* was associated with increased yield in southern Australian trials (characterized by shallow soils), but only when plants were irrigated (Bonneau *et al.*, 2013). Parent *et al.* (2017) subsequently observed that the allele effect at *qYDH.3BL* was dependent on growth temperatures. In this study, we fine-mapped the QTL to a short region of 690 kbp which includes 12 putative genes. Furthermore, we identified sequence variants and differential expression of a *Seven in absentia* (*SINA*) gene consistent with the positive effects of one allele on early growth in unstressed conditions and plant biomass following heat stress. We identified and tracked the allele in a worldwide collection of wheat accessions to enable its future use.

## Materials and methods

### Plant materials

A recombinant inbred line (RIL) population of 2000 individuals of the cross between RAC875 (RAC-655//SR21/4*Lance/3/4*Bayonet) and Kukri (Madden/6*RAC-177//Grajo/76-ECN-44) were screened for recombination breakpoints in the QTL *qYDH.3BL*. A set of 160 RILs was selected for the fine mapping of *qYDH.3BL*. Among these, 70 RAC875×Kukri RILs were chosen for their contrasting yield in the 2011 (Bonneau *et al.*, 2013) and 2012 field trials conducted in Mexico (Supplementary Table S1) and based on their recombination point in the QTL interval. Four near isogenic families derived from QTL-specific residual heterozygous lines were developed from RAC875×Kukri F2:6 RILs to further study the mechanism underlying *qYDH.3BL*. Four RILs with residual heterozygosity at the QTL (between markers AWG43_1 and AWG38) were selfed to obtain near-isogenic families where sibling lines have a homogenous background and only segregate at *qYDH.3BL*.

A second collection of 3000 RILs of the cross between Drysdale (Hartog*3/Quarrion) and Gladius (RAC875/Krichauff//Excalibur/Kukri/3/RAC875/Krichauff/4/RAC875//Excalibur/Kukri) was screened for recombinants in the QTL. A second set of 44 RILs was selected based on their recombination breakpoint in the QTL interval and their extreme yield values during the 2009 and 2010 Mexican and New South Wales field trials (Bonneau, 2012). The four parental lines RAC875, Kukri, Drysdale, and Gladius were included in each trial.

Nulli-tetrasomic lines cv. Chinese Spring (LV-Szechuan) of chromosome group 3 were used to test the specificity of chromosome-specific primers. The method used to obtain nulli-tetrasomic lines was previously described (Sears, 1966).

A diversity panel, combining 788 landraces and modern varieties of hexaploid wheat of which 743 accessions were of known origin, was used to identify the distribution of alleles at *qYDH.3BL* (Supplementary Table S2). A total of 544 accessions were spring wheat types originating from worldwide locations, with more than a quarter from Australia. We also used 244 worldwide accessions of winter wheat, mostly from Europe, from the INRA core collection (Balfourier *et al.*, 2007). Pedigree, year of release, and geographical origin of the lines were retrieved from the wheat pedigree portal, http://www.wheatpedigree.net .

### Genotyping and genetic map construction

DNA was extracted as described previously (Pallotta *et al.*, 2000). Gene-based markers were designed using the whole-genome sequencing (WGS) data of the parental lines (RAC875, Kukri, Drysdale, and Gladius) generated by BioPlatforms Australia using Illumina HiSeq sequencing technology (Edwards *et al.*, 2012). Sequence reads (100 bp) were aligned against the reference sequence International Wheat Genome Sequencing Consortium (IWGSC) RefSeq v1.0 of Chinese Spring (https://www.wheatgenome.org/) using DAWN (Watson-Haigh *et al.*, 2018). DAWN is a web interface that integrates multiple datasets including IWGSC RefSeq v1.0, RNA sequencing data of five tissues at three developmental stages (Bowne *et al.*, 2012), and WGS data of the parental lines. Single nucleotide polymorphisms (SNPs) within the *qYDH.3BL* interval were identified with a minimum of five reads per line at a SNP position. As regions with a high density of reads are more likely to be repetitive elements, SNPs with a coverage >50 reads were discarded.

Kompetitive allele-specific PCR (KASP) markers were designed with the Kraken software (LGC genomics, Middlesex, UK) on sequences of at least 100 bp spanning an SNP. Two allele-specific forward primers were designed for each SNP in combination with a common reverse primer. Each forward primer had a tail attached at the 5′ end specific to a fluorophore. The hybridization of one specific forward primer to the target sequence allowed the pairing of the fluorophore present in the KASP mix to the primer tail, releasing the quencher and allowing emission of fluorescence (Semagn *et al.*, 2014). KASP assays were performed using the KASPline (https://www.lgcgroup.com). KASP markers were prefixed AWG- and ADW- (Supplementary Table S3).

The genotyping data of the KASP markers were added to RAC875×Kukri and Drysdale×Gladius RIL genetic maps previously generated by Bonneau *et al.* (2013). Genetic maps were constructed using the ASMap package (Taylor and Butler, 2017) available in the software R. The genetic distance between each marker was calculated using the Kosambi mapping function (Kosambi, 1943) which is based on a new algorithm named MSTmap (Wu *et al.*, 2008). This algorithm can determine the marker order efficiently by establishing a minimum spanning tree. We also studied the allelic distribution of *TaSINA* using the closest markers, ADW594 and ADW595, in the wheat diversity panel.

### Deep soil phenotyping platform

Wheelie bins (100×57.5×51 cm) were filled with a mixed medium (one-third coco peat, one-third sand, and one-third clay). Twenty-five plants were grown per bin, in a 5 rows×5 columns grid with 10 cm space between each plant. Trials were conducted at the Waite Campus, Urrbrae, Australia, in a tunnel with a polyurethane cover. Meteorological data, air temperature, and relative humidity were recorded with a data logger (Kongin KG100, China) (Supplementary Fig. S1). Gypsum blocks (MEA, Magill, SA, Australia) were placed in the bins at two depths (10 cm and 40 cm) to record soil water potential (Supplementary Fig. S2).

In 2014 and 2015, RILs and parental lines were grown in a resolved latinized incomplete block using CycDesigN (Whitaker *et al.*, 2002), plus one filler plant placed in the centre of the bin. The design took into account the position of the lines according to a west/east axis and split the bins between inner and outer layers. Two trials were conducted in both 2014 and 2015. In 2014, RAC875×Kukri RILs were sown later than the normal planting season (44 RILs sown on 11 August and 20 RILs sown on 8 September, instead of May) to phenotype plants under combined drought and heat treatment. Plants were well watered during the first month for both treatments; watering was stopped at the booting stage for stress treatments. In 2015, both trials were conducted under dry and hot conditions: 30 RAC875×Kukri RILs were sown on 10 July, and 44 Drysdale×Gladius RILs were sown on 18 August.

In 2017, the near isogenic lines (NILs) were grown in a single plant plot design with 15 replicates each for the parental lines RAC875 and Kukri and 54 replicates of each pair of NILs. The design was developed using the function ‘prDiGGer’ in the DiGGer package in R for partially replicated designs (Supplementary Fig. S3). Replicates were distributed within the bins so that the same genotype was not present twice in the same row or column. In 2017, the plants were sown on 31 July; watering was maintained until late booting at the beginning of October and then stopped.

### Phenotypic evaluation in the deep soil-mimic system

Plant developmental stages were scored using the Zadoks’ scale around anthesis. Early vigour was scored using two methods. In 2014, during the first month after planting, photographs were taken for each bin every week, and early vigour was scored using a 1 (less vigorous) to 5 (more vigorous) comparative scale. In 2015, early vigour was monitored by measuring the total leaf area when a plant reached the four-leaf stage. The lengths and widths of each leaf on the main tiller were measured to determine the individual leaf area. The total leaf area was calculated as the sum of the leaf area (leaf width×leaf length×0.8) of the second and third leaves (Richards and Lukacs, 2002). Chlorophyll content of the flag leaf was estimated with a SPAD-502L meter (Ozaka, Japan) at three stages: booting, anthesis, and grain filling. Three measurements per flag leaf at each stage were taken to estimate the average value. Flag leaf area was measured from booting to flowering time using the LI-3000C portable leaf area meter (LI-COR Inc., Lincoln, NE, USA) in 2014. In 2015, flag leaf length and width were measured at the flowering stage with a ruler. Stomatal density was measured by taking imprints of the flag leaf adaxial face at anthesis using translucent nail polish on a glass slide (Shahinnia *et al.*, 2016). The slides were then analysed by microscopy using the Leica AS-LMD Laser Microdissection Microscope (Wetzlar, Germany). Three pictures per sample were taken and the guard cells number was counted for each.

Tiller number was recorded at the four-leaf stage and before harvesting. Tiller abortion was calculated at the end of the trial by subtracting the spike number from the tiller number. After harvest, spikes were manually counted to evaluate the number of spikes per plant. Spike and stem biomass were weighed separately, and harvest index was determined by dividing grain biomass by total above-ground biomass. Spike length was measured using a ruler before counting the number of spikelets per spike for each spike. Seed number was measured using a seed counter (Pfueffer GmBH, Germany) and then weighed to determine single grain weight. In 2014, carbon and nitrogen isotope discrimination of mature grains was measured on five grains per plant. Grains were dried for 1 week at 60 °C and finely ground to 1–4 mg of powder stored in tin capsules, pressed (Sercon, Gateway, Crewe, UK). The samples were sent to the University of California, Davis Stable Isotope Facility, University of California, for analysis using a continuous flow isotope ratio mass spectrometer.

Root traits were also evaluated in the 2014 experiment in deep-soil bins by digging out the roots at 10 cm depth after harvesting. The numbers of both seminal and nodal roots were measured. Photographs of the root system were taken for each plant and analysed using the Image J software (Schneider *et al.*, 2012). The root growth angle—the angle formed by seminal roots and the soil surface—was measured.

### Measurement of plant water use with sap flow sensors

In 2017, SF-4/5 Micro Stem Sap Flow sensors (Edaphic Scientific, Port Macquarie, NSW, Australia) were used in the wheelie bins on the NILs to measure ascending sap flow through the main stem and calculate plant water use (Smith and Allen, 1996). The measurements were non-destructive and continuous. Each sensor contained a heater located between two temperature probes. Data were automatically collected every 15 min after initial warming of the stem by the heater for 5 min. The output voltage was proportional to the difference in temperatures between the two probes. Sensors were installed on plants from mid-October when watering was stopped until harvest and placed between the first and second node. Data were recorded from the beginning of grain filling. Sensors were isolated with aluminium foil to avoid temperature fluctuations. Sensors were calibrated by measuring stomatal conductance (*g*_s_, mmol m^–2^ s^–1^) of plants every 2 h from pre-dawn until after sunset with an AP4 leaf porometer (Delta-T devices, Cambridge, UK). Collected raw sap flow data were normalized by setting a constant zero as the average of readings for 5 h during each preceding night and then averaged for every hour to calculate the mean average of the sap flow data for each allele within a NIL pair. The data were then plotted with the daily mean temperature. We also calculated the total sap flow rate per day and compared it within each NIL pair between the lines carrying the RAC875 allele and those with the Kukri allele.

### Early vigour imaging and water use analysis

Uniformly sized seeds of the NIL3 and NIL4 allelic pairs (*n*=6) were sown in 150 mm pots (one seed per pot) in coco peat compost and grown in a climate-controlled glasshouse containing an automated gravimetric watering and imaging platform, previously described (Brien, 2015), with day/night temperatures of 22/15 °C. The plants were arranged in two lanes×18 positions using a randomized complete-block design. After 25 d, images of each plant from each NIL and allelic pair were captured daily for the succeeding 25 d using the LemnaTec-Scanalyser 3D platform. Projected shoot area (PSA; kpixel) was estimated as the sum of the areas from three RGB camera views comprising two side views at 80° angles and one view from above. Water use was computed using the smoothed values of watering amounts (ml), and the water use index for a given interval was the ratio in change between PSA and total water use in that interval, with higher values corresponding to higher water use efficiency.

### Root morphological and anatomical measurements

In 2017, NILs were grown in black plastic pots (9 cm diameter×18 cm high) filled with a 50:50 soil mix of coco peat and sand, in greenhouse conditions with a photoperiod of 12 h. Six replicates per line were grown until sampling. Plants were harvested at two time points, 21 and 42 days after sowing (DAS). At 21 DAS, total leaf area of all the leaves from the main tiller was measured. Shoots were collected at both 21 and 42 DAS, dried at 50 °C for a week, and weighed. Roots were gently rinsed to remove any excess soil and then placed in 80% ethanol before storing them at 4 °C until measurements. Roots were separated from the root–shoot junction and aligned on a tray filled with water to scan them. Roots were scanned using the EPSON EXPRESSION 10000 XL scanner (Epson, Suwa, Japan). Each scan was then analysed using the RootGraph software (Cai *et al.*, 2015; https://www.quantitative-plant.org/software/RootGraph) to measure morphological traits including total root volume, total root length, total root area, and root tip number.

After scanning, samples of seminal roots were collected at two positions: 5 cm away from the tip and 2 cm away from the root–shoot junction. Once collected, samples were stored overnight in an aqueous fixative solution: 0.1 M phosphate buffer pH 7.4, 4% formaldehyde, and 0.25% glutaraldehyde. Root samples were then embedded in 5% agarose gel for sectioning using a Leica VT1200 S fully automated vibrating blade microtome (Leica, Wetzlar, Germany). Cross-sections as fine as 40 μm were stained in 0.5% toluidine blue and mounted with 90% glycerol. Slides were analysed with the Nikon Ni-E optical microscope (Nikon, Tokyo, Japan) and pictures were taken with a DS-Ri1 colour cooled digital camera (Nikon, Tokyo, Japan). Three technical replicates for both the root–shoot junction and the tip at both 21 and 42 DAS were analysed per genotype. Root anatomy features including root diameter, stele diameter, central metaxylem vessels diameter and number, protoxylem diameter. and number and thickness of the cortex were measured using the Image J software (Schneider *et al.*, 2012). A *t*-test in the R statistical environment (R Core Team, 2014) was used to identify statistically significant difference between the lines containing the RAC875 and the lines containing the Kukri allele for each trait measured.

### QTL analysis

The multienvironment QTL analysis of the RAC875×Kukri RILs combined four field trials conducted in Mexico, Ciudad de Obregon, in 2011 (Bonneau *et al.*, 2013) and 2012 (Bonneau, 2012), and was performed as described previously (Bonneau *et al.*, 2013). For the single-marker analysis, we generated the best predicted unbiased estimates (BLUEs) for each trait using Equation 1 and the asremlPlus package (Brien, 2015). The normal distribution of the BLUEs was then evaluated using the Shapiro–Wilk test (Shapiro and Wilk, 1965). Single-marker analysis at each marker position was performed to test association with the studied traits by a one-way ANOVA in R. For the trait which did not follow a normal distribution, a Kruskal–Wallis test was used (Kruskal and Wallis, 1952).

$$\begin{array}{l} Var\left[Y\right]=\left\{\left[\left(Rep/Bin\right)\times \left(Zone/Side\right)\right]/Position\right\} \end{array}$$ (1)

### Anchoring of *qYDH.3BL* onto the wheat reference sequence

Flanking markers at the QTL were used to find sequence similarity with the reference sequence RefSeq v1.0 of Chinese Spring by BLASTN (e-value –10; # hit return=100). The 1.5 Mbp sequence delimited by AWG43_1 and AWG38 was extracted using Fetch-seq, an in-house sequence retriever that allows the extraction of subsequences from the IWGSC RefSeq v1.0 using coordinates. Gene annotations were retrieved using the coordinated interval in the *Triticum aestivum* browser available at Ensembl Plants (http://plants.ensembl.org) and updated to release 49 v1.1. Genomic sequences of the annotated genes were used to find similarities with *Brachypodium distachyon* v3.1 and the *Oryza sativa* v7 reference genomes to identify putative gene function by BLASTN (default settings) in phytozome (https://phytozome.jgi.doe.gov/).

### Targeted assembly of the region in RAC875

Initial, stringent alignments of WGS reads of RAC875 (Edwards *et al.*, 2012) indicated that an ~150 kbp region in Chinese Spring RefSeq 1.0 (from adw477 to adw594) may be either absent or highly divergent in RAC875. Further, more relaxed alignments of RAC875 paired-end and mate-pairs data to RefSeq v1.0 failed to produce any evidence for the region being deleted.

Reads and their mates which remained unaligned and those which aligned within the region of interests were *k*-merized, *k*=64 using KMC2 (Deorowicz *et al.*, 2015). We then assembled *k*-mers occurring three or more times in the input data into unitigs, namely contigs unambiguously supported by *k*-mers, using yakat kextend (Suchecki *et al.*, 2019, Preprint]. Unitigs were scaffolded with the complete mate pairs and paired-end datasets using SSPACE (Boetzer *et al.*, 2011). The resulting scaffolds were aligned to RefSeq v1.0 using BLASTn to exclude those derived from other regions of the genome and to identify a subset likely to be derived from the region of interest. The total length of 80 scaffolds putatively identified as coming from the region of interest adds up to ~200 kbp and the longest scaffold covers 36 kbp. Scaffolds with a minimum length of 10 kbp were then annotated using TriAnnot (Leroy *et al.*, 2012).

### Gene sequencing, promoter analysis, and haplotyping

*TaSINA* chromosome-specific primers were designed with Primer 3 available in Geneious (Biomatters, Auckland, New Zealand) (Supplementary Table S5). Nulli-tetrasomic lines (Sears, 1966) specific to the group 3 chromosome were used to test the specificity of the primers through amplification of the amplicon fragment. PCR samples were Sanger sequenced at the Australian Genome Research Facility Ltd (http://www.agrf.org.au/) (Supplementary Fig. S4). Oligo primer sequences were conserved in RAC875, Kukri, and Chinese Spring. Promoter sequences of the RAC875 and Kukri *TaSINA* were annotated using the Plant Cis-acting Regulatory DNA Elements (PLACE) database which includes sequence motifs reported as *cis*-acting regulatory DNA elements (Higo *et al.*, 1999). Protein domains, residues, and motifs of *TaSINA* were identified using Prosite (Sigrist *et al.*, 2002) and Pfam (http://beta.supfam.org/).

The *TaSINA* sequence from Kukri was used to identify *TaSINA* in other wheat genome sequence assemblies by BLASTn in GrainGenes (https://wheat.pw.usda.gov/blast/) using default setting to query the 10+ Genome pseudomolecules (Walkowiak *et al.*, 2020) of the varieties Arina (v3), Jagger (v1.0), Julius (v1.0), LongReach Lancer (v1.0), CDC Landmark (v1.0), Mace (v1.0), SY Mattis (v1.0), Norin 61 (v1.1), *Triticum spelta* PI190962 (v1.0), and CDC Stanley (v1.2). Genomic sequences from –4 kbp to the end of the coding sequence (CDS) were retrieved from JBrowsers (Supplementary Fig. S5) and aligned using MUSCLE v3.8 from EMBL-EBI. We also studied haplotypes at the large QTL interval between AWG43_1 and AWG38 on chromosome 3B from the Crop Haplotypes website which showed shared haplotypes based on 1 Mb bins between the wheat genome assemblies generated by the 10+ Wheat Genome Project (Brinton *et al*., 2020; Walkowiak *et al*, 2020).

### Gene expression analysis

The wheat drought experiment cDNA series of RAC875 and Kukri was previously described (Bowne *et al.*, 2012). Additionally, we used 2-week-old seedlings of the NIL pairs for gene expression analysis via quantitative reverse transcription–PCR (qRT–PCR) with three biological replicates for each genotype and three technical replicates per biological replicate. Seedlings were grown in an air-conditioned glasshouse in pot trays filled with a potting mix (coco peat). Total RNA for both root and shoot tissues was extracted using the Spectrum™ Plant Total RNA Kit (Sigma-Aldrich, Carlsbad, CA, USA) following the manufacturer’s recommended protocol. cDNAs were synthetized using the SuperScript IV First-Strand Synthesis System (Invitrogen, Sigma-Aldrich) as per the manufacturer’s instructions and used for qPCR. qPCR primers targeting 70–200 bp amplicon sequences were designed to target the chromosome-specific gene copy (Supplementary Table S3). Specificity was tested with nulli-tetrasomic lines. qPCR assays were performed using the Kapa SYBR Fast Universal 2X qPCR Master Mix (Geneworks, Thebarton, South Australia, Australia) on the QuantStudio 6 Flex (Applied Biosystems, Foster City, CA, USA) following these steps: 95 °C for 3 min; followed by 40 cycles at 95 °C for 20 s, 63 °C for 20 s, 72 °C for 15 s. The initial run was then followed by a melting curve at 95 °C for 15 s, 60 °C for 1 min increasing by 0.05 °C per cycle up to 95 °C. Three technical replicates per sample and per gene, and four reference genes, namely *TaActin*, *TaCyclophilin*, *TaGAPDH* (glyceraldehyde-3-phosphate dehydrogenase), and elongation factor (*TaEFα*), were included in the analysis for normalization of gene expression.

Gene expression profiles were also extracted from the wheat gene expression atlas (http://www.wheat-expression.com/) using the gene set RefSeq v1.1 (Ramirez-Gonzalez *et al.*, 2018).

## Results

### Marker development in the *qYDH.3BL* region using two populations

**Fig. 1.**
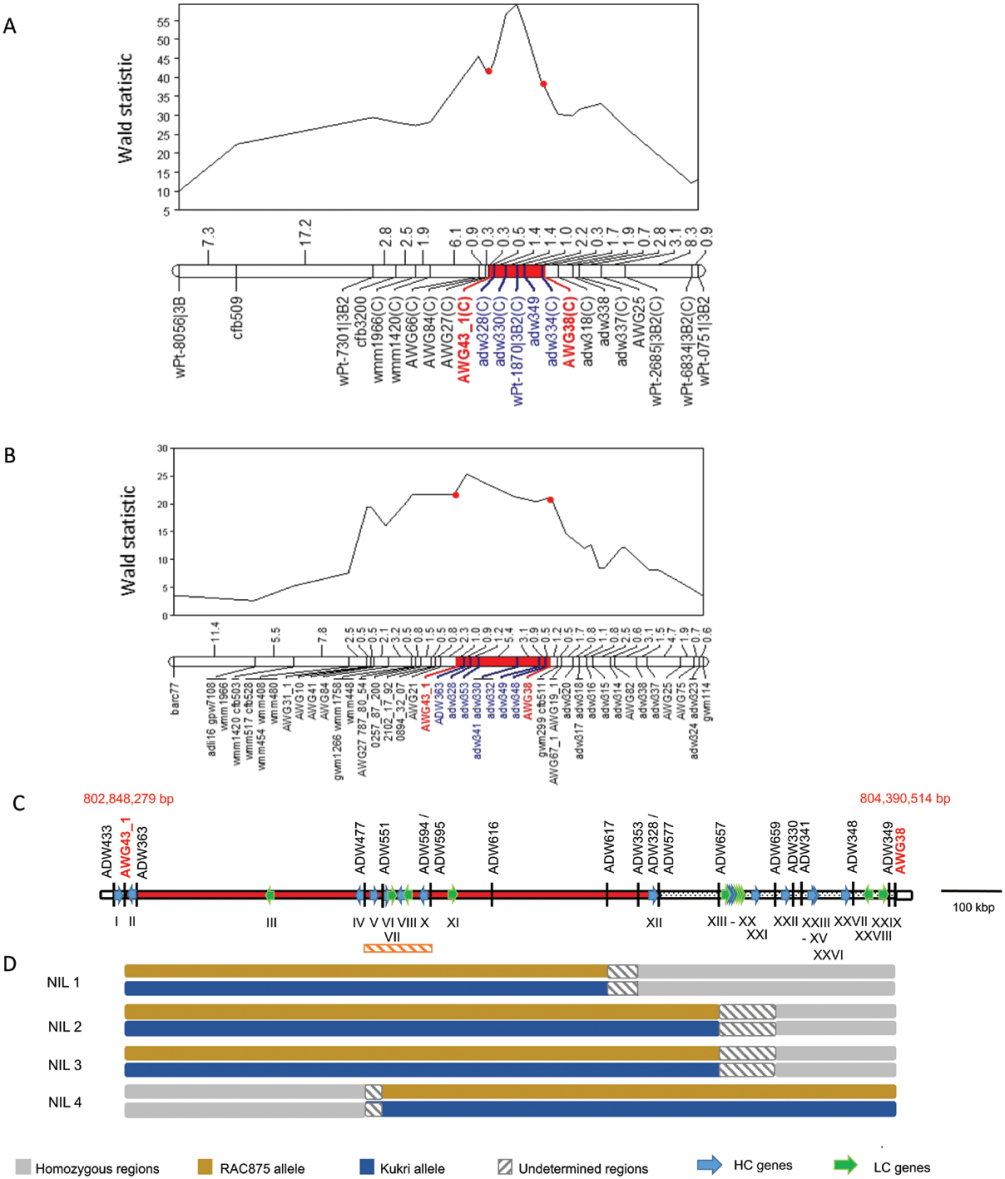
Genomic structure of *qYDH.3BL* in wheat. (A) QTL analysis of the Drysdale×Gladius recombinant inbred lines (RILs) in five field trials in Australia in 2009 and 2010. (B) QTL analysis of RAC875×Kukri RILs in four field trials conducted in 2011 and 2012 in Ciudad de Obregon, Mexico, delineating the QTL peak between the markers AWG43_1 and AWG38. (C) The physical interval (in red) of *qYDH.3BL* in RAC875 defined an interval of ~1 Mbp. HC, high confidence gene models; LC, low confidence gene models. The orange, hatched rectangle highlights the interval where RAC875 local assembly has been done. (D) Genotypes of RAC875×Kukri RILs with residual heterozygosity at *qYDH.3BL* used to generate near-isogenic lines (NILs).

The QTL *qYDH.3BL*, originally found in the spring wheat RAC875×Kukri DH population (Bennett *et al.*, 2012), was also present in a Drysdale×Gladius RIL population (Maphosa *et al.*, 2014). The analysis of these two populations across multiple trials in Australia and Mexico showed that at this locus the allele from RAC875 and Drysdale increased yield relative to the Kukri and Gladius allele (Fig. 1A, B). The multienvironment analysis of both populations showed that the high confidence QTL interval was flanked by the markers AWG43_1 and AWG38 (Fig. 1A, B). The RAC875 allele was positively associated with a 10% yield increase (Fig. 1B). We first fine-mapped *qYDH.3BL* in RAC875 by anchoring the interval defined by the multienvironment analysis onto the v1.0 reference sequence of cv. Chinese Spring (IWGSC RefSeq v1.0) (International Wheat Genome Sequencing Consortium, 2018). We then aligned the whole-genome shotgun sequencing data of the four parental lines (Edwards *et al.*, 2012) against the reference genome to identify sequence polymorphisms. Comparison between the genotypes RAC875–Drysdale versus Kukri–Gladius enabled us to identify new sequence variants. Seventy-four SNP-based markers (named AWG and ADW hereafter) and InDel markers were added to the previously described RAC875×Kukri RIL map (Bonneau *et al.*, 2013). The interval delimited by AWG43_1 and AWG38 defined an ~1.5 Mbp sequence in the new reference sequence assembly RefSeq v2.0, containing 29 gene models, 17 described as high confidence (HC) genes and 12 as low confidence (LC) genes (Fig. 1C). We found that this interval was smaller, only ~1 Mbp, in the RAC875 genome (Fig. 1C; Supplementary Fig. S6).

### Development of a deep soil-mimic platform for phenotyping

The QTL was highly significant for yield in Mexican field trials with heat treatment (by late planting with irrigation by flooding) (Bonneau *et al.*, 2013). These trials were conducted in the northern part of Mexico in the Sonora region where the climate is hot and dry: temperatures at flowering time averaged 25–28 °C for conventional sowing and 30–35 °C for late sowing; there is no rainfall during the growing season and water is provided by irrigation; and the soil profile is deep, with an effective soil depth of 110–120 cm (Verhulst *et al.*, 2009). The QTL was most significant in the Mexican trial sites, characterized by a deep soil profile, when flood irrigation and heat stress were applied to the crop, which suggested that the QTL gave an advantage in deep soil where water is stored at depth.

To identify the genes underlying the QTL, higher genetic map resolution was required, and new recombinants needed to be grown in conditions where the QTL has the strongest effects. As southern Australian soils are shallow, we used a deep soil-mimic platform to test the hypothesis that a deep soil profile, characteristic of the Mexican environments, triggered *qYDH.3BL* expression. Forty-four and 20 RAC875×Kukri RILs previously grown in Mexican field trials (Bonneau *et al.*, 2013) were planted in the deep soil platform in August and September 2014, respectively; late planting dates enabled us to phenotype the plants under combined drought and heat stress (Supplementary Figs S1, S2). The analysis of the September 2014 trial showed a significant positive effect of the RAC875 allele on most measured traits, increasing early vigour, stem biomass, plant height, spike length and biomass, grain and spike number per plant, spikelets per spike, chlorophyll content, and seminal root number, by at least 19% (Table 1). No effects were observed on stomatal density, or on carbon and nitrogen isotope discrimination in mature grains (data not shown). The single-marker analysis of the August 2014 experiment showed that the RAC875 allele was positively associated with a small increase in stem and spike biomasses, in tiller and spike number, and in number of spikelets per spike (Supplementary Fig. S7). Interestingly, the RAC875 allele was associated with a decrease in root growth angle, with 14.3% of the variance explained by ADW341. These results showed that the effects of *qYDH.3BL* in the deep soil-mimic platform were similar to those observed in Mexican field trials. We then used the platform for phenotyping recombinant RILs to narrow down the QTL size.

**Table 1.** Effects of the RAC875 allele at *qYDH.3BL* (using the AWG43_1 marker for single-marker analysis) in 20 RAC875× Kukri RILs and four lines (RAC875, Kukri, Drysdale, and Gladius) sown in September 2014 in the deep soil-mimic platform (*n*=3)

Phenotypic traits	Allelic effect^*a*^ (%)	*P*-value
Early vigour per plant	19.3	0.03
Plant height	20.8	0.03
Spike number per plant	20.8	0.03
Grain number	21.3	0.02
Spike biomass	21.3	0.02
Relative chlorophyll content in flag leaf	21.6	0.02
Spikelet per spike	25.1	0.01
Seminal roots	27.2	0.01
Stem biomass	27.6	0.01
Spike length	29.0	0.01

^*a*^ Allelic effect calculated as the percentage variance explained by the RAC875 compared with the Kukri allele.

The ANOVA comparing the two 2014 experiments showed highly significant differences in spike morphology, length, and number of spikelets per spike, but also spike biomass (Supplementary Table S4). The flag leaf area and tiller number were also significantly decreased in the September experiment compared with August. The average temperature around flowering time was min=15.4 °C and max=35.9 °C for the August 2014 trial and min=16.6 °C and max=33.4 °C for the September 2014 trial, so that temperatures at flowering were quite similar between the two experiments and did not seem to explain the differences between results (Supplementary Fig. S1). A quantitative analysis of soil water potential and air temperature effects on *qYDH.3BL* had previously found that the allele effect was correlated to temperatures (Parent *et al.*, 2017). The RAC875 allele increased individual seed weight, biomass, and harvest index when temperatures were above 25 °C around flowering time.

### Fine mapping of *qYDH.3BL* in RILs

**Fig. 2.**
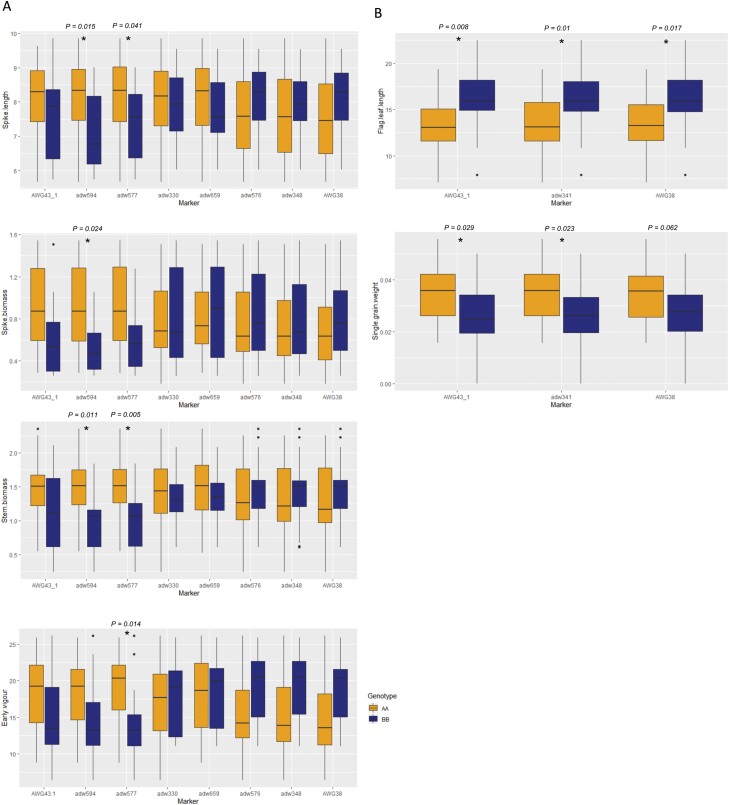
Single-marker analysis in 30 RAC875×Kukri RILs and 44 Drysdale×Gladius RILs segregating for *qYDH.3BL* and grown in dry and hot conditions in the deep-soil platform (2015). (A) Positive effects were associated with the RAC875 allele, while negative effects were associated with the Kukri allele. (B) Positive effects were associated with the Drysdale allele, while negative effects were associated with the Gladius allele. *P-value <0.05; **P-value <0.01

Out of 2000 RAC875×Kukri RILs, we found 30 lines with recombination breakpoints in the interval delimited by the AWG43_1 and AWG38 markers and grew them in the deep soil-mimic platform with heat stress to fine-map *qYDH.3BL*. In 2015, maximum temperatures at flowering time ranged between 34.8 °C and 38.8 °C (Supplementary Fig. S1). Spike length and biomass, stem biomass, and early vigour were significantly higher in plants carrying the RAC875 allele at the ADW594–ADW577 interval (Fig. 2A). Out of 3000 Drysdale×Gladius RILs, 44 RILs showed recombination within the QTL interval AWG 43_1–AWG38 and were phenotyped in dry and hot conditions, using the deep soil-mimic platform. The 44 RILs showed only two recombination points between AWG43_1, adw341, and AWG38. The single-marker analysis showed the positive allele from Drysdale associated with increases in flag leaf length and single grain weight that narrowed the interval in this population to markers AWG 43_1 and AWG38 (Fig. 2B), confirming the interval that was delineated based on field trials (Bonneau *et al.*, 2013).

The eight recombination breakpoints in the QTL region in the 30 RAC875×Kukri RILs enabled us to fine-map *qYDH.3BL* to an ~690 kbp sequence interval delimited by markers ADW594 and ADW577 which contained 12 annotated genes in the Chinese Spring reference sequence (Table 2). We also performed a local assembly of the RAC875 genomic sequence where no additional or missing annotated genes were detected in the interval compared with the Chinese Spring reference sequence (Supplementary Fig. S6).

**Table 2.** Genes annotated in the Chinese Spring v2.0 reference sequence 690 kbp interval of *qYDH.3BL*

	Gene ID	Position (bp)	Putative function based on rice orthologues	E-value	Putative function based on *Brachypodium* orthologues	E-value	Gene expression
**I**	TraesCS3B02G570900	802 845 950–802 848 503	LOC_Os10g33910.1_ mitochondrial import inner membrane translocase subunit Tim16	4.1 e-42	Bradi2g61480_mitochondrial import inner membrane translocase subunit TIM16	1.7 e-55	Supplementary Fig. S8
**II**	TraesCS3B02G571000	802 848 874–802 852 363	LOC_OS02g17280_Gamma-secretase subunit APH-1B	4.3 e-131	Bradi3g10110_endopeptidase activity	8.5 e- 135	Supplementary Fig. S8
**III**	TraesCS3B02G851200LC	803 363 524–803 364 407	–	–	–	–	Supplementary Fig. S9
**IV**	TraesCS3B02G572500	803 511 461–803 513 157	LOC_Os11g14410_ Polygalacturonase	8.5 e-142	Bradi2g57427_ Polygalacturonase/Pectinase	8.2 e- 87	Supplementary Fig. S8
**V**	TraesCS3B02G572600	803 523 701–803 525 213	LOC_Os01g72720_ expressed protein	8.9 e-11	Bradi2g61275_PF03478 Protein of unknown function	4.8 e-56	Supplementary Fig. S8
**VI**	TraesCS3B02G572700	803 540 158–803 540 583	–	–	Bradi1g36100_Glycosyl hydrolase, subfamily GH 28	1.6 e-22	Supplementary Fig. S8
**VII**	TraesCS3B02G851300LC	803 580 461–803 581 032	LOC_Os01g72620_ expressed protein	1.6 e-22	–	–	Supplementary Fig. S9
**VIII**	TraesCS3B02G572800	803 583 095–803 583 731	LOC_Os01g59540_GRF zinc finger family protein	1.1 e-4	Bradi5g21617	1.5 e-24	Supplementary Fig. S8
**IX**	TraesCS3B02G851400LC	803 586 310–803 587 263	LOC_Os07g42000_expressed protein	1.8 e-14	–	–	Supplementary Fig. S9
**X**	TraesCS3B02G572900	803 628 595– 803 630 088	LOC_Os01g03170_seven in absentia protein family protein	3.3 e-34	Bradi1g27970_ubiquitin-protein ligase activity	6 e-57	Supplementary Fig. S8
**XI**	TraesCS3B02G851500LC	803 715 648–803 716 915	–	–	–	–	Supplementary Fig. S9
**XII**	TraesCS3B02G573000HC	804 008 796–804 009 926	LOC_Os01g03170_seven in absentia protein family protein	2.7 e-31	Bradi1g27970_ubiquitin-protein ligase activity	1.7 e-48	Supplementary Fig. S8

The putative function of each gene was retrieved by homology with rice and *Brachypodium*. Gene expression information was retrieved from http://wheat-expression.com using RefSeq1.1. HC, high confidence; LC, low confidence.

### NIL development and phenotyping

**Fig. 3.**
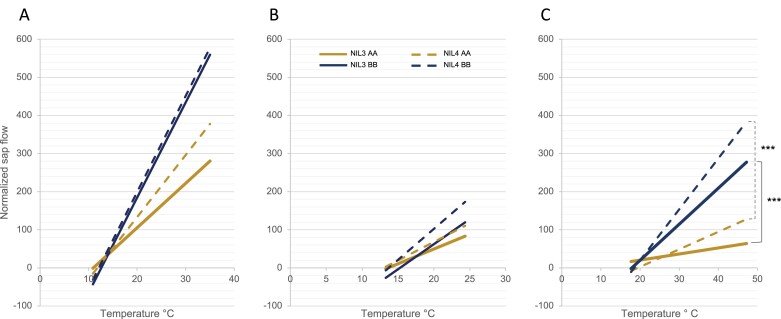
Regression of normalized sap flow of NIL3 and NIL4 families against temperature. (A) Temperate days, (B) cool days, and (C) hot days. AA, RAC875 allele (gold); BB, Kukri allele (blue). Differences between AA and BB alleles were non-significant on temperate and cool days. ***P<0.001 between AA and BB alleles on hot days.

As no further recombinants were found in our collection of 2000 RAC875×Kukri RILs, we developed NILs from heterozygous inbred families with recombination breakpoints spanning the QTL interval using RAC875×Kukri RILs with residual heterozygosity between AWG43_1 and AWG38 (Fig. 1D). Following crossing, these families segregate for the locus in contrasting pairs, but are otherwise near isogenic. In 2017, the lines were phenotyped under combined drought and heat treatment in the deep soil-mimic platform for yield components and main tiller sap flow, a proxy for plant transpiration (Smith and Allen, 1996). To avoid the confounding effects of phenology, we selected NILs 2, 3, and 4 with similar flowering time to the RILs previously studied. In the NIL2 family, the lines carrying the RAC875 allele had increased spike and stem biomass, increased grain weight per plant, and increased average number of spikelets per spike compared with those carrying the Kukri allele. In the NIL3 family, the lines carrying the RAC875 allele had increased single grain weight compared with those carrying the Kukri allele. In the NIL4 family, the lines carrying the RAC875 allele had increased spike and stem biomass, grain number, average number of spikelets per spike, and spike number compared with those carrying the Kukri allele (Table 3). Mass sap flow was, on average, significantly lower in all NILs carrying the RAC875 allele compared with those carrying the Kukri allele at the QTL over the period (Fig. 3). The regression of normalized sap flow mass against mean daily temperatures was significant (*P*<0.05) in all lines even though the proportion of the variance explained was low (adjusted *R*^2^=0.11–0.33). Whilst the difference was not significant early on during temperate days (average temperature 20.5 °C, Fig. 3A) or later in development (grain filling) during several cool days (average temperature 17.5 °C, Fig. 3B), there was a significant difference in sap flow between RAC875-allele NILs versus Kukri-allele NILs during heat stress (average temperature 31.5 °C) (Fig. 3C). At this time, sap flow in NILs carrying the RAC875 allele was always lower than in NILs carrying the Kukri allele as the RAC875-allele lines failed to increase sap flow in response to increasing temperature in comparison with the alternative allele.

**Table 3.** ANOVA of the NILs carrying the RAC875 (AA) or Kukri (BB) allele at *qYDH.3B* and grown in wheelie bins under drought and heat stress in 2017

Traits	Early vigour (cm^2^)	Spike biomass (g)	Stem biomass (g)	Grain number	No. of spikelets per spike	Single grain weight (mg)	Average spike length (cm)	Spike number	Tiller number	Plant height (cm)
NIL2-AA	25.52±3.59	1.95±0.74	2.74±0.72	49.63±16.30	18.04±0.48	19.93±3.81	9.23±0.30	1.78±0.83	3.67±0.87	52.22±3.85
NIL2-BB	23.75±4.05	1.21±0.34	1.81±0.43	37.75±7.70	17.48±0.47	16.65±3.2	8.87±0.51	1.38±0.52	3±0.53	48.39±4.91
df	16	16	16	15	16	16	16	16	16	16
*F*-test (*P*)	0.36	0.02*	0.006**	0.06	0.03*	0.08	0.09	0.26	0.08	0.09
NIL3-AA	19.40±2.81	1.77±0.46	3.01±0.78	40.63±10.2	16.25±0.94	29.17±5.23	8.89±2.98	1.58±0.51	3.62±0.51	54.68±5.11
NIL3-BB	17.66±2.63	1.46±0.97	2.58±1.26	31.08±17.6	16.07±1.17	22.5±5.65	8.64±2.94	1.64±0.84	3.5±1.02	50.9±6.01
df	26	25	25	23	25	24	25	25	26	25
*F*-test (*P*)	0.108	0.31	0.31	0.69	0.68	0.007**	0.35	0.83	0.72	0.1
NIL4-AA	24.81±2.71	2.38±0.91	3.05±0.9	67.36±13.74	16.3±0.39	17.10±2.80	8.03±0.41	2.75±0.62	3.58±0.79	54.05±4.56
NIL4-BB	22.49±2.76	1.69±0.54	2.26±0.55	51.45±14.44	15.74±0.81	18.56±2.92	7.88±0.44	1.91±0.7	3.27±0.79	51.73±5.62
df	22	22	22	21	22	22	21	22	22	22
*F*-test (*P*)	0.06	0.04*	0.02*	0.02*	0.05	0.33	0.41	0.006**	0.36	0.29

The values represent the mean ±SD. Early vigour was monitored by measuring the total leaf area (cm^2^) when a plant reached the four-leaf stage. *P-value <0.05; **P-value <0.01.

**Fig. 4.**
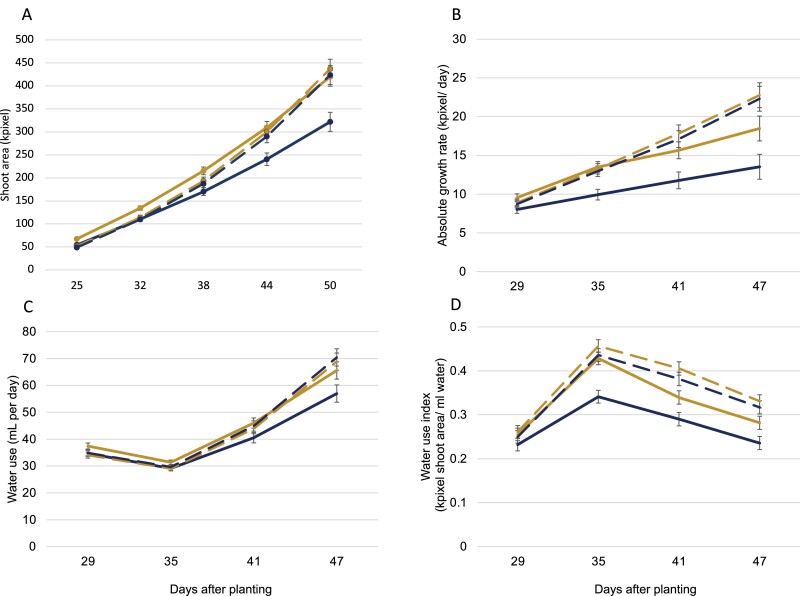
Early vigour and water use of NILs grown in unstressed conditions. (A) Estimated shoot area (kpixel) and (B) absolute growth rate (kpixel d^–1^) of young NIL3 (solid line) and NIL4 (hatched line) family plants. AA, RAC875 allele (gold); BB, Kukri allele (blue). (C) Water use (ml d^–1^) and (D) water use index (shoot area kpixel ml^–1^).

To verify the hypothesis that the RAC875 allele provides a yield advantage by increasing early vigour before the onset of drought or heat stress, we grew the NIL3 and NIL4 allele pair plants on an automated, gravimetric watering system with real-time imaging of early vegetative growth and measurement of water used per plant in unstressed conditions. Early vigour was increased in NIL3 lines carrying the RAC875 allele (Fig. 4), consistent with the allelic effect observed in the RIL experiments in deep soil (Table 1) and the increase in final biomass observed for this NIL (Table 3), but the effect was not significant in NIL4. In unstressed conditions, NIL4 with the RAC875 allele also had increased water use compared with the Kukri allele, in complete contrast to its reduced sap flow during heat stress. The water use index (kpixel ml^–1^ H_2_O) of NIL4 was increased in plants carrying the RAC875 compared with the Kukri allele, showing that this line’s early increase in biomass did not demand increased water use in unstressed conditions; that is, that the RAC875 allele at the locus conferred both increased early vigour and increased water use efficiency in unstressed conditions.

We measured root traits at 21 and 42 DAS in pots under controlled conditions. We did not observe any significant differences between parental lines or between NIL pairs for the root morphology traits including total root volume, total root length, total root surface, and number of root tips (Supplementary Table S5). Small differences were found in root anatomy between the two alleles in parental lines and NILs. At 21 DAS, we observed that RAC875 and NIL3 with the RAC875 allele had significantly (*P*<0.05) less protoxylem than lines with the Kukri allele (Supplementary Table S6). We also observed that RAC875 had more central metaxylem vessels than Kukri but the diameters of the metaxylem vessels were smaller in RAC875 compared with Kukri (Supplementary Table S6). Lines containing the Kukri allele had smaller central metaxylem vessels than those with the RAC875 allele, but only for NIL3 as no significant difference was found for the other NILs.

### Gene sequence analysis

We then analysed the sequence of the 12 gene models present in the QTL interval delineated by ADW594 and ADW577 markers using WGS datasets of RAC875, Kukri, Gladius, and Drysdale in DAWN. No non-synonymous variation was identified in the protein sequences encoded by the 12 genes at the locus when the RAC875–Drysdale versus Kukri–Gladius translated sequences were compared. Therefore, functional variation was most likely to lie at the gene expression level.

We next studied the expression profile of these gene models in the four NIL pairs that contrasted for yield components in deep soil. We first looked for gene expression in Wheat Gene Expression Atlas RefSeq v1.1 which includes 36 RNAseq datasets from different varieties (Ramirez-Gonzalez *et al.*, 2018) (Table 2). Among the eight HC genes, genes I, II, V, and X showed high levels of expression, while genes IV, VI, VIII, and XII showed a low level of expression (Supplementary Fig. S8). Among the four LC genes, genes III, VII, and IX showed very low, possibly insignificant, expression (Supplementary Fig. S9). In contrast, gene XI also annotated as a LC gene in RefSeq v2, showed significant expression in several studies (Supplementary Fig. S9).

**Fig. 5.**
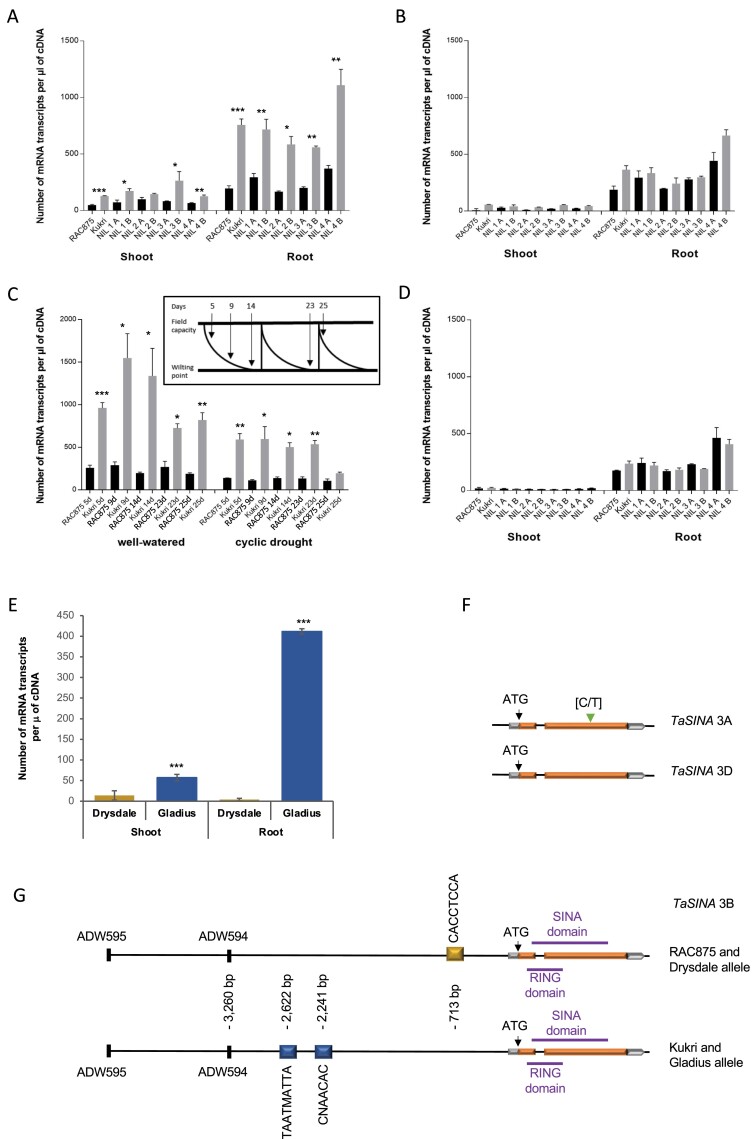
Expression analysis and gene structure of *TaSINA* and its homeologues. (A, E) Expression analysis of *TaSINA* in seedling shoot and root tissues of RAC875, Kukri, and NILs (A) and in Gladius and Drysdale (E). (C) Expression of *TaSINA* in RAC875 and Kukri under well-watered and cyclic drought conditions. (B, D) Expression analysis of *TaSINA* homeologues on chromosomes 3A (B) and 3D (D). ‘A’ corresponds to the RAC875 allele and ‘B’ corresponds to the Kukri allele. *P-value <0.05; **P-value <0.01; ***P-value <0.001. (F) Exon–intron structure of *TaSINA* homeologues on chromosomes 3A and 3D. (G) Exon–intron structure and promoter annotation of *TaSINA* on chromosome 3B. The CACCTCCA domain is a putative ABSCISIC ACID INSENSITIVE-4- (ABI4) binding site called S box (SBOXATRCS). Yellow boxes indicate the position of *cis*-acting elements present in the promoter region; green triangle, SNP position inducing the alanine to valine amino acid change; orange boxes represent the exons and the grey boxes the UTRs.

We measured the expression of the eight HC genes in the four NIL pairs, expecting a difference in expression between the RAC875 and Kukri alleles for the gene(s) responsible for *qYDH.3BL* effect. As the QTL was associated with early vigour when no treatment had been applied, we studied gene expression in seedlings grown in unstressed conditions. The expression profiles of genes I, II, IV, V, VI, VIII, and XII in RAC875 and the NILs carrying the RAC875 allele were not significantly different from Kukri and the lines carrying the Kukri allele (Supplementary Fig. S10). Gene X (*TaSINA*) was the only gene with a differential expression pattern both between the parental lines and among the NILs (Fig. 5A). The gene was consistently highly expressed and statistically different in the lines containing the Kukri–Gladius allele compared with the lines containing the RAC875–Drysdale allele, in both shoot and root tissues of seedlings, except for NIL2 in shoot tissue (Fig. 5A, E). In contrast, gene X homeologues on chromosomes 3A and 3D had the same expression profile in the two parental lines and the NILs independently of the allele that they contained and the tissue analysed (Fig. 5B, D).

The Wheat Gene Expression Atlas (Supplementary Fig. S8) showed that gene X was expressed in all plant tissues, with a higher expression in roots than in other tissues. We also analysed the expression profile of gene X in cDNA of RAC875 and Kukri grown in well-watered conditions and cyclic drought using the wheat RNA collection of the ACPFG (Bowne *et al.*, 2012). Gene X was significantly overexpressed in Kukri compared with RAC875 in both well-watered and drought treatments (Fig. 5C). Based on homology with rice and *Brachypodium*, gene X is homologous to a *SINA* gene encoding an E3 ubiquitin ligase protein, hereafter called *TaSINA*.

The translated Sanger sequencing of the *TaSINA* gene revealed two amino acid substitutions between the RAC875 (AP) and Kukri (VA) protein sequences at positions 303 and 304 (Supplementary Fig. S11) that were not previously found using the shotgun sequencing dataset and were not predicted to affect the protein function. We also found sequence polymorphisms in the promoter region, with the predicted *cis*-acting elements CANBNNAPA and HDZIP2ATATHB2 present in Kukri and absent in RAC875 (Fig. 5G). In RAC875 and Drysdale promoter sequences, the insertion of a CCAC domain 713 bp upstream of the start codon created a SBOXATRBCS (CACCTCCA) motif. The sequences of the *TaSINA* homeologues on chromosomes 3A and 3D were monomorphic between RAC875 and Kukri. The alignment of the TaSINA predicted protein sequence and its homeologues revealed an amino acid change in the protein sequence of the 3A copy (Fig. 5F). An SNP in the coding sequence of the 3A copy induced a semi-conservative amino acid replacement of an alanine by a valine at the position of amino acid 230 (Fig. 5E; Supplementary Fig. S11).

### Haplotypes and allelic distribution of *qYDH.3BL*

We aligned *TaSINA* sequences from 14 wheats using the dataset from the 10+ Genome project available in GrainGenes, and Sanger sequences of the four parental lines to identify other variants. RAC875, Drysdale, Norin 61, Jagger, SY Mattis, Julius, Arina, and Spelta share the same sequence with the amino acid substitution AP (Supplementary Fig. S5) previously observed between RAC875 and Kukri (Supplementary Fig. S11). We also found that the insertion of a CCAC domain at –713 bp created a SBOXATRBCS motif in these varieties, in contrast to Kukri, Gladius, Mace, CDC Landmark, and CDC Stanley (Supplementary Fig. S12). Lancer showed a number of rare variants and either shared RAC875 or Kukri sequence, and therefore represents a third haplotype of *TaSINA*.

**Fig. 6.**
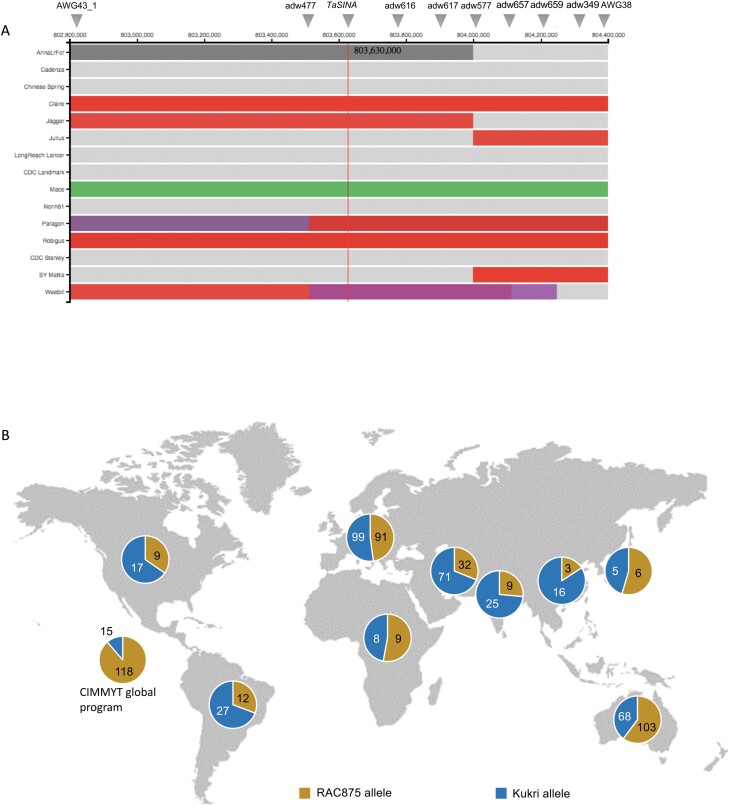
Haplotypes and allelic distribution at the *TaSINA* gene. (A) Haplotypes of the QTL large interval between AWG43_1 and AWG38 on chromosome 3B. Shared haplotypes are of the same colour in the same position (based on 1 Mb bins); sections in grey indicate a line-specific haplotype. The position of *TaSINA* is indicated with a red vertical line. (B) Allelic distribution in a diversity panel of 743 *T. aestivum* accessions genotyped with the closest SNP markers to TaSINA, ADW594 and ADW595.

We also looked at haplotype blocks covering the QTL interval to see the extension of historical recombination around *TaSINA*. We first looked at the interval covering the QTL interval between AWG43_1 and AWG38 using http://www.crop-haplotypes.com. We found three haplotypes at the *TaSINA* gene: red including the varieties Claire, Paragon, Rubigus, and Jagger which have the RAC875 allele at *TaSINA* (Supplementary Fig. S12), green for Mace which has the Kukri allele (Supplementary Fig. S12), and purple for Weebill (Fig. 6A). Interestingly Weebill showed a breakpoint at 803 510 000 bp, between genes III and IV (Table 2). DAWN also showed three haplotype blocks in this region using the NGS data of Australian cultivars (Supplementary Fig. S6): (i) Gladius and Kukri; (ii) RAC875, Drysdale, Westonia, and H45; and (iii) Wyalkatchem.

We investigated the worldwide allelic distribution of *TaSINA* alleles using the two closest SNP markers (ADW594 and ADW595) (Supplementary Table S2). The RAC875 allele was over-represented in accessions originating from CIMMYT in Mexico and in Australia compared with the Kukri allele which was more abundant in the germplasm originating from the Middle East, South America, and Asia (Fig. 6B). Both alleles were evenly represented in European and African wheats. The Kukri allele was present in historical wheat varieties such as Du Toits, Federation, Ward’s Prolific. and Hudson Early Purple Straw, released before 1900, and also in a large proportion of landraces (Supplementary Table S2). The RAC875 allele appeared later in the panel’s chronology, with most of the lines released after 1950, and seemed to follow the migration of germplasm released by CIMMYT during the green revolution of the 1960s (Supplementary Table S2).

## Discussion

The RAC875 allele at *qYDH.3BL* consistently increases early vigour, seminal root number, plant height, spike length, above-ground biomass, stem and spike biomass, spike number, number of spikelets per spike, and total grain number in deep soil following drought and heat stress. The association of *qYDH.3BL* with early vigour suggests that the mechanisms underlying the QTL are effective at an early stage of plant development, before abiotic stress is encountered. Early vigour is an important trait associated with water uptake, preventing water loss from soil evapotranspiration to the benefit of transpiration (Ludlow and Muchow, 1990). We hypothesize that this increased early growth in plants having the RAC875 allele had no significant effect under optimal conditions, as observed in southern Australian field trials in 2009 (Bonneau *et al.*, 2013), but was beneficial to the plant when temperatures increased.

As *qYDH.3BL* had previously been associated with differences in canopy temperature (Bennett *et al.*, 2012), a surrogate for plant evaporative cooling, we used sap flow sensors to evaluate the transpiration rates of whole plants (Smith and Allen, 1996). We observed that sap flow responded to daily temperature variations, but this response was abolished in lines carrying the RAC875 allele during heat stress late in development. These results are consistent with the observations of Izanloo *et al.* (2008) on the water management behaviour of RAC875 and Kukri parental lines under cyclic drought. They concluded that RAC875 reduced its water consumption at early stages of drought stress for later consumption when the stress become more severe. Here, the RAC875 allele increased plant biomass and water use efficiency at early, vegetative stages of development and reduced transpiration in response to heat late in development without any negative impact on yield components.

By fine-mapping the QTL and by expression analysis of eight genes in the QTL interval, we found that only the *TaSINA* gene showed consistent variation between lines carrying contrasting alleles at the QTL. Beyond those identified in the reference assembly, no other gene was found in the interval when we locally assembled RAC875 genomic sequence. As the combination of sequencing technology and the assembly technique did not yield a contiguous representation of the region of interest in RAC875, we cannot completely rule out additional genes being present. Moreover, we focused our analysis on HC genes while there is a slight chance that an LC gene is expressed in a specific genotype. However, the clear contrast of gene expression between Kukri and RAC875 alleles, and between Drysdale and Gladius (Fig. 5A, C, E), makes *TaSINA* a strong candidate for the QTL effect.

*SINA* genes encode an E3 ubiquitin ligase protein that forms a complex with the E1 ubiquitin-activating enzyme and the E2 ubiquitin-conjugating enzyme which ubiquitinate target proteins for degradation (Mazzucotelli *et al.*, 2006). *SINA* genes were first reported in *Drosophila* associated with the development of photoreceptor cell, R7, localized in the *Drosophila* eye (Carthew and Rubin, 1990). Analyses of the gene family in plants identified several copies of *SINA* in *Arabidopsis thaliana* (Wang *et al.*, 2018), *Populus trichocarpa* (Den Herder *et al.*, 2008), *Oryza sativa* (Wang *et al.*, 2008), *Zea mays* (Wang *et al.*, 2008), *Physcomitrella patens* (Wang *et al.*, 2008), *Medicago truncatula* (Den Herder *et al.*, 2008), and *Solanum lycopersicum* (Wang *et al.*, 2018), with roles in plant development and abiotic stress tolerance (Welsch *et al.*, 2007; Recchia *et al.*, 2013). No *SINA* gene has previously been reported in bread wheat. In Arabidopsis, *SINAT5* ubiquitinates a NAC1 transcription factor to regulate the growth signalling hormone auxin (Xie *et al.*, 2002). Arabidopsis plants overexpressing *SINAT5* had fewer lateral roots compared with the wild type, whereas expression of a dominant-negative mutant (Cys49→Ser substitution) induced more lateral roots (Xie *et al.*, 2002). The ectopic expression of this dominant-negative form of *SINAT5* in *M. truncatula* reduced root nodulation, but both root and shoot growth were more vigorous in young (*in vitro*) plants after 20 d and in older plants after 8 weeks growth (Den Herder *et al.*, 2008). We observed similar results in our experiments when Kukri had fewer primary and seminal roots compared with RAC875 (Table 1): *TaSINA* was more strongly expressed in Kukri compared with RAC875, and early vigour and biomass were increased in wheat plants when *TaSINA* was not expressed (Table 1; Fig. 4). No change in *TaSINA* expression was observed for RAC875, while expression was reduced in Kukri only after prolonged cyclic drought (25 d) (Fig. 5C). This suggests that *TaSINA* expression is relatively insensitive to stress and that heat tolerance in wheat is more likely to be conferred by increases in growth and water use when *TaSINA* expression is reduced. Since *TaSINA* is mostly expressed in roots (Fig. 5A, E), it is likely that increased shoot biomass and water use efficiency are the consequences of changes in the root system due to the RAC875 allele. However, we did not observe major differences in root system architecture and anatomy between Kukri and RAC875 alleles at 21 and 42 DAS. This suggests either that *qYDH.3BL* does not affect root system architecture, or that the sampling time points were not appropriate to reveal a significant allelic effect.

**Fig. 7.**
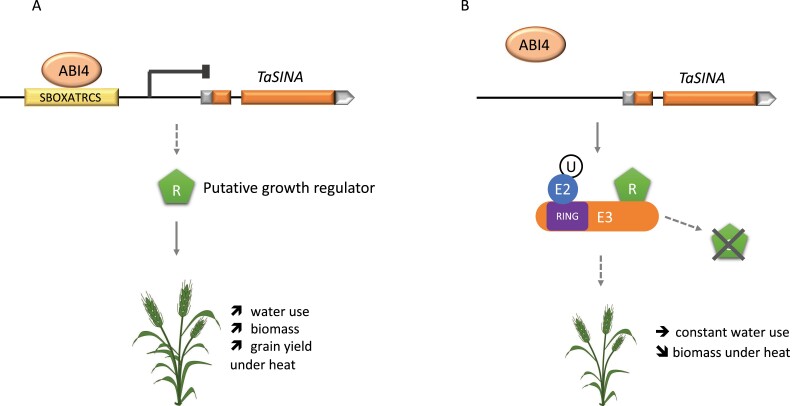
Hypothetical model on *TaSINA* gene function in RAC875 (A) and Kukri (B) wheat varieties. We hypothesize that ABI4 binds to the S box element of the RAC875 *TaSINA* promoter, inhibiting the gene expression, and therefore the degradation of target protein (substrate S) by the E3 proteasome complex. This would enable increasing water use, even under heat, and consequently increasing biomass and yield.

The promoter sequence of RAC875 *TaSINA* contained a *cis*-acting element, SBOXATRCS (CACCTCCA) also called S box, absent in the promoter of Kukri *TaSINA* (Fig. 5G). This motif is a putative ABSCISIC ACID INSENSITIVE-4- (ABI4) binding site which negatively regulates the *Conserved Modular Arrangement 5* gene *CMA5* in Arabidopsis in response to sugar and abscisic acid (ABA) signals (Acevedo-Hernández *et al.*, 2005). ABA is a phytohormone that plays a central role in different physiological processes of plants, mediates gene expression in response to environmental stimuli, and regulates ABA-dependent responses following heat stress (Huang *et al.*, 2016). Sugar signalling also plays an important role in mediating plant responses to environmental stimuli and mediates gene expression associated with photosynthesis, carbon and nitrogen metabolism, and secondary metabolism (Rolland *et al.*, 2002). We hypothesize that the presence of the S box element and ABI4-binding site, in the promoter sequence of *TaSINA* contributes to the down-regulation of its expression in RAC875 and might affect ABA and sugar signalling (Fig. 7A).

The functions of E3 ligases depend on the target they proteolyse. Here we hypothesize that *TaSINA* degrades a regulator of growth (R) (Fig. 7). In RAC875, an ABI4 signal would bind to the promoter and turn off *TaSINA* gene expression, increasing the amount of regulator R (Fig. 7A); this would stimulate the production of seminal roots (Table 1) and vertical root growth (Supplementary Fig. S7), limit fluctuations in water use in response to temperature increase (Fig. 3), and increase biomass (Fig. 4). In Kukri, the absence of the motif would prevent the binding of ABI4 to the *TaSINA* promoter and interrupt the ABA signalling pathway; *TaSINA* would continue to degrade the target protein R, particularly in roots where *TaSINA* is most expressed (Fig. 7B); consequently, plants would grow fewer seminal roots (Table 1), and have a more horizontal root system (Supplementary Fig. S7), increasing water consumption with increasing temperature (Fig. 3) without increased biomass (Fig. 4), decreasing water use efficiency, and leading to an overall loss of yield under heat compared with RAC875. In other words, *TaSINA* might be a negative regulator of plant growth adaptation under heat.

Following genotyping of 743 worldwide wheat accessions, we found that the RAC875 allele was over-represented in the CIMMYT germplasm (Fig. 6B), suggesting that the allele had been selected through breeding for high yield under heat stress. The RAC875 allele was unexploited for wheat breeding until the 1950s, when it appears to have been recruited by the CIMMYT wheat programme in Mexico and into Australia with the introduction of CIMMYT material. Interestingly, among Australian germplasm, we see that Gladius, that was selected for its adaptation to the southern region of the wheat belt, lost the RAC875 allele from its parents Excalibur and RAC875. In constrast, Drysdale, that was selected for its water use efficiency and adaptation to the northern region (with deep soil), still has the RAC875 allele. It seems that Australian breeders have selected unintentionally for or against this allele depending on the local climate and soil depth. It is surprising that the RAC875 allele is not more prevalent in India and other Asian countries given that CIMMYT germplasm, either as parents or directly as cultivars, has been estimated to account for >65% of production in India. This allele has not yet been exploited in other regions where heat was not considered a threat until recently. With the increasing occurrence of heat stress events, this allele could be beneficial in other regions with deep soil such as in Europe and in the Punjab in India.

## Supplementary data

The following supplementary data are available at *JXB* online.

Fig. S1. Air temperatures during 2014 and 2015 experiments using the RAC875×Kukri and Drysdale×Gladius RILs in the deep soil platform in a polytunnel.

Fig. S2. Average soil water tension during the grain-filling period when drought was imposed in the deep soil platform.

Fig. S3. Example of the randomized design developed for the deep soil-mimic wheelie bin experiments showing one of the bins in 2017 with parental lines RAC875 (allele A) and Kukri (allele B) and NILs containing either allele.

Fig. S4. Wheat *TaSINA* gene sequences from Kukri, RAC875, Drsydale, and Gladius generated by Sanger sequencing using primers designed for TraesCS3B02G572900 of Chinese Spring RefSeq v2 and described in Supplementary Table 3.

Fig. S5. Wheat TaSINA gene sequences retrieved from the 10+ Genome dataset https://wheat.pw.usda.gov/blastn) using the Chinese Spring TraesCS3B01G572900 gene as query.

Fig. S6. Genetic diversity around the *qYDH.3BL* QTL visualized in DAWN. 

Fig. S7. Single-marker analysis of 44 RAC875×Kukri RILs phenotyped in the deep soil platform sown in August 2014.

Fig. S8. Expression profiles of high confidence genes annotated in the interval delimited by ADW594 and ADW577 on chromosome 3B.

Fig. S9. Expression profiles of low confidence genes annotated in the interval delimited by ADW594 and ADW577 on chromosome 3B.

Fig. S10. Expression of seven genes annotated in the interval delimited by ADW594 and ADW577. 

Fig. S11. Amino acid alignment of the *TaSINA* gene in RAC875 and Kukri and its homeologues on chromosomes 3A and 3D with *SINA* genes from barley (AK), *Triticum urartu* (TRIUR), and *Aegilops tauschii* (XP). 

Fig. S12. CLUSTAL multiple sequence alignment of *TaSINA* gene sequences of 14 wheat varieties by MUSCLE (v3.8, EMBL-EBI). 

Table S1. Descriptions of the environment in Ciudad de Obregon (Mexico) where the RAC875×Kukri RILs field trials were conducted, showing water supply from rainfall and/or irrigation, and RAC875×Kukri DH and RILs.

Table S2. Country of origin, year of release, pedigree, and genotyping at *qYDH.3BL* of 788 wheat accessions.

Table S3. Primer sequences for KASP genotyping, PCR, qPCR, and Sanger sequencing.

Table S4. ANOVA comparing agronomical and physiological traits in August and September 2014 experiments on 44 RAC875/Kukri RILs.

Table S5. ANOVS between sibling lines for root and shoot traits measured in RAC875/Kukri NILs grown in pots in the glasshouse in 2017.

Table S6. Analysis of root anatomy traits measured at 21 DAS in NILs and parental lines, RAC875 and Kukri grown in pots in a glasshouse. 

erab044_suppl_Supplementary-Figures-S1-S3_S6-S11_Tables-S1-S4-S6

erab044_suppl_Supplementary-Figures-S4-S5

erab044_suppl_Supplementary-Figure-S12

erab044_suppl_Supplementary_Table-S2

erab044_suppl_Supplementary_Table-S3

## Data Availability

Materials used and described in this paper are available for non-commercial research purposes at the School of Agriculture, Food and Wine (University of Adelaide). The sequence data associated with the paper are available in GenBank, accessions numbers MW770316, MW770317, MW770318, and MW770319.

## Abbreviations

DH: doubled-haploid
HC: high confidence
IWGSC: International Wheat Genome Sequencing Consortium
KASP: kompetitive allele-specific PCR
LC: low confidence
NIL: near-isogenic line
QTL: quantitative trait locus
RIL: recombinant inbred line
SINA: seven in absentia
SNP: single nucleotide polymorphism
WGS: whole-genome sequencing
