# Supplementary material for: The wheat Seven in absentia gene is associated with increases in biomass and yield in hot climates
Source: J Exp Bot. 2021 Feb 5;72(10):3774–91. doi: 10.1093/jxb/erab044 (PMC8096608; doi:10.1093/jxb/erab044)
Supplement: erab044_suppl_Supplementary-Figures-S1-S3_S6-S11_Tables-S1-S4-S6 [file erab044_suppl_supplementary-figures-s1-s3_s6-s11_tables-s1-s4-s6.pdf]

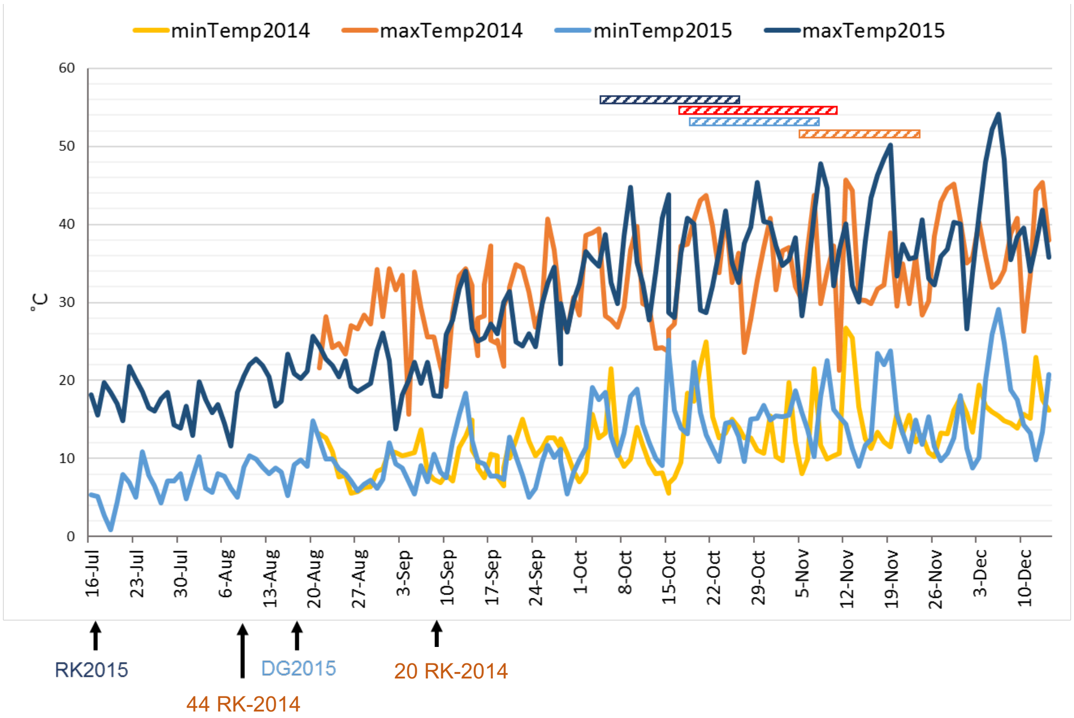

Supplementary Fig. S1. Air temperatures during 2014 and 2015 experiments using the RAC875 x Kukri and Drysdale x Gladius RILs in the deep soil platform in a polytunnel. Black arrows indicate the sowing date for each experiment and lines. In 2014, 44 RAC875 x Kukri RIL (RK) were planted in August and 20 RK in September. In 2015, 30 RK lines were planted in July and 20 Drysdale x Gladius RIL (DG) in August. Flowering periods have been highlighted for each experiment: red for Aug-2014; orange for Sept-2014; light blue for DG2015; dark blue for RK2015.

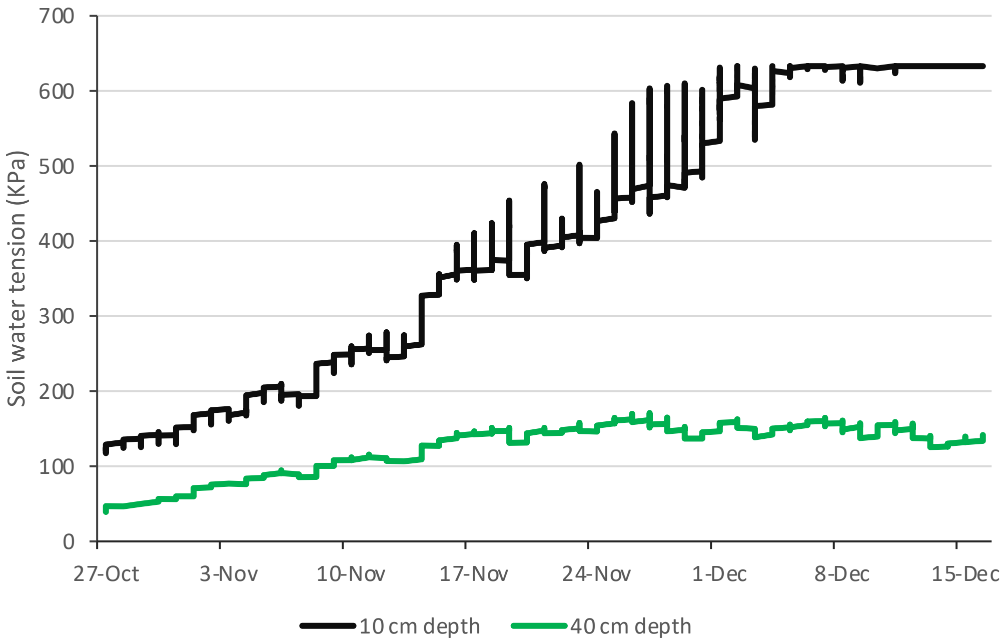

Supplementary Fig. S2. Average soil water tension during the grain-filling period when drought was imposed in the deep soil platform. RAC875 x Kukri RILs and parental lines were planted in August in three bins.

NIL 5A	NIL 2A	NIL 3B	NIL 3A	NIL 5B
NIL 1B	NIL 5B	NIL 5A	NIL 1A	NIL 4B
NIL 3B	NIL 2B	NIL 2A	Kukri	NIL 4A
NIL 4A	NIL 3A	NIL 2B	NIL 4B	NIL 5A
RAC875	NIL 1A	NIL 1B	NIL 4A	Kukri

Supplementary Fig. S3. Example of the randomized design developed for the deep-soil mimic wheelie bin experiments showing one of the bins in 2017 with parental lines RAC875 (allele A) and Kukri (allele B) and NIL containing either allele.

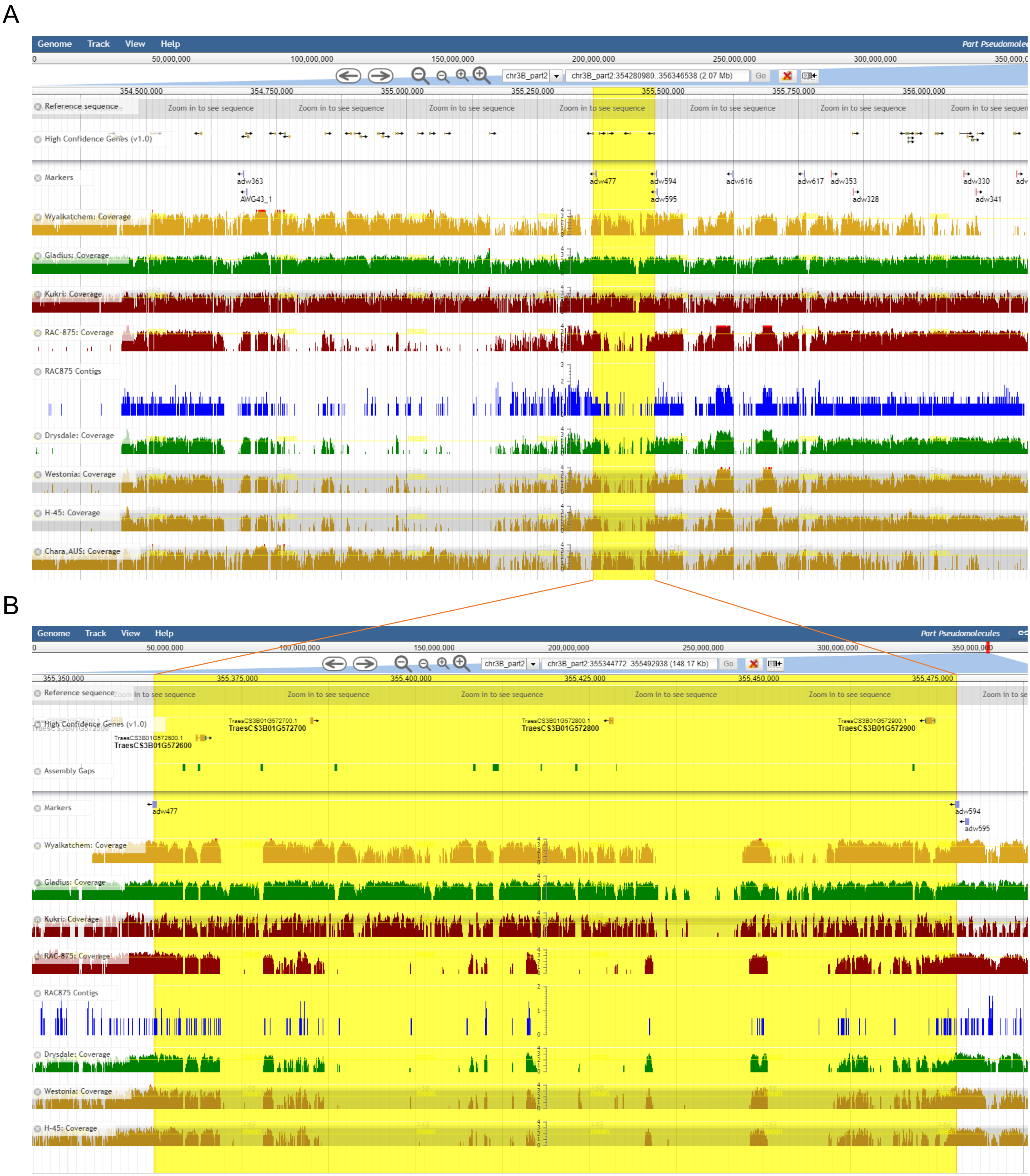

Supplementary Fig. S6. Genetic diversity around the *qYDH.3BL* QTL visualised in DAWN[75]. (A) The QTL is located between markers adw477 and adw594 and highlighted in yellow. Read coverage is displayed for eight wheat accessions with Kukri and RAC875 in dark red, Gladius and Drysdale in green. The track in blue represents the minimap2 alignment of assembled RAC875 contigs to the reference genome. (B) Close-up of the QTL showing the sparsity of reads from RAC875 and also RAC875 derived contigs. A track showing the location and relative size of assembly gaps has been added (3rd from top).

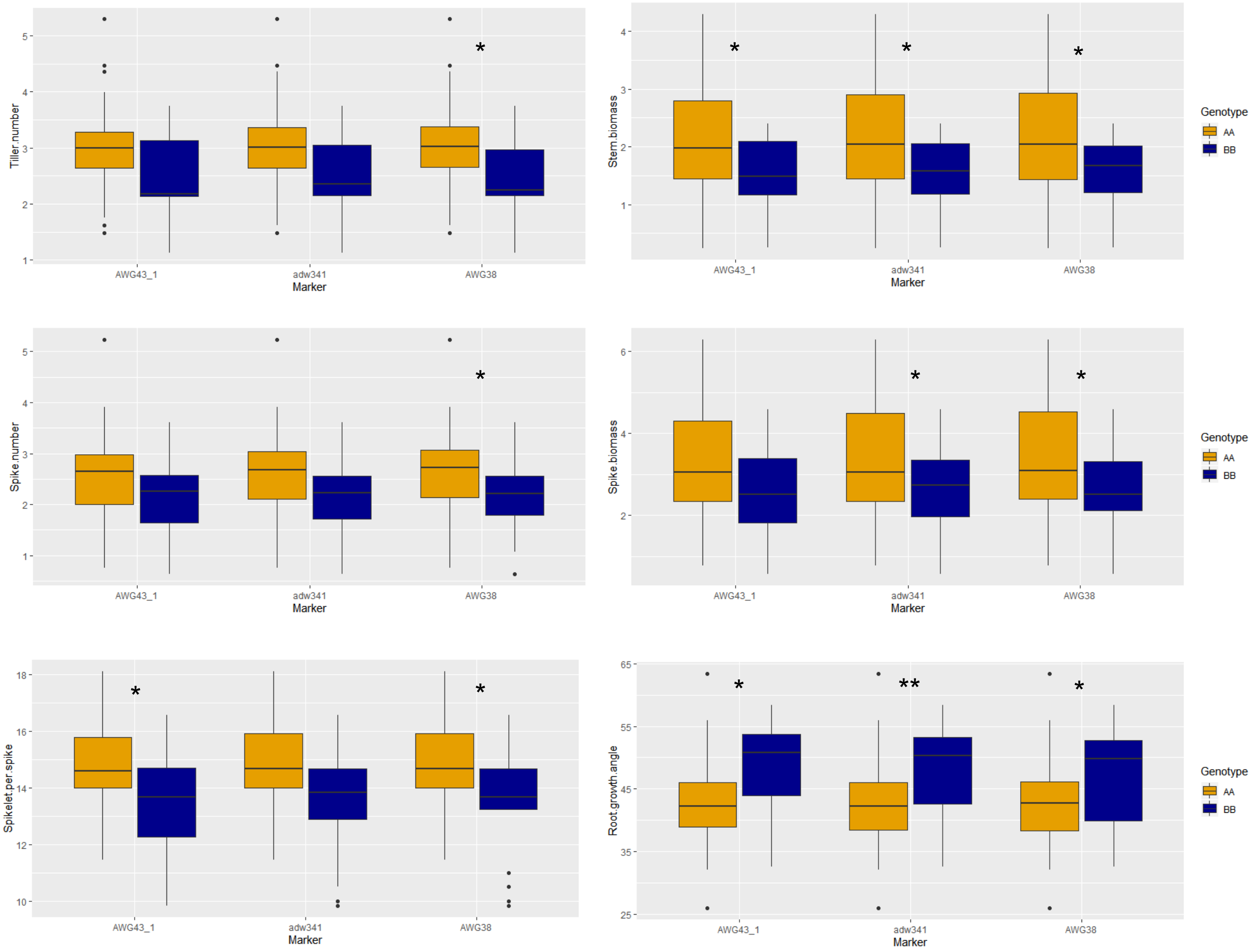

Supplementary Fig. S7. Single marker analysis of 44 RAC875 x Kukri RILs phenotyped in the deep soil platform sown in August 2014. AA: RAC875 allele; BB: Kukri allele. Anova of AA vs BB allelic effect with * : p value < 0.05; ** : p value < 0.01.

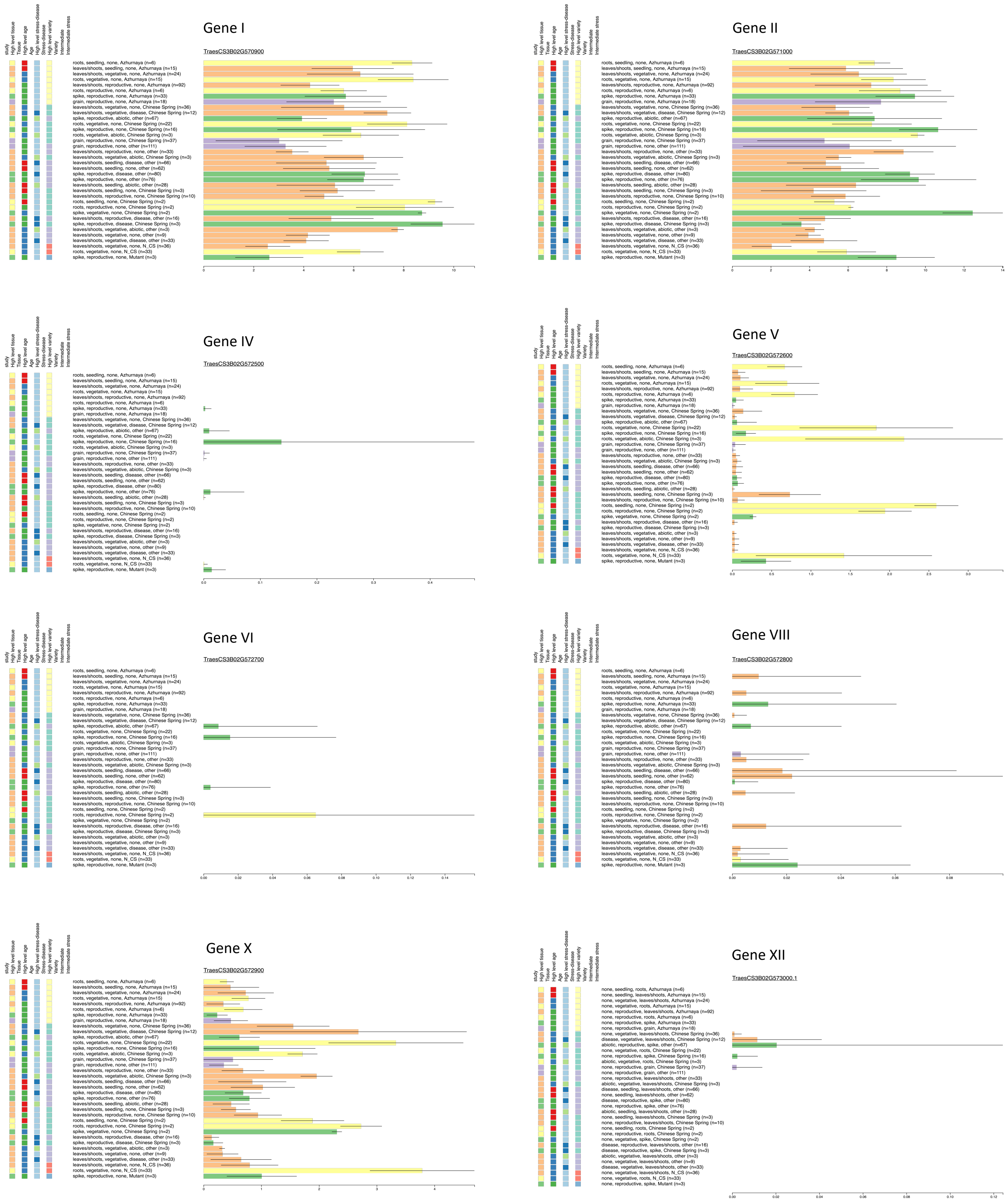

Supplementary Fig. S8. Expression profiles of High Confidence genes annotated in the interval delimited by ADW594 and ADW577 on chromosome 3B. Data were extracted from the wheat expVIP platform including 36 datasets (version Dec 2020).

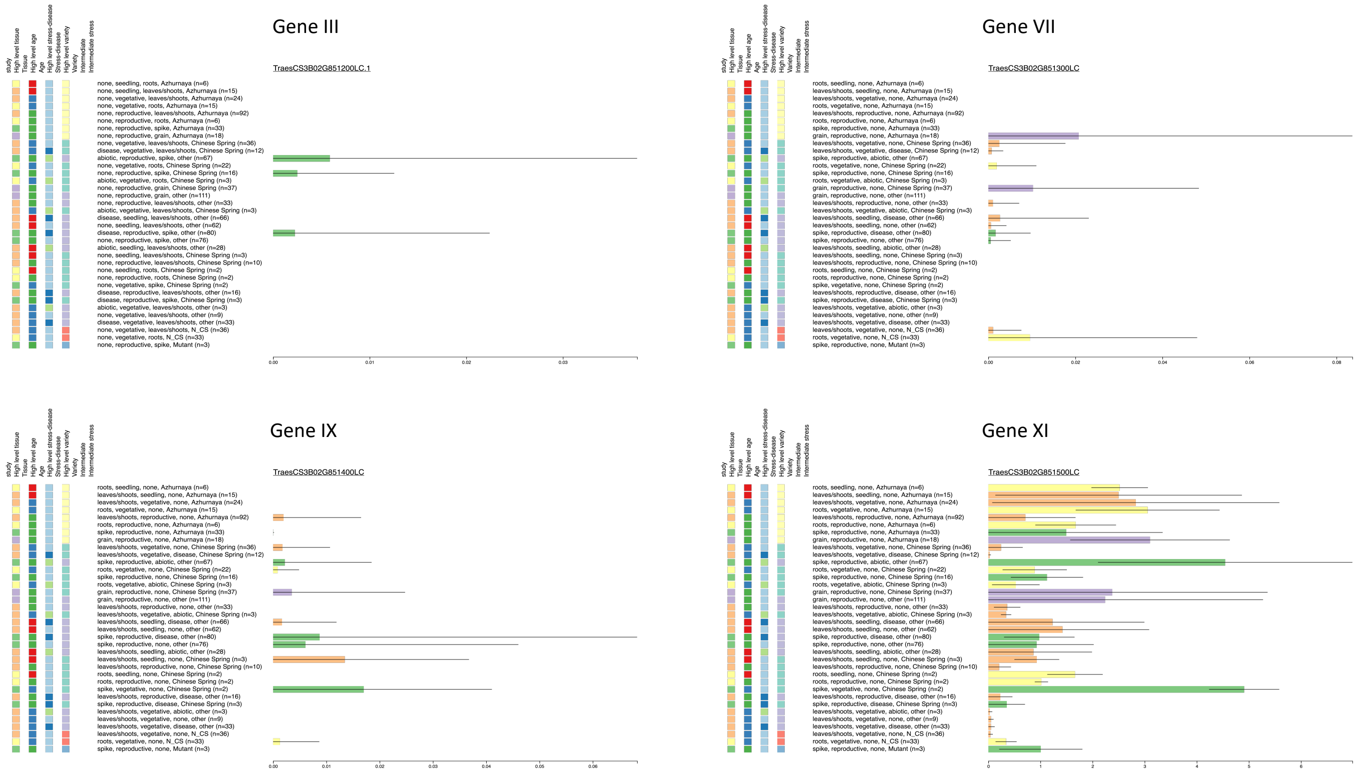

Supplementary Fig. S9. Expression profiles of Low Confidence genes annotated in the interval delimited by ADW594 and ADW577 on chromosome 3B. Data were extracted from the wheat expVIP platform including 36 datasets (version Dec 2020).

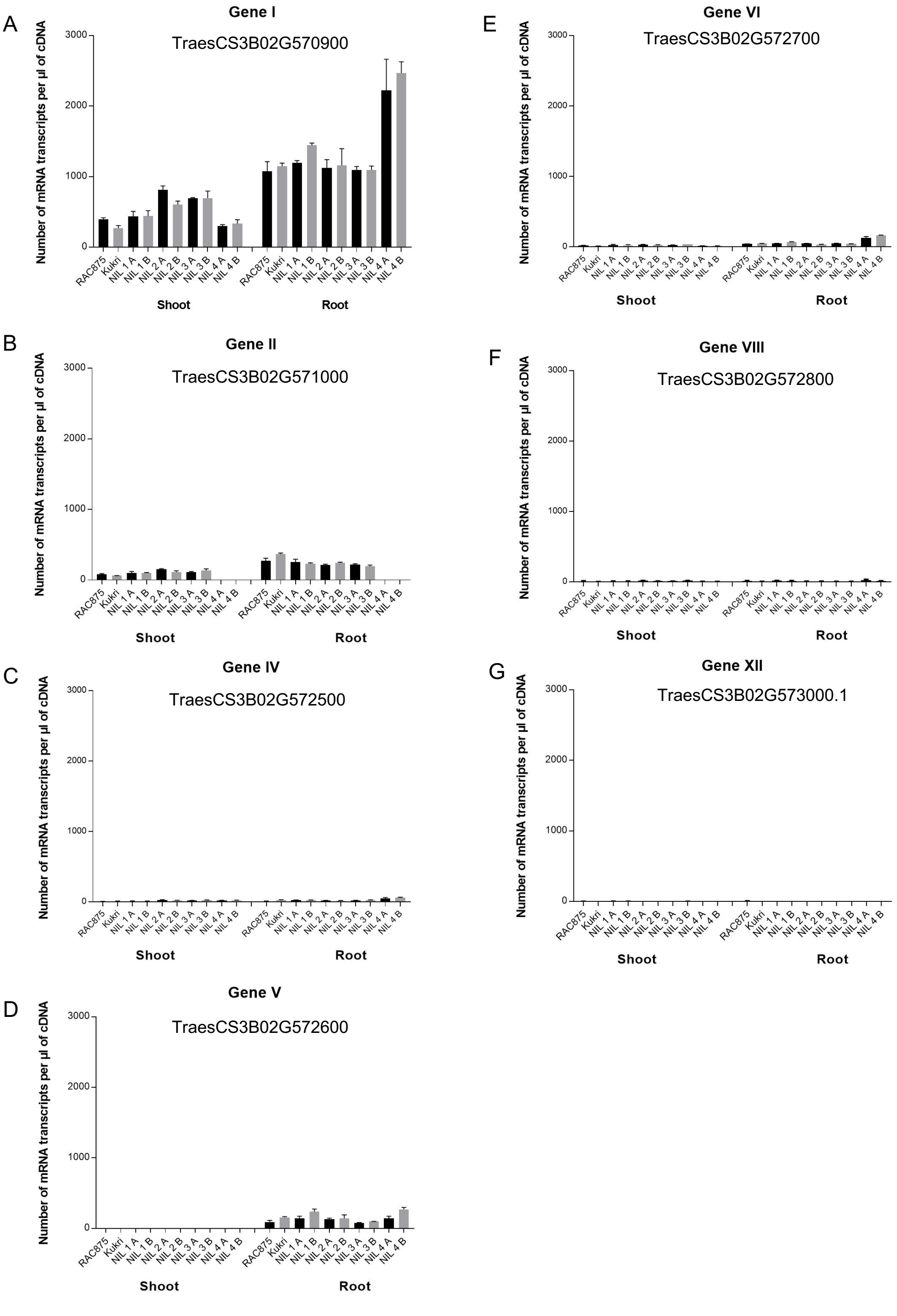

Supplementary Fig. S10. Expression of seven genes annotated in the interval delimited by ADW594 and ADW577. No significant differences in the expression profile of these genes could be found between parents and among the NILs. “A”: NIL contains the RAC875 allele; “B”: NIL contains the Kukri allele.

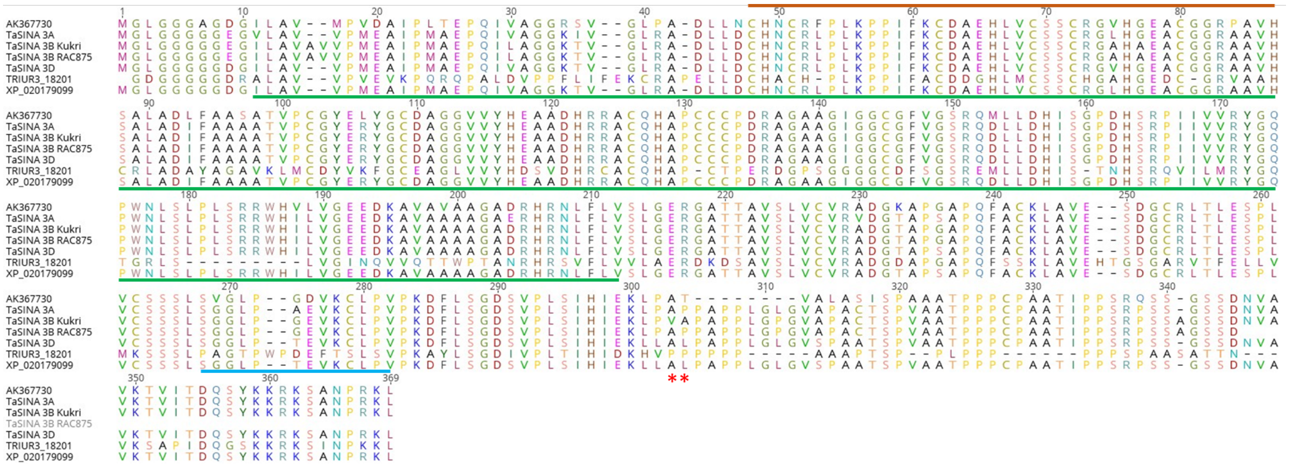

Supplementary Fig. S11. Amino acid alignment of *TaSINA* gene in RAC875 and Kukri and its homeologues on chromosomes 3A and 3D with SINA genes from barley (AK), *Triticum urartu* (TRIUR) and *Aegilops tauschii* (XP). The conserved domains were identified and highlighted: the orange line indicates the position of the conserved amino acid in RING finger domain; the green line indicates the position of the TRAF-like domain of the Seven in absentia homolog family; the blue line indicates the position of a nuclear localization signal; the red star indicates the amino acid substitutions between *TaSINA* in Kukri and *TaSINA* in RAC875.

Supplementary Table S1. Descriptions of the environment in Ciudad de Obregon (Mexico) where the RAC875 x Kukri RILs field trials were conducted, showing water supply from rainfall and/or irrigation and RAC875 x Kukri DH and RIL (from Bonneau, 2012).

Environ-ment [a]	Latitude	Longitude	Altitude (M)	Lines tested [b]	Sowing density seed m^{-2}	Rainfall and/or irrigation mm	Mean temperature around flowering time [c]	
							**Sept**	**Oct**
11_DI_CS	27˚28' N	109˚56' W	38	34 DH/77 RI	200	150	24.5	29.3
12_DI_CS	27˚28' N	109˚56' W	38	27 DH/ 40 RI	200	100	24.7	26.6
							**April**	**May**
11_FI_LS	27˚28' N	109˚56' W	38	34 DH/ 109 RI	200	1050	30.7	34.8
12_FI_LS	27˚28' N	109˚56' W	38	27 DH/ 40 RI	200	900	30.5	37.8

[a] Mex Mexico, DI drip irrigation, FI for flooding irrigation, LS for late sowing, CS for conventional sowing date.
[b] DH doubled haploid lines, RI recombinant inbred lines.
[c] The temperature data for Mexico were collected at www.agroson.com.mx

Supplementary Table S4. ANOVA comparing agronomical and physiological traits between August and September 2014 experiments on RAC875/Kukri RIL and four parents (n = 48 lines for August and n = 24 lines for September).

Phenotypic traits	Mean Aug.	Mean Sept.	p value α = 5%
Early vigour	0.65	0.58	0.04
Flag leaf length	15.15	11.14	5.25E-10
Flag leaf width	1.48	0.99	2.62E-12
Stomata density	23.52	24.37	0.21
Spike length	7.63	6.26	2.03E-05
Spikelet per spike	14.55	12	2.34E-05
Spike number	2.44	2.31	0.51
Spike biomass	3.13	2.04	0.0004
Stem biomass	1.96	0.97	7.25E-06
Tiller number	2.82	2.03	0.0002
Total grain	57.07	35.18	0.0003
One grain weight	36.62	32.28	0.003
Harvest index	0.29	0.28	0.3
Crown and brace roots	0.93	1.81	0.0002
Number of seminal roots	4.22	4.35	0.67
Root growth angle	44.45	44.85	0.83
C amount	1252	1024	1.28E-08
N amount	102.19	78.38	1.46E-10
d13C	-23.15	-23.95	2.01E-09
d15N	-3.097	-2.806	0.17
d%13	15.51	16.02	0.08
d%15	3.11	2.82	0.16
%C	42.45	43.58	0.13
%N	3.48	3.31	0.03
[C]/[N]	12.39	13.14	0.01

Supplementary Table S5. Analysis of variance between sibling lines for root and shoot traits measured in RAC875/Kukri NIL (here called HIF from heterozygous inbred family) grown in pots in glasshouse in 2017. Traits were measured per plant at 21 and 42 DAS (n=6 plants per line). AA: RAC875 allele; BB: Kukri allele.

	21 DAS							42 DAS					
	Early Vigour	Dry root biomass (g)	Dry shoot biomass (g)	Total root length (cm)	Total root area (cm^2)	Total root volume (cm^3)	Number of root tips	Root biomass (g)	Shoot biomass (g)	Total root length (cm)	Total root area (cm^2)	Total root volume (cm^3)	Number of root tips
RAC875	25.31	0.06	1.61	1069.67	153.09	2.58	804.83	0.66	2.87	2187.51	1293.27	21.38	7847.8
Kukri	25.58	0.07	1.88	1024.61	149.63	2.48	932.33	0.66	3.48	2237.9	1314.73	19.47	8517.2
T-test	**0.9**	**0.5**	**0.31**	**0.82**	**0.89**	**0.85**	**0.41**	**0.9**	**0.27**	**0.86**	**0.9**	**0.51**	**0.56**
HIF1-AA	26.59	0.06	1.87	998.63	142.05	2.13	921.5	0.74	3.86	2501.01	1477.98	24.19	8938.2
HIF1-BB	28.44	0.05	1.81	1008.31	145.63	2.22	831.67	0.62	3.38	2301.35	1351.45	21.5	8210.5
T-test	**0.22**	**0.52**	**0.82**	**0.93**	**0.84**	**0.76**	**0.4**	**0.3**	**0.28**	**0.58**	**0.54**	**0.5**	**0.5**
HIF2-AA	19.17	0.05	1.15	851.68	141.43	2.33	582	0.69	3.43	2074.47	1235.68	18.66	7561.7
HIF2-BB	22.36	0.06	1.62	832.61	143.05	2.42	625.17	0.61	3.71	2161.51	1274.81	19.65	7562.2
T-test	**0.57**	**0.4**	**0.26**	**0.92**	**0.96**	**0.8**	**0.79**	**0.65**	**0.68**	**0.81**	**0.85**	**0.75**	**0.99**
HIF3-AA	23.23	0.06	1.91	1122.94	174.6	2.82	849.83	0.52	3.08	1916.83	1145.45	16.48	8427
HIF3-BB	20.05	0.05	1.48	982.58	156.15	2.45	706	0.6	3.66	2262.58	1354.65	20.82	8024.8
T-test	**0.06**	**0.25**	**0.05**	**0.34**	**0.34**	**0.17**	**0.24**	**0.56**	**0.4**	**0.46**	**0.45**	**0.37**	**0.79**
HIF4-AA	24.09	0.06	1.73	1021.11	162.81	2.65	771.17	0.4	2.97	1446.82	867.02	12.8	6588.3
HIF4-BB	22.06	0.05	1.83	898.2	143.41	2.37	742	0.57	3.03	1675.76	1002.51	14.72	7386.5
T-test	**0.31**	**0.16**	**0.62**	**0.2**	**0.16**	**0.24**	**0.73**	**0.1**	**0.9**	**0.51**	**0.5**	**0.58**	**0.57**

Supplementary Table S6. Analysis of root anatomy traits measured at 21 DAS in NIL (HIF) and parental lines, RAC875 and Kukri grown in pots in a glasshouse. Cross sections were done on six plants with three technical replicates per plant. Samples of seminal roots were collected at two positions: 5 cm away from the tip (TIP) and 2 cm away from the root-shoot junction (RSJ). AA: RAC875 allele; BB: Kukri allele; ns = non-significant; CMX = central metaxylem vessels.

	RSJ							TIP						
	Root diameter (µm)	Stele diameter (µm)	CMX diameter (µm)	Protoxylem diameter (µm)	Thickness cortex (µm)	CMX number	Protoxylem number	Root diameter (µm)	Stele diameter (µm)	CMX diameter (µm)	Protoxylem diameter (µm)	Thickness cortex (µm)	CMX number	Protoxylem number
RAC875	519.22	221	48.31	12.96	298.22	1.69	9.25	480.75	193.06	56.68	12.63	287.69	1.14	11.47
Kukri	515.58	224.64	56.19	13.24	290.94	1.14	13.17	427.14	177.89	55.90	13.81	249.25	1.08	14.33
T-test	**0.87**	**0.7**	**0.03**	**0.53**	**0.75**	**0.01**	**0.02**	**0.22**	**0.24**	**0.89**	**0.15**	**0.25**	**0.66**	**0.04**
HIF1-AA	517.06	243.14	50.88	15.46	273.92	1.97	10.56	436.25	178.67	54.13	15.03	257.58	1.08	10.50
HIF1-BB	517.67	221.22	53.11	15.26	296.44	1.44	8.72	474.94	180.97	57.24	16.29	293.97	1.17	11.11
T-test	**0.99**	**0.14**	**0.48**	**0.83**	**0.44**	**0.25**	**0.17**	**0.3**	**0.81**	**0.51**	**0.02**	**0.27**	**0.67**	**0.57**
HIF2-AA	507.39	225.28	51.64	15.03	282.11	1.56	10.89	511.36	180.02	56.96	13.78	331.33	1.08	10.33
HIF2-BB	535.52	230.61	46.78	13.17	304.92	2.00	10.14	518.83	191.81	55.69	12.22	327.03	1.61	10.14
T-test	**0.33**	**0.69**	**0.4**	**0.11**	**0.36**	**0.26**	**0.56**	**0.85**	**0.41**	**0.81**	**0.11**	**0.91**	**0.16**	**0.89**
HIF3-AA	506.4	219.7	49.25	15.71	286.7	1.63	7.53	447.83	180.25	66.25	15.78	267.58	1.33	7.94
HIF3-BB	441.42	194.66	47.63	14.63	246.75	1.42	10.00	413.86	179	52.5	13.93	234.86	1.22	10.86
T-test	**0.08**	**0.16**	**0.75**	**0.31**	**0.09**	**0.44**	**0.04**	**0.4**	**0.91**	**0.001**	**0.07**	**0.44**	**0.59**	**0.03**
HIF4-AA	479.64	208.56	46.9	12.8	271.1	1.56	10.33	438.36	177.53	63.33	14.42	260.83	1.06	10.31
HIF4-BB	519.36	223.64	47.64	14.14	295.72	1.69	10.08	440.47	185.33	61.25	14.01	255.14	1.25	10.08
T-test	**0.13**	**0.25**	**0.73**	**0.2**	**0.19**	**0.54**	**0.84**	**0.95**	**0.41**	**0.66**	**0.63**	**0.85**	**0.09**	**0.88**
