# Supplementary material for: The wheat Seven in absentia gene is associated with increases in biomass and yield in hot climates
Source: J Exp Bot. 2021 Feb 5;72(10):3774–91. doi: 10.1093/jxb/erab044 (PMC8096608; doi:10.1093/jxb/erab044)
Supplement: erab044_suppl_Supplementary-Figures-S4-S5 [file erab044_suppl_supplementary-figures-s4-s5.pdf]

Supplementary Fig. S4 Wheat *TaSINA* gene sequences from Kukri, RAC875, Drsydale and Gladius generated by Sanger sequencing using primers designed for TraesCS3B02G572900 of Chinese Spring RefSeq v2 and described in Supplementary Table 3. The fasta sequences include exons and introns.

>Kukri TaSINA (4,812-4,814..Start codon) (4,812-4,985..exon 1) (5,093-5,934…exon 2) (4,696-4,811..5' UTR)

ATAATGAGTTATCACTCCCTATTTGGGTGAATCACAAAAGTTTAAATTAAGAACAATCCATTCCATCAAACTTTGAT
AATATTAGCTATTAATGTATAAAGATTAATACAATTATAGCTTTATTCGGCATGGATGCTATTCCATAGTACCATTAT
TCATAATATTATATTGAGTTAAACATACTGTAGATTATCCCAAATTGTAAACAACTTTAATAATAATACTCCCTCCAT
TTTTATATACAAGGCCACTATGGAATATACATTTTGCATATATATAAGGCCACCAACAGTAATCGAGGCAAATTTAA
TGATATTTTCTCGTACTAATAACCTGTTTAATACTTGCATGCTTGCAATCATAATGACAGTCAGCTACTTCCTCCACT
CAGTTTTTTTGCATGCATGTGGAGTATTAATGATCCCAGTAAACAAGAAGAAAAGTTGACTTGTAAAGCAGGCATT
AAATTTTACATTGGTACCTGTAATCCAAGTTTGTGGCCTTATATACAAAAATGGAGGGAGTATAAAAATCTAGCAG
CGCAAATGCGCGGATAACTGTGCTAACTACTCAATAAGAGGTTAAAAGAAGATTCACATGCACAACTGTAGAAAA
CAAAAGGCCACACAACCAGTATTTAAAAAATGTACATTAATTGGACTCCCTTCATTCCTACTTACCATGCACATATTT
TTTTCTAGTTTTTCGTGATTATCTAGGGTATTATGGTTCCCACACAGATGATTCTCTATCTTTCCAACCCTTATTTTGG
GTAGTACTACCAATGCATATATTATCAATGTTCTTCCCATAAAATGCTCCCGTGACATTAACTATCCCACCATGCATG
GAGGAAATTAATGCATGCATATCATTAACTATCTCAACGTCTAAACCGAAACAAGGCACCGCCAGATGCCGAGGG
CAAGCTCGTCCAACTACACATCCAATTGTCAATCTCCACCACTCATTTATATGCGGGCTTGAACTAAAAAAAATTAG
ATACTCCCTCCGTCTAGGTGTGTAAGTCATCTTACGAAAACCAAATAATCCCAAAATAATTAGGCGTGGTGCATTAA
CTTCTACCTCGTTTCTTGTTTTTTGACATATCAACCAATAATAGATGAGGGTGTGCATGCTTTTAATGACTTGAGACT
ATTAAACACGACATGCAGTGGTTAGTTCATTGCATGCAATACTATTAATTAGCAAATAAACATTAATGTCTCTCATT
TTCCCCTCCTCCTTGGTCACGGTGCACAGCCTAAGATGACTTACTCACCTAGACGGAGGGAGTACTCACTTCAGAT
GGGAATACAAGTCGGGCACAGTTTCTAGGTCTTCAATTTGACAAGCGAATAATATGCCACAAAAAAATGGTAAGA
AACCTCACCTGGCAAGGAATATACATTTTTTGTGGCATATAACATATATTTTGCTTGTCAAATAGATAACCTAAAAA
TCGGTGTCTGTGTATATTCCCACCCAAAGGGAGTAACAATTAGGAATTAAGAGAGCTTTTATTATGGTAGACCCTT
CTTTCATTAAGATGCCCACGTCTAAGGACATCATCAATGTATCATTCTTAAGTCGGTAATTAGCTGGGGCCACACTA
TGCTATAGCAAACTCGAATTCCTCCTATGCTTAATTCCAATATGGCTATGGACGTTAGGGTGAAGCCCACTTGCCG
GTATTTTCTCTTGTAGTTTTAACCCCTCAAATCATGTGAGGGGAGAAAGAAATAGTTTTTGATTCCTTGTGGCATGG
CCTTAGCACTATGACCGGTCTTAAAGATTTTTGTTGCTAGGACCCCTTCCCACAATGCTTGAGGATATGTCATTAGA
CCCATATTTTTGCAACAATGGAGACATAATATAAGATTTGATGATATATAGGTTATATTTCATTAGAGATGATCTCT
TAGGCCTGTCTCGATGGATATTATCATAAACTATTATCATTAAATGATGCTACTTTGCTTACTACATTCCCATCATTT
CATAGCATCATAACATATATTTTACTGGGAATAAACTTATATTTTATAACTTAAAATTATTATCAAAATGTGACAAAA
TACAAGAGGACTAGTGAATCAGGACGGAGGCGGTAGGAAAAGAGACAGTGTCACGGTGGAGCATCGGGAGAGA
GAGTCCCAACAGTAAAAAGCCAAACTCACTGAAAAGTAAATGATTAAAAAGAAGATTCACATGCCCACTTGCAGTA
CTACCAAGCAAAAACACAAGGCCAATCGACCAGTAATGTATAATTAAAAACATTGTCACGGTTCCCCAGTTGAAAT
TTTTTGGGTCATGGTGTTCAACAGAGTTATTTAATGTTCTACTAGAGGGCATTAATGTGACATATCGTAGTTTAATC
GCGGTGGCGTGGCAATGACATTGGCAAGCAAACTAAACTAGCTGAGTGTCCTTGTCCTCTCCACCCACCACTAGTC
AAGCCTAATTAGCTAAGCTGCGTTGGAGAGGGCTAAAAGTGAGTCTCTCTGGACCTGGAATCCAAACATTCAAAA
ACGATTTGGTGAGAGCATCTCCAGTCGCATCCCCAACACGGCCTTCCAGGCGATTTTTTCGTGCCGGCGCCTAAAA
ATGGCCCAGTCGCGTCCCCAGGAGCCCGTTTTTCACCGGCTTGGGCCGAAATCAATACAGGCGGACCCAGGCCGA
ACCCGGCGCGCTGGGGGCGCCGGGGCGAGTGGTTTTGGCGCGAAATAGCCCCGGGCCCACCGAGTCTGCGAGAC
GGCCGCTTCGTCGCCCTCATCGCCTTGGTTCCCACGGGAATCAATGCCAAGACTGCCACGATGCCGTCGCCGGTCA
GCCTTGCCATTGATAGCTCATCACGGGCGGCGCGTCACGGGTGGCGCGGCGACGCCTCCCCTCCCGCCACGCGTA

CACACGGGCGCGACTATATAAGCCGGTGGCCTCCCTCACCTTTGGCCACACCAGCCCTCGCCGTCCATCGACCGCC
GTCGAGCTCTTCCTCTCCCGTGCGCAGCCGCGACGCTTCTTCCCTCCCTCTCCCTCACTCCCAAGCCCGGTGGCCGC
GCGTTTCCCCGGCGACGGCCAACGGCTTCGGTCACCGCTCGCTTCACGAGTGGGAGGGTGGCTCCTGTTCGAGGC
GAACATCCCGGCGCCACCGGACATGCGCGCATGAACGATGGGGTGGAGACTCGGCAACGGGGGAGTGCCCATTC
CCCCCGGTGCCCGACGTCGAGGCGTGCCCCGCCTTCTTCGCCGCCGAGGTCGACCGCGTGCAAGCCTCCCTCACAG
CGGAGCAACTCGCCCCCCACCAGTACGCCGTCGAAAACCACGCGGCGTGGGCGGCGTACTTCCAGCACCGCCAGG
AGCAAAGACTGGCGTCCACCAACGGGGCGCCGATGGTGGGCAGCGTCAAGAACAGTGAGGGGCACCGCGTGTG
GTGGGGCGCCACCGAATATGCTCGACGGCGTGCTGTCGTACCTCGAGGGCGGTAACGAACCGTCGCTGGCATACC
GTCCCATGGCGGCCGCCCCGGTCCACCGCCGGCACGCGGGGCAATGGATGCCCAGGAGGTTCCGTTCCTCCTCGA
CTTCCTCCTCGCACTCCTCCTCCCGTTCTTCTTCCCACTCCTCCGGCTCGACGACGTTGCTCGGCGTCAAGGCCGAGC
CCGCAGCGGAGACGCTGCTCAGCCGGCGCACTCGCAGCGTCGGCATCATCATAAACGAGGGCGGCCGGCGCTCC
TCCTCATCGGCTCCTCCGCGCTTCGTCAAACCAAAGACGGAACCGAGGCTCGCGGCCGTGAAAACGGAGCCGGGG
CTCCCCGCCGTGAAGACAGAGCACGGCGACGTGGAGCTCGATGAGGAGGCGACCCTAAAATGGGCGCGCGCCGA
CTGGCTCAAGATGGAGTGGGAGCGCCAGTGCGCCGCCCTGCAGCACTACGAACAGCGCCGCCAGGGCCGCGACG
AAGGAGGCGTCGTCATCCTCAACGACAACGACGACGACGACGCGCCGCCACCGCCACCGGTCCGCCATGGCGAC
GCCGGGCAAGGGTCCAGCGGGGGCGGCCGCGTCAAGGAGGAGAAGACCGCCGCCGACGACGACGCGCCGCTCC
ACCGGTCCGCCATGGCGACGCCGGGCAAGGGTCCAGCAGGGGCGGCCGCGTAAAGGAGGAGAAGGCCGCCGCC
GACGACTACGCCGCGTTCAGCGACTTCTTCGCACCTTCGTTGTAGTTTCGGCTTTTTTGAGATAAATTTGCTATGTA
AACTGTCTTCTTTGTGTAATATATGTCCAGTTTACCGAATTTAGCCGAACTTTATTGAATGTTTTTTTTAATTTAGAC
GGCGTCTGGGAACGGTCATGGGATCGGCGACTGGGAATCCAGTTGCCTTAGGCGGCGATTTTACGCGCCCCTTGG
GGAACGGCTGGAGATGGTCTGAGTCTCCTTGATGCCCGAGCTCTCGGATGAATGGCAGATTTCTTTTGAATGTTGC
AGTTGGGTGAATGCGGTTAGGTTAGGTGGATGGTAAAAAGTAAAAACAACTTCTATTCCCAAAGTCTGAACCCGT
CAAAATCTGGCTATGGGCACGGCCCCATCTCATCAGAGACCCCACCAGACCTCCTGTGACGGGGCTCGCACCCGAT
ACCATCTTGATCGGATCCATACCCCTTAACCCCCTTGACCTCACCTTCGCTCACAAGCGCCAGTACACAGAGCAGAG
AGCCGCCCGCCTGAGTGCTACCCGCCGCTGCTGGTGCCAACAGAGAGCCGCCCGCCCGCCCGCCCGCCTGACTGA
ATCTTGGTGGGGGAGGAGAGGAGAGATGGGCCTCGGCGGCGGCGGAGAGGGGATCCTGGCCGTGGCCGT
GGTGCCCATGGAGGCCATCCCCATGGCGGAGCCGCAGATCCTCGCCGGCGGCAAGACCGTCGGCCTGCGGGCGG
ACCTGCTCGACTGCCACAACTGCCGCCTCCCCCTCAAGCCCCCCATATTCAAGGTGAGATCCGCCGGCCGCCCCTCT
TCCCCAAGATTCATTTTTCCGTTTTCTTGAATCTGGCACGGCGTGACCGAGCGTATCTGGTGTGCATGAATCTTGGC
CGGCAGTGCGACGCCGAGCACCTGGTCTGCTCCTCCTGCCGCGGCGCCCACGCCGAGGCCTGCGGCGGCCGCGCC
GCCGTCCACTCCGCGCTCGCCGACATCTTCGCGGCCGCCGCCACCGTGCCCTGCGGCTACGAGCGCTACGGCTGC
GACGCGGGCGGCGTGGTGTACCACGAGGCGGCCGACCACCGGCGCGCGTGCCAGCACGCGCCCTGCTGCTGCCC
GGACCGCGCGGGCGCGGCCGGCATCGGGGGCTGCGGCTTCGTCGGCTCCCGCCAGGACCTGCTCGACCACATCT
CCGGGCCCGACCACTCGCGCCCCATCATCGTCGTCCGCTACGGCCAGCCCTGGAACCTCAGCCTGCCGCTCTCGCG
CCGCTGGCACATCCTCGTCGGTGAGGAGGACAAGGCGGTGGCCGCCGCCGCGGGCGCCGACCGGCACCGCAACC
TCTTCCTCGTCTCCCTCGGCGAGCGCGGCGCCACCACGGCCGTGTCGCTGGTGTGCGTCCGGGCGGACGGCACGG
CGCCGGGCGCGCCGCAGTTCGCGTGCAAGCTCGCCGTGGAGAGCGACGGCTGCAGGCTGACCCTGGAGTCGCCG
CTGGTGTGCAGCAGCTCCCTGTCCGGCGGCCTGCCCGGCGAGGTCAAGTGCCTGCCGGTGCCCAAAGACTTTCTCT
CCGGCGACAGTGTGCCCCTCAGCATCCACATCGAGAAGCTCCCGGTTGCCCCCGCTCCTCCTCTAGGACCAGGAGT
GGCGCCGGCCTGTACCTCCCCGGTCGCCGCCACGCCTCCTCCTTGCCCGGCGGCCACCATCCCGCCTTCCCGTCCGT
CCTCCGCCGGCTCCTCCGACAA

>RAC875 TaSINA (4,816 4,818..Start codon) (4,816-4,989..exon 1) (5,097-5,936..exon 2) (4,700-4,815..5' UTR)

ATAATGAGTTATCACTCCCTATTCGGGTGAATCACAAAAGTTTAAATTAAGAACAATCCATTCCATCAAACTTTGAT
AATATTAGCTATTAATGTATAAAGATTAATACAATTATAGCTTTATTCGGCATGGATGCTATTCCATAGTACCATTAT
TCATAATATTATATAGAGTTAAACATACTGTAGATTATCCCAAATTGTAAACAACTTTAATAATAATACTCCCTCCAT
TTTTATATACAAGGCCACTATGGAATATACATTTTGCATATATATAAGGCCACCAACAGTAATCGAGGCAAAATTAA
TGATATTTTCTCGTACTAACAACCTGTTTAATACTTGCATGCTTGCAATCATAATGACAGTCAGCTACTTCCTCCACT
CAGTTTTTTTTGCATGCATGTGGAGTATTAATGATCCCAGTAAACAAGAAGAAAAGTTGACTTGTAAAGCAGGCAT
TAAATTTTACATTGATACCTGTAATCCAAGTTTGTGGCCTTATATACAAAAATGGAGGGAGTATAAAAATCTAGCA
GCGCAAATGCGCGGATAACTGTGCTAACTACTCAATAAGAGGTTAAAAGAAGATTCACATGCACAACTGTAGAAA
ACAAAAGGCCACACAACCAGTATTTAAAAAATGTACATTAATTGGACTCCCTTCATTCCTACTTACCATGCACATAT
TTTTTTCTAGTTTTTCGTGATTATCTAGGGTATTATGGTTCCCACACAGATGATTCTCTATCTTTCCAACCCTTATTTT
GGGTAGTACTACCAATGCATATATTATCAATGTTCTTTCCATAAAATGCTCCCGTGACATTAACTATCCCACCATGC
ATGGAGGAAATTAATGCATGCATATCATTAACTATCTCAACGTCTAAACCGAAACAGGGCACCGCCAAATGCTGAG
GGCAAGCTCGTCCAACTACACATCCAATTGTCAATGTCCACCACTCATTTATATGCGGGGCTTGAACTAAAAAATTA
GATACTCCCTCCGTCTAGGTGTGTAAGTCATCTTACGAAAACCAAATAATCCCAAAATAATTAGGCGTGGTGCATT
AACTTCTACCTCATTTCTTGTTTTTGACATATCAACCAATAATAGATGAGGGTGTGCATGCTTTTAATGACTTGAGAC
TATTAAACACGACATGCAGTGGTTAGTTCATTGCATGCAATACTATTAATTAGCAAATAAACATTAATGTCTCTCAT
TTTCCCCTCCTCCTTGGTCACGGTGCACAGCCTAAGATGACTTACTCACGTAGACGGAGGGAGTACTCACTTCAGA
TGGGAATACAAGTCGGGCACATTTTCTAGGTCTTCAATTTGACAAGCGAATAATATGCCACAAAAAAATGGTAAGA
AACCTCACCTGGCAAGGAATATACATTTTTTGTGGCATATAACATATATTTTGCTTGTCAAATAGATATCCTAAAAA
TCGGTGTCTGTGTATATTCCCACCCAAAGGGAGTAACAATTAGGAATTAAGAGAGCTTTTATTATGGTAGACCCTT
CTTTCATTAAGATACCCACGTCTAAGGACATCATCAATGTATCATTCTTAAGTCGGTAATTAGCTGGGGCCACACTA
TGCTATAGCAAACTCGAATTCCTCCTATGCTTAATTCCAATATGGCTATGGACGTTAGGGTGAAGCCCACTTGCCG
GTATTTTCTCTTGTAGTTTTAACCCCTCAAATCATGTGAGGGGAGAAAGAAATAGTTTTTGATTCCTTGTGGCATGG
CCTTAGCACTATGACCGGTCTTAAAGATTTTTGTTGCTAGGACCCCTTCCCACAATGCTTGAGGATATGTCATTAGA
CCCATATTTTTGCAACAATGGAGACATAATATAAGATTTGATGATATATAGGTTATATTTCATTAGAGATGATCTCT
TAGGCCTGTCTCGATGGATATTATCATAAACTATTATCATTAAATGATGCTACTTTGCTTACTACATTCCCATCATTT
CATAGCATCATAACATATATTTTACTGGGAATAAACTTATATTTTATAACTTAAAATTATTATCAAAATGTGACAAAA
TACAAGAGGACTAGTGAATCAGGACGGAGGCGGTAGGAAAAGAGACAGTGTCACGGTGGAGCATCGGGAGAGA
GAGTCCCAACAGTAAAAAGCCAAACTCACTGAAAAGTAGTGATTAAAAAGAAGATTCACATGCCCACTTGCAGTA
CTACCAAGCAAAAACACAAGGCCAATCGACCAGTAATGTATAATTAAAAACATTGTCACGGTTCCCCAGTTGAAAT
TTTTTGGGTCATGGTGTTCAACAGAGTTATTTAATGTTCTACTAGAGGGCATTAATGTGACATATCGTAGTTTAATC
GCGGTGGCGTGGCAATGACATTGGCAAGCAAACTAAACTAGCTGAGTGTCCTTGTCCTCTCCACCCACCACTAGTC
AAGCCTAATTAGCTAAGCTGCGTTGGAAAGGGCTAAAAGTGAGTCTCTCTGGACCTGGAATCCAAACATTCAAAA
ACGATTTTGGTGAGAGCATCTCCAGTCGCACCCTCAACACGGCCTTTCTGGCGATTTTTTCGTGCCGGCGCCTAAAA
ATGGCCCAGTCGCGTCCCCAGGAGCCCGTTTTTCACCGGCTTGGGCCGAAATCAGTACAGACGGACCCAGGCCGA
ACCCGGCGCACTGGGGGCGCCGGGGCGAGTGGTTTTGGCGCGAAATAGCCGCGGGCCCACCGAGTCTGCGAGA
CGGCCGCTTCGTCGCCCTCATCGCCTTGGTTCCCACGGGAATCAATGCCAAGACTGCCACGATGCCGCCGCCGGTC
AGCCTTGCCATTGATAGCTCATCACGGGCGGCGCGTCACGGGTGGCGCGGCGACGCCTCCCCTCCCGCCACGCGT
ACACACGGGCGCGACTATATAAGCCGGTGGCCTCCCTCACCTTTGGCCACACCAGCCCTCGCCGTCCATCGACCGC
CGTCGAGCTCTTCCTCTCCCGTGCGCAGCCGCGACGCTCCTTCCCTCCCTCTCCCTCACTCCCAAGCCCGGTGGCCG
CGCGTTTCCCCGGCGACGGCCAACGGCTTCGGTCACCGCTCGCTTCACGAGTGGGAGGCGTGGCTCCTGTTCGAG
GCGAACATCCCGGCGCCACCGGACATGCGCGCATGAACGATGGGGTGGAGACTCGGCAACGGGGGAGTGCCCA

TTCCCCCGGTGCCCGACGTCGAGGCGTGCCCCGCCTTCTTCGCCGCCGAGGTCGACCGCGTGCAAGCCTTCTCACA
GCGGAGCAACTCGCCCCCCACCAGTACGCCGTCGAAAACCACGCGGCGTGGGCGGCGTACTTCCAGCGCCGCCA
GGAGCAAAGACTGGCGTCCACCAACGGGGCGCCGATGGTGGCAGCGTCAAGAATAGTGAGGGGCACCGCGTGT
GGTGGGGCGCCCCCGAATATGCTCGACGGCGTGCTGTCGTACCTCGAGGGCGGTAACGAACCGTCGCTGGCATA
CCGTCCCATGGCGGCCGCCCCGGTCCACCGCCGGCACGCCGGGCAATGGATGCCCAGGAGGTTCGGTTCCTCCTC
GACTTCCTCCTCGCACTCCTCCTCCCGTTCTTCTTCCCACTCCTCCGGCTCGACGACGTTGCTCGGCGTCAAGGCCGA
GCCCGCAGCGGAGACGCTGCTCGGCCGGCGCACTCGCAGCGTCGGCATCATCATAAACGAGGGCGGCCGGCGCT
CCTCCTCATCGGCTCCTCCGCGCTTCGTCAAACCAAAGACGGGAACCGAGGCTCGCGGCCGTGAAAACGGAGCCG
GGGCTCCCCGCCGTGAAGACAGAGCACGGCGACGTGGAGCTCGATGAGGAGGCGACCCTAAAATGGGCGCGCA
CCGACTGGCTCAAGATGGAGTGGGAGCGCCAGTGCGCCGCCCTGCAGCACTACGAACAGCGCCGCCAGGGCCGC
GACGAAGGAGGCGTCGTCGTCCTCAACGACAACGACGACGACGACGCGCCGCCACCGCCACCGGTCCGCCATGG
CGACGCCGGGCAAGGGTCCAGCAGGGGCGGCCGCGTCAAGGAGGAGAAGACCGCCGCCGCCGACGACGCGCCG
CCACCTCCACCGGTCCGCCATGGCGACGCCGGACAAGGGTCCAGCAGGGGCGGCCGCGTAAAGGAGGAGAAGG
CCGCCGCCGACGACTACGCCGCGTTCAGCGACTTCTTCGCACCTTCGTTGTAGTTTCGGCTTTTTTAGATAAATTTG
CTATATAAACTGTCTTCTTTGTGTAATATATGTCCAGTTTACCGAATTTAGCCGAACTTTATTGAATGTTTTTTTTTAA
TTTAGACGGCGTCTGGGAATGGTCATGGGATCGGCGACTGAGAATCCAGTTGCCCCCAGGCGGCGATTTTACGCG
CCCCTTGGGGAACGGCTGGAGATGCTCTGAGTCTCCTTGATGCCCGAGCTCTCGGATGAATGGCAGATTTCTTTTG
AATGTTGCAGTTGGGTGAATGCGGTTAGGTTAGGTGGATGGTAAAAAGTAAAAACAACTTCTATTCCCAAAGTCT
GAACCCGTCAAAATCTGGCTATGGGCACGGCCCCATCTCATCAGAGACCCCACCAGACCTCCTGTGACGGGGCTC
GCACCCGATACCATCTTGATCGGATCCATACCCCTTAACCCCCTTGACCTCACCTTCGCTCACAAGCGCCAGTACAC
AGAGCAGAGAGCCGCCCGCCTGAGTGCTACCCGCCGCTGCTGGTGCCAACAGACAGCCGCCCGCCCGCCCGCCCG
CCTGACTGAATCTTGGTGGGGGAGGAGAGGAGAGATGGGCCTCGGCGGCGGCGGCGGAGAGGGGATCCTGGCC
GTGGCCGTGGTGCCCATGGAGGCCATCCCCATGGCGGAGCCGCAGATCCTCGCCGGCGGCAAGACCGTCGGCCT
GCGGGCGGACCTGCTCGACTGCCACAACTGCCGCCTCCCCCTCAAGCCCCCCATATTCAAGGTGAGATCCGCCGGC
CGCCCCTCTTCTCCAAGATTCATTTTTCCGTTTTCTTGAATCTGGCACGGCGTGACCGAGCGTATCTGGTGTGCATG
AATCTTGGCCGGCAGTGCGACGCCGAGCACCTGGTCTGCTCCTCCTGCCGCGGCGCCCACGCCGAGGCCTGCGGC
GGCCGCGCCGCCGTCCACTCCGCGCTCGCCGACATCTTCGCGGCCGCCGCCACCGTGCCCTGCGGCTACGAGCGC
TACGGCTGCGACGCCGGCGGCGTGGTGTACCACGAGGCCGCCGACCACCGGCGCGCGTGCCAGCACGCGCCCTG
CTGCTGCCCGGACCGCGCGGGCGCGGCCGGCATCGGGGGCTGCGGCTTCGTCGGCTCCCGCCAGGACCTGCTCG
ACCACATCTCCGGCCCCGACCACTCGCGCCCCATCATCGTCGTCCGCTACGGCCAGCCGTGGAACCTCAGCCTGCC
GCTCTCGCGCCGCTGGCACATCCTCGTCGGCGAGGAGGACAAGGCGGTGGCCGCCGCCGCGGGCGCCGACCGGC
ACCGCAACCTCTTCCTCGTCTCCCTCGGCGAGCGCGGCGCCACCACGGCCGTGTCGCTGGTGTGCGTCCGGGCGG
ACGGCACGGCGCCGGGCGCGCCGCAGTTCGCGTGCAAGCTCGCCGTGGAGAGCGACGGCTGCAGGCTGACCCTG
GAGTCGCCTCTGGTGTGCAGCAGCTCCCTGTCCGGCGGCCTGCCGGGCGAGGTCAAGTGCCTGCCGGTGCCCAAA
GACTTCCTGTCCGGCGACAGTGTGCCCCTCAGCATCCACATCGAGAAGCTCCCGGCTCCCCCAGCTCCTCCTCTAG
GACCAGGAGTGGCGCCGGCCTGTACTTCCCCGGTCGCCGCCACCCCTCCTCCCTGCCCGGCCGCCACCATCCCGCC
TTCTCGTCCGTCCTCCGCCGGCTCCTCCGAC

>Drysdale TaSINA (4,814-4,816..Start codon) (4,814-4,987..exon 1) (5,095-5,935..exon 2) (4,698-4,813..5' UTR)

ATAATGAGTTATCACTCCCTATTCGGGTGAATCACAAAAGTTTAAATTAAGAACAATCCATTCCATCAAACTTTGAT
AATATTAGCTATTAATGTATAAAGATTAATACAATTATAGCTTTATTCGGCATGGATGCTATTCCATAGTACCATTAT
TCATAATATTATATAGAGTTAAACATACTGTAGATTATCCCAAATTGTAAACAACTTTAATAATAATACTCCCTCCAT

TTTTATATACAAGGCCACTATGGAATATACATTTTGCATATATATAAGGCCACCAACAGTAATCGAGGCAAAATTAA
TGATATTTTCTCGTACTAACAACCTGTTTAATACTTGCATGCTTGCAATCATAATGACAGTCAGCTACTTCCTCCACT
CAGTTTTTTTTGCATGCATGTGGAGTATTAATGATCCCAGTAAACAAGAAGAAAAGTTGACTTGTAAAGCAGGCAT
TAAATTTTACATTGATACCTGTAATCCAAGTTTGTGGCCTTATATACAAAAATGGAGGGAGTATAAAAATCTAGCA
GCGCAAATGCGCGGATAACTGTGCTAACTACTCAATAAGAGGTTAAAAGAAGATTCACATGCACAACTGTAGAAA
ACAAAAGGCCACACAACCAGTATTTAAAAAATGTACATTAATTGGACTCCCTTCATTCCTACTTACCATGCACATAT
TTTTTTCTAGTTTTTCGTGATTATCTAGGGTATTATGGTTCCCACACAGATGATTCTCTATCTTTCCAACCCTTATTTT
GGGTAGTACTACCAATGCATATATTATCAATGTTCTTTCCATAAAATGCTCCCGTGACATTAACTATCCCACCATGC
ATGGGAGGAATTAATGCATGCATATCATTAACTATCTCAACGTCTAAACCGAAACAGGCACCGCCAGATGCTGAG
GGCAAGCTCGTCCAACTACACATCCAATTGTCAATGTCCACCACTCATTTATATGCGGGCTTGAACTAAAAAATTAG
ATACTCCCTCCGTCTAGGTGTGTAAGTCATCTTACGAAAACCAAATAATCCCAAAATAATTAGGCGTGGTGCATTAA
CTTCTACCTCATTTCTTGTTTTTGACATATCAACCAATAATAGATGAGGGTGTGCATGCTTTTAATGACTTGAGACTA
TTAAACACGACATGCAGTGGTTAGTTCATTGCATGCAATACTATTAATTAGCAAATAAACATTAATGTCTCTCATTTT
CCCCTCCTCCTTGGTCACGGTGCACAGCCTAAGATGACTTACTCACGTAGACGGAGGGAGTACTCACTTCAGATGG
GAATACAAGTCGGGCACATTTTCTAGGTCTTCAATTTGACAAGCGAATAATATGCCACAAAAAAATGGTAAGAAAC
CTCACCTGGCAAGGAATATACATTTTTTGTGGCATATAACATATATTTTGCTTGTCAAATAGATATCCTAAAAATCG
GTGTCTGTGTATATTCCCACCCAAAGGGAGTAACAATTAGGAATTAAGAGAGCTTTTATTATGGTAGACCCTTCTTT
CATTAAGATACCCACGTCTAAGGACATCATCAATGTATCATTCTTAAGTCGGTAATTAGCTGGGGCCACACTATGCT
ATAGCAAACTCGAATTCCTCCTATGCTTAATTCCAATATGGCTATGGACGTTAGGGTGAAGCCCACTTGCCGGTATT
TTCTCTTGTAGTTTTAACCCCTCAAATCATGTGAGGGGAGAAAGAAATAGTTTTTGATTCCTTGTGGCATGGCCTTA
GCACTATGACCGGTCTTAAAGATTTTTGTTGCTAGGACCCCTTCCCACAATGCTTGAGGATATGTCATTAGACCCAT
ATTTTTGCAACAATGGAGACATAATATAAGATTTGATGATATATAGGTTATATTTCATTAGAGATGATCTCTTAGGC
CTGTCTCGATGGATATTATCATAAACTATTATCATTAAATGATGCTACTTTGCTTACTACATTCCCATCATTTCATAGC
ATCATAACATATATTTTACTGGGAATAAACTTATATTTTATAACTTAAAATTATTATCAAAATGTGACAAAATACAAG
AGGACTAGTGAATCAGGACGGAGGCGGTAGGAAAAGAGACAGTGTCACGGTGGAGCATCGGGAGAGAGAGTCC
CAACAGTAAAAAGCCAAACTCACTGAAAAGTAGTGATTAAAAAGAAGATTCACATGCCCACTTGCAGTACTACCAA
GCAAAAACACAAGGCCAATCGACCAGTAATGTATAATTAAAAACATTGTCACGGTTCCCCAGTTGAAATTTTTTGG
GTCATGGTGTTCAACAGAGTTATTTAATGTTCTACTAGAGGGCATTAATGTGACATATCGTAGTTTAATCGCGGTG
GCGTGGCAATGACATTGGCAAGCAAACTAAACTAGCTGAGTGTCCTTGTCCTCTCCACCCACCACTAGTCAAGCCT
AATTAGCTAAGCTGCGTTGGAAAGGGCTAAAAGTGAGTCTCTCTGGACCTGGAATCCAAACATTCAAAAACGATTT
GGTGAGAGCATCTCCAGTCGCACCCTCAACACGGCCTTTCTGGCGATTTTTTCGTGCCGGCGCCTAAAAATGGCCC
AGTCGCGTCCCCAGGAGCCCGTTTTTCACCGGCTTGGGCCGAAATCAGTACAGACGGACCCAGGCCGAACCCGGC
GCACTGGGGGCGCCGGGGCGAGTGGTTTTGGCGCGAAATAGCCGCGGGCCCACCGAGTCTGCGAGACGGCCGC
TTCGTCGCCCTCATCGCCTTGGTTCCCACGGGAATCAATGCCAAGACTGCCACGATGCCGCCGCCGGTCAGCCTTG
CCATTGATAGCTCATCACGGGCGGCGCGTCACGGGTGGCGCGGCGACGCCTCCCCTCCCGCCACGCGTACACACG
GGCGCGACTATATAAGCCGGTGGCCTCCCTCACCTTTGGCCACACCAGCCCTCGCCGTCCATCGACCGCCGTCGAG
CTCTTCCTCTCCCGTGCGCAGCCGCGACGCTCCTTCCCTCCCTCTCCCTCACTCCCAAGCCCGGTGGCCGCGCGTTTC
CCCGGCGACGGCCAACGGCTTCGGTCACCGCTCGCTTCACGAGTGGGAGGCGTGGCTCCTGTTCGAGGCGAACAT
CCCGGCGCCACCGGACATGCGCGCATGAACGATGGGGTGGAGACTCGGCAACGGGGGAGTGCCCATTCCCCCGG
TGCCCGACGTCGAGGCGTGCCCCGCCTTCTTCGCCGCCGAGGTCGACCGCGTGCAAGCCTTCTCACAGCGGAGCA
ACTCGCCCCCCACCAGTACGCCGTCGAAAACCACGCGGCGTGGGCGGCGTACTTCCAGCGCCGCCAGGAGCAAAG
ACTGGCGTCCACCAACGGGGCGCCGATGGTGGGCAGCGTCAAGAATAGTGAGGGGCACCGCGTGTGGTGGGGC
GCCCCCGAATATGCTCGACGGCGTGCTGTCGTACCTCGAGGGCGGTAACGAACCGTCGCTGGCATACCGTCCCAT
GGCGGCCGCCCCGGTCCACCGCCGGCACGCCGGGCAATGGATGCCCAGGAGGTTCGGTTCCTCCTCGACTTCCTC

CTCGCACTCCTCCTCCCGTTCTTCTTCCCACTCCTCCGGCTCGACGACGTTGCTCGGCGTCAAGGCCGAGCCCGCAG
CGGAGACGCTGCTCGGCCGGCGCACTCGCAGCGTCGGCATCATCATAAACGAGGGCGGCCGGCGCTCCTCCTCAT
CGGCTCCTCCGCGCTTCGTCAAACCAAAGACGGGAACCGAGGCTCGCGGCCGTGAAAACGGAGCCGGGGCTCCC
CGCCGTGAAGACAGAGCACGGCGACGTGGAGCTCGATGAGGAGGCGACCCTAAAATGGGCGCGCACCGACTGG
CTCAAGATGGAGTGGGAGCGCCAGTGCGCCGCCCTGCAGCACTACGAACAGCGCCGCCAGGGCCGCGACGAAGG
AGGCGTCGTCGTCCTCAACGACAACGACGACGACGACGCGCCGCCACCGCCACCGGTCCGCCATGGCGACGCCG
GGCAAGGGTCCAGCAGGGGCGGCCGCGTCAAGGAGGAGAAGACCGCCGCCGCCGACGACGCGCCGCCACCTCC
ACCGGTCCGCCATGGCGACGCCGGACAAGGGTCCAGCAGGGGCGGCCGCGTAAAGGAGGAGAAGGCCGCCGCC
GACGACTACGCCGCGTTCAGCGACTTCTTCGCACCTTCGTTGTAGTTTCGGCTTTTTTAGATAAATTTGCTATATAAA
CTGTCTTCTTTGTGTAATATATGTCCAGTTTACCGAATTTAGCCGAACTTTATTGAATGTTTTTTTTTAATTTAGACGG
CGTCTGGGAATGGTCATGGGATCGGCGACTGAGAATCCAGTTGCCCCCAGGCGGCGATTTTACGCTCCCCTTGGG
GAACGGCTGGAGATGCTCTGAGTCTCCTTGATGCCCGAGCTCTCGGATGAATGGCAGATTTCTTTTGAATGTTGCA
GTTGGGTGAATGCGGTTAGGTTAGGTGGATGGTAAAAAGTAAAAACAACTTCTATTCCCAAAGTCTGAACCCGTC
AAAATCTGGCTATGGGCACGGCCCCATCTCATCAGAGACCCCACCAGACGTCCTGTGACGGGGCTCGCACCCGAT
ACCATTTTGATCGGATCCATACCCCTTAACCCCCTTGACCTCACCTTCGCTCACAAGCGCCAGTACACAGAGCAGAG
AGCCGCCCGCCTGAGTGCTACCCGCCGCTGCTGGTGCCAACAGACAGCCGCCCGCCCGCCCGCCCGCCTGACTGA
ATCTTGGTGGGGGAGGAGAGGAGAGATGGGCCTCGGCGGCGGCGGCGGAGAGGGGATCCTGGCCGTGGCCGT
GGTGCCCATGGAGGCCATCCCCATGGCGGAGCCGCAGATCCTCGCCGGCGGCAAGACCGTCGGCCTGCGGGCGG
ACCTGCTCGACTGCCACAACTGCCGCCTCCCCCTCAAGCCCCCCATATTCAAGGTGAGATCCGCCGGCCGCCCCTCT
TCTCCAAGATTCATTTTTCCGTTTTCTTGAATCTGGCACGGCGTGACCGAGCGTATCTGGTGTGCATGAATCTTGGC
CGGCAGTGCGACGCCGAGCACCTGGTCTGCTCCTCCTGCCGCGGCGCCCACGCCGAGGCCTGCGGCGGCCGCGCC
GCCGTCCACTCCGCGCTCGCCGACATCTTCGCGGCCGCCGCCACCGTGCCCTGCGGCTACGAGCGCTACGGCTGC
GACGCCGGCGGCGTGGTGTACCACGAGGCCGCCGACCACCGGCGCGCGTGCCAGCACGCGCCCTGCTGCTGCCC
GGACCGCGCGGGCGCGGCCGGCATCGGGGGCTGCGGCTTCGTCGGCTCCCGCCAGGACCTGCTCGACCACATCT
CCGGCCCCGACCACTCGCGCCCCATCATCGTCGTCCGCTACGGCCAGCCGTGGAACCTCAGCCTGCCGCTCTCGCG
CCGCTGGCACATCCTCGTCGGCGAGGAGGACAAGGCGGTGGCCGCCGCCGCGGGCGCCGACCGGCACCGCAACC
TCTTCCTCGTCTCCCTCGGCGAGCGCGGCGCCACCACGGCCGTGTCGCTGGTGTGCGTCCGGGCGGACGGCACGG
CGCCGGGCGCGCCGCAGTTCGCGTGCAAGCTCGCCGTGGAGAGCGACGGCTGCAGGCTGACCCTGGAGTCGCCT
CTGGTGTGCAGCAGCTCCCTGTCCGGCGGCCTGCCGGGCGAGGTCAAGTGCCTGCCGGTGCCCAAAGACTTCCTG
TCCGGCGACAGTGTGCCCCTCAGCATCCACATCGAGAAGCTCCCGGCTCCCCCAGCTCCTCCTCTAGGACCAGGAG
TGGCGCCGGCCTGTACTTCCCCGGTCGCCGCCACCCCTCCTCCCTGCCCGGCCGCCACCATCCCGCCTTCTCGTCCG
TCCTCCGCCGGCTCCTCCGACA

>Gladius TaSINA (4,809-4,811..Start codon) (4,809-4,982..exon 1) (5,090-5,920..exon 2) (4,693-4,808..5' UTR)

ATAATGAGTTATCACTCCCTATTTGGGTGAATCACAAAAGTTTAAATTAAGAACAATCCATTCCATCAAACTTTGAT
AATATTAGCTATTAATGTATAAAGATTAATACAATTATAGCTTTATTCGGCATGGATGCTATTCCATAGTACCATTAT
TCATAATATTATATTGAGTTAAACATACTGTAGATTATCCCAAATTGTAAACAACTTTAATAATAATACTCCCTCCAT
TTTTATATACAAGGCCACTATGGAATATACATTTTGCATATATATAAGGCCACCAACAGTAATCGAGGCAAATTTAA
TGATATTTTCTCGTACTAATAACCTGTTTAATACTTGCATGCTTGCAATCATAATGACAGTCAGCTACTTCCTCCACT
CAGTTTTTTTGCATGCATGTGGAGTATTAATGATCCCAGTAAACAAGAAGAAAAGTTGACTTGTAAAGCAGGCATT
AAATTTTACATTGGTACCTGTAATCCAAGTTTGTGGCCTTATATACAAAAATGGAGGGAGTATAAAAATCTAGCAG
CGCAAATGCGCGGATAACTGTGCTAACTACTCAATAAGAGGTTAAAAGAAGATTCACATGCACAACTGTAGAAAA
CAAAAGGCCACACAACCAGTATTTAAAAAATGTACATTAATTGGACTCCCTTCATTCCTACTTACCATGCACATATTT

TTTTCTAGTTTTTCGTGATTATCTAGGGTATTATGGTTCCCACACAGATGATTCTCTATCTTTCCAACCCTTATTTTGG
GTAGTACTACCAATGCATATATTATCAATGTTCTTCCCATAAAATGCTCCCGTGACATTAACTATCCCACCATGCATG
GAGGAAATTAATGCATGCATATCATTAACTATCTCAACGTCTAAACCGAAACAAGGCACCGCCAGATGCCGAGGG
CAAGCTCGTCCAACTACACATCCAATTGTCAATCTCCACCACTCATTTATATGCGGGGCTTGAACTAAAAAAATGAT
ACTCCCTCCGTCTAGGTGTGTAAGTCATCTTACGAAAACCAAATAATCCCAAAATAATTAGGCGTGGTGCATTAACT
TCTACCTCGTTTCTGTTTTTTGACATATCAACCAATAATAGATGAGGGTGTGCATGCTTTTAATGACTTGAGACTATT
AAACACGACATGCAGTGGTTAGTTCATTGCATGCAATACTATTAATTAGCAAATAAACATTAATGTCTCTCATTTTC
CCCTCCTCCTTGGTCACGGTGCACAGCCTAAGATGACTTACTCACCTAGACGGAGGGAGTACTCACTTCAGATGGG
AATACAAGTCGGGCACAGTTTCTAGGTCTTCAATTTGACAAGCGAATAATATGCCACAAAAAAATGGTAAGAAACC
TCACCTGGCAAGGAATATACATTTTTTGTGGCATATAACATATATTTTGCTTGTCAAATAGATAACCTAAAAATCGG
TGTCTGTGTATATTCCCACCCAAAGGGAGTAACAATTAGGAATTAAGAGAGCTTTTATTATGGTAGACCCTTCTTTC
ATTAAGATGCCCACGTCTAAGGACATCATCAATGTATCATTCTTAAGTCGGTAATTAGCTGGGGCCACACTATGCT
ATAGCAAACTCGAATTCCTCCTATGCTTAATTCCAATATGGCTATGGACGTTAGGGTGAAGCCCACTTGCCGGTATT
TTCTCTTGTAGTTTTAACCCCTCAAATCATGTGAGGGGAGAAAGAAATAGTTTTTGATTCCTTGTGGCATGGCCTTA
GCACTATGACCGGTCTTAAAGATTTTTGTTGCTAGGACCCCTTCCCACAATGCTTGAGGATATGTCATTAGACCCAT
ATTTTTGCAACAATGGAGACATAATATAAGATTTGATGATATATAGGTTATATTTCATTAGAGATGATCTCTTAGGC
CTGTCTCGATGGATATTATCATAAACTATTATCATTAAATGATGCTACTTTGCTTACTACATTCCCATCATTTCATAGC
ATCATAACATATATTTTACTGGGAATAAACTTATATTTTATAACTTAAAATTATTATCAAAATGTGACAAAATACAAG
AGGACTAGTGAATCAGGACGGAGGCGGTAGGAAAAGAGACAGTGTCACGGTGGAGCATCGGGAGAGAGAGTCC
CAACAGTAAAAAGCCAAACTCACTGAAAAGTAATGATTAAAAAGAAGATTCACATGCCCACTTGCAGTACTACCAA
GCAAAAACACAAGGCCAATCGACCAGTAATGTATAATTAAAAACATTGTCACGGTTCCCCAGTTGAAATTTTTTGG
GTCATGGTGTTCAACAGAGTTATTTAATGTTCTACTAGAGGGCATTAATGTGACATATCGTAGTTTAATCGCGGTG
GCGTGGCAATGACATTGGCAAGCAAACTAAACTAGCTGAGTGTCCTTGTCCTCTCCACCCACCACTAGTCAAGCCT
AATTAGCTAAGCTGCGTTGGAGAGGGCTAAAAGTGAGTCTCTCTGGACCTGGAATCCAAACATTCAAAAACGATTT
GGTGAGAGCATCTCCAGTCGCATCCCCAACACGGCCTTCCAGGCGATTTTTTCGTGCCGGCGCCTAAAAATGGCCC
AGTCGCGTCCCCAGGAGCCCGTTTTTCACCGGCTTGGGCCGAAATCAATACAGGCGGACCCAGGCCGAACCCGGC
GCGCTGGGGGCGCCGGGGCGAGTGGTTTTGGCGCGAAATAGCCCCGGGCCCACCGAGTCTGCGAGACGGCCGCT
TCGTCGCCCTCATCGCCTTGGTTCCCACGGGAATCAATGCCAAGACTGCCACGATGCCGTCGCCGGTCAGCCTTGC
CATTGATAGCTCATCACGGGCGGCGCGTCACGGGTGGCGCGGCGACGCCTCCCCTCCCGCCACGCGTACACACGG
GCGCGACTATATAAGCCGGTGGCCTCCCTCACCTTTGGCCACACCAGCCCTCGCCGTCCATCGACCGCCGTCGAGC
TCTTCCTCTCCCGTGCGCAGCCGCGACGCTTCTTCCCTCCCTCTCCCTCACTCCCAAGCCCGGTGGCCGCGCGTTTCC
CCGGCGACGGCCAACGGCTTCGGTCACCGCTCGCTTCACGAGTGGGAGGCGTGGCTCCTGTTCGAGGCGAACATC
CCGGCGCCACCGGACATGCGCGCATGAACGATGGGGTGGAGACTCGGCAACGGGGGAGTGCCCATTCCCCCGGT
GCCCGACGTCGAGGCGTGCCCCGCCTTCTTCGCCGCCGAGGTCGACCGCGTGCAAGCCTCCCTCACAGCGGAGCA
ACTCGCCCCCCACCAGTACGCCGTCGAAAACCACGCGGCGTGGGCGGCGTACTTCCAGCACCGCCAGGAGCAAAG
ACTGGCGTCCACCAACGGGGCGCCGATGGTGGGCAGCGTCAAGAACAGTGAGGGGCACCGCGTGTGGTGGGGC
GCCACCGAATATGCTCGACGGCGTGCTGTCGTACCTCGAGGGCGGTAACGAACCGTCGCTGGCATACCGTCCCAT
GGCGGCCGCCCCGGTCCACCGCCGGCACGCGGGGCAATGGATGCCCAGGAGGTTCCGTTCCTCCTCGACTTCCTC
CTCGCACTCCTCCTCCCGTTCTTCTTCCCACTCCTCCGGCTCGACGACGTTGCTCGGCGTCAAGGCCGAGCCCGCAG
CGGAGACGCTGCTCAGCCGGCGCACTCGCAGCGTCGGCATCATCATAAACGAGGGCGGCCGGCGCTCCTCCTCAT
CGGCTCCTCCGCGCTTCGTCAAACCAAAGACGGAACCGAGGCTCGCGGCCGTGAAAACGGAGCCGGGGCTCCCC
GCCGTGAAGACAGAGCACGGCGACGTGGAGCTCGATGAGGAGGCGACCCTAAAATGGGCGCGCGCCGACTGGC
TCAAGATGGAGTGGGAGCGCCAGTGCGCCGCCCTGCAGCACTACGAACAGCGCCGCCAGGGCCGCGACGAAGG
AGGCGTCGTCATCCTCAACGACAACGACGACGACGACGCGCCGCCACCGCCACCGGTCCGCCATGGCGACGCCGG

GCAAGGGTCCAGCGGGGGCGGCCGCGTCAAGGAGGAGAAGACCGCCGCCGACGACGACGCGCCGCTCCACCGG
TCCGCCATGGCGACGCCGGGCAAGGGTCCAGCAGGGGCGGCCGCGTAAAGGAGGAGAAGGCCGCCGCCGACGA
CTACGCCGCGTTCAGCGACTTCTTCGCACCTTCGTTGTAGTTTCGGCTTTTTTGAGATAAATTTGCTATGTAAACTGT
CTTCTTTGTGTAATATATGTCCAGTTTACCGAATTTAGCCGAACTTTATTGAATGTTTTTTTTAATTTAGACGGCGTC
TGGGAACGGTCATGGGATCGGCGACTGGGAATCCAGTTGCCTTAGGCGGCGATTTTACGCGCCCCTTGGGGAAC
GGCTGGAGATGGTCTGAGTCTCCTTGATGCCCGAGCTCTCGGATGAATGGCAGANNNNNNNNNNNNNNNNNN
NNNNNNNNNNNNNNNNNNNNNNNNNNNNNNNNNNNNNNNNNNNNNNNNNNNNNNNNNNNNNNNNN
NNNNNNNNNNNNNNNNNNNNNNNNNNNNNNNNNNNNNNNNNNNNNNNNNNNNNNNNNNNNNNNNN
NNNNNNNNNNNNNNNNNNNNNNNNNNNNNNNNNNNNNNNNNNNNNNNNNNNNNNNNNNNNNNNNN
NNNNNNNNNNNNNNNNNNNNNNNNNNNNNNNNNNNNNNNNNNNNNNNNNNNNNNNNNNNNNNNNN
NNNNNNNNNNNNNNNNNNNNNNNNNNNNNNNNNNNNNNNNNNNNNNNNNNNNNNNNNNNNNNNNN
NNNNNNNNNNNNNNNNNNNNNNNNNNNNNNNNNNNNNNNNNNNNNNNNNNNNNNNNNNNNNNNNN
NNNNNNNNNNNNNNNNNNNNNNNNNNNNNNNNNNNNNNNNCCGTCGGCCTGCGGGCGGACCTGCTCGAC
TGCCACAACTGCCGCCTCCCCCTCAAGCCCCCCATATTCAAGGTGAGATCCGCCGGCCGCCCCTCTTCCCCAAGATT
CATTTTTCCGTTTTCTTGAATCTGGCACGGCGTGACCGAGCGTATCTGGTGTGCATGAATCTTGGCCGGCAGTGCG
ACGCCGAGCACCTGGTCTGCTCCTCCTGCCGCGGCGCCCACGCCGAGGCCTGCGGCGGCCGCGCCGCCGTCCACT
CCGCGCTCGCCGACATCTTCGCGGCCGCCGCCACCGTGCCCTGCGGCTACGAGCGCTACGGCTGCGACGCGGGCG
GCGTGGTGTACCACGAGGCGGCCGACCACCGGCGCGCGTGCCAGCACGCGCCCTGCTGCTGCCCGGACCGCGCG
GGCGCGGCCGGCATCGGGGGCTGCGGCTTCGTCGGCTCCCGCCAGGACCTGCTCGACCACATCTCCGGGCCCGAC
CACTCGCGCCCCATCATCGTCGTCCGCTACGGCCAGCCCTGGAACCTCAGCCTGCCGCTCTCGCGCCGCTGGCACA
TCCTCGTCGGTGAGGAGGACAAGGCGGTGGCCGCCGCCGCGGGCGCCGACCGGCACCGCAACCTCTTCCTCGTCT
CCCTCGGCGAGCGCGGCGCCACCACGGCCGTGTCGCTGGTGTGCGTCCGGGCGGACGGCACGGCGCCGGGCGC
GCCGCAGTTCGCGTGCAAGCTCGCCGTGGAGAGCGACGGCTGCAGGCTGACCCGGAGTCGCCGCTGGTGTGCAG
CAGCTCCCTGTCCGGCGGCCTGCCCGGCGAGGTCAAGTGCCTGCCGGTGCCCAAAGACTTTCTCTCCGGCGACAG
TGTGCCCCTCAGCATCCACATCGAGAAGCTCCCGGTTGCCCCCGCTCCTCCTCTAGGACCAGGAGTGGCGCCGGCC
TGTACCTCCCCGGTCGCCGCCACGCCTCCTCCTTGCCCGGCGGCCACCATCCCGCCTTCCCGTCCGTCCTCCGCCGG
CT

Supplementary Fig. S5 Wheat *TaSINA* gene sequences retrieved from 10+Genome dataset https://wheat.pw.usda.gov/blastn) using Chinese Spring TraesCS3B01G572900 gene as query. The fasta sequences include exons and introns.

>**Mace** TraesMAC3B01G633400.1 chr3B:822719144..822720339 (- strand) class=mRNA length=1196

ATGGGCCTCGGCGGCGGCGGAGAGGGGATCCTGGCCGTGGCCGTGGTGCCCATGGAGGCCATCCCCATGG
CGGAGCCGCAGATCCTCGCCGGCGGCAAGACCGTCGGCCTGCGGGCGGACCTGCTCGACTGCCACAACTGCCGC
CTCCCCCTCAAGCCCCCATATTCAAGGTGAGATCCGCCGGCCGCCCCTCTTCCCCAAGATTCATTTTTCCGTTTTCT
TGAATCTGGCACGGCGTGACCGAGCGTATCTGGTGTGCATGAATCTTGGCCGGCAGTGCGACGCCGAGCACCTGG
TCTGCTCCTCCTGCCGCGGCGCCCACGCCGAGGCCTGCGGCGGCCGCGCCGCCGTCCACTCCGCGCTCGCCGACAT
CTTCGCGGCCGCCGCCACCGTGCCCTGCGGCTACGAGCGCTACGGCTGCGACGCGGGCGGCGTGGTGTACCACG
AGGCGGCCGACCACCGGCGCGCGTGCCAGCACGCGCCCTGCTGCTGCCCGGACCGCGCGGGCGCGGCCGGCATC
GGGGGCTGCGGCTTCGTCGGCTCCCGCCAGGACCTGCTCGACCACATCTCCGGGCCCGACCACTCGCGCCCCATC
ATCGTCGTCCGCTACGGCCAGCCCTGGAACCTCAGCCTGCCGCTCTCGCGCCGCTGGCACATCCTCGTCGGTGAGG
AGGACAAGGCGGTGGCCGCCGCCGCGGGCGCCGACCGGCACCGCAACCTCTTCCTCGTCTCCCTCGGCGAGCGC
GGCGCCACCACGGCCGTGTCGCTGGTGTGCGTCCGGGCGGACGGCACGGCGCCGGGCGCGCCGCAGTTCGCGTG
CAAGCTCGCCGTGGAGAGCGACGGCTGCAGGCTGACCCTGGAGTCGCCGCTGGTGTGCAGCAGCTCCCTGTCCG
GCGGCCTGCCCGGCGAGGTCAAGTGCCTGCCGGTGCCCAAAGACTTTCTCTCCGGCGACAGTGTGCCCCTCAGCA
TCCACATCGAGAAGCTCCCGGTTGCCCCCGCTCCTCCTCTAGGACCAGGAGTGGCGCCGGCCTGTACCTCCCCGGT
CGCCGCCACGCCTCCTCCTTGCCCGGCGGCCACCATCCCGCCTTCCCGTCCGTCCTCCGCCGGCTCCTCCGACAACG
TCGCAGTCAAGACGGTGATCACCGATCAGAGCTACAAGAAACGGAAGTCTGCCAACCCAAGGAAGCTGTAG

ESPLVCSSSLSGGLPGEVKCLPVPKDFLSGDSVPLSI**HIEKL**PVAP**APPL**GPGVAPACTSPVAATPPPCPAATIPPSRPSSA
GSSDNVAVKTVITDQSYKKRKSANPRKL

Blastn score: 2695.52 (2988), E value: 0, Identity: 1494/1494 (100%), Gaps: 0/1494 (0%), Strand: + / -

> **Longreach Lancer** TraesLAC3B01G635900.1 chr3B:812952783..812953978 (- strand) class=mRNA length=1196

ATGGGCCTCGGCGGCGGCGGAGAGGGGATCCTGGCCGTGGCCGTGGTGCCCATGGAGGCCATCCCCATGG
CGGAGCCGCAGATCCTCGCCGGCGGCAAGACCGTCGGCCTGCGGGCGGACCTGCTCGACTGCCACAACTGCCGC
CTCCCCCTCAAGCCCCCATATTCAAGGTGAGATCCGCCGGCCGCCCCTCTTCTCCAAGATTCATTTTTCCGTTTTCT
TGAATCTGGCACGGCGTGACCGAGCGTATCTGGTGTGCATGAATCTTGGCCGGCAGTGCGACGCCGAGCACCTGG
TCTGCTCCTCCTGCCGCGGCGCCCACGCCGAGGCCTGCGGCGGCCGCGCCGCCGTCCACTCCGCGCTCGCCGACAT
CTTCGCGGCCGCCGCCACCGTGCCCTGCGGCTACGAGCGCTACGGCTGCGACGCGGGCGGCGTGGTGTACCACG
AGGCGGCCGACCACCGGCGCGCGTGCCAGCACGCGCCCTGCTGCTGCCCGGACCGCGCGGGCGCGGCCGGCATC
GGGGGCTGCGGCTTCGTCGGCTCCCGCCAGGACCTGCTCGACCACATCTCCGGGCCCGACCACTCGCGCCCCATC
ATCGTCGTCCGCTACGGCCAGCCCTGGAACCTCAGCCTGCCGCTCTCGCGCCGCTGGCACATCCTCGTCGGCGAGG
AGGACAAGGCGGTGGCCGCCGCCGCGGGCGCCGACCGGCACCGCAACCTCTTCCTCGTCTCCCTCGGCGAGCGC
GGCGCCACCACGGCCGTGTCGCTGGTGTGCGTCCGGGCGGACGGCACGGCGCCGGGCGCGCCGCAGTTCGCGTG
CAAGCTCGCCGTGGAGAGCGACGGCTGCAGGCTGACCCTGGAGTCGCCGCTGGTGTGCAGCAGCTCCCTGTCCG
GCGGCCTGCCCGGCGAGGTCAAGTGCCTGCCGGTGCCCAAAGACTTTCTCTCCGGCGACAGTGTGCCCCTCAGCA
TCCACATCGAGAAGCTCCCGGTTGCCCCCGCTCCTCCTCTAGGACCAGGAGTGGCGCCGGCCTGTACCTCCCCGGT

CGCCGCCACGCCTCCTCCTTGCCCGGCGGCCACCATCCCGCCTTCCCGTCCGTCCTCCGCCGGCTCCTCCGACAACG
TCGCAGTCAAGACGGTGATCACCGATCAGAGCTACAAGAAACGGAAGTCTGCCAACCCAAGGAAGCTGTAG

ESPLVCSSSLSGGLPGEVKCLPVPKDFLSGDSVPLS**IHIEKLPVAPAPP**LGPGVAPACTSPVAATPPPCPAATIPPSRPSSA
GSSDNVAVKTVITDQSYKKRKSANPRKL

Blastn score: 2674.78 (2965), E value: 0, Identity: 1492/1498 (99.6%), Gaps: 4/1498 (0.3%), Strand: + / -

> **CDC Stanley** chr3B TraesSTA3B01G630200.1 chr3B:829890824..829892019 (- strand) class=mRNA length=1196

ATGGGCCTCGGCGGCGGCGGCGGAGAGGGGATCCTGGCCGTGGCCGTGGTGCCCATGGAGGCCATCCCCATGG
CGGAGCCGCAGATCCTCGCCGGCGGCAAGACCGTCGGCCTGCGGGCGGACCTGCTCGACTGCCACAACTGCCGC
CTCCCCCTCAAGCCCCCCATATTCAAGGTGAGATCCGCCGGCCGCCCCTCTTCCCCAAGATTCATTTTTCCGTTTTCT
TGAATCTGGCACGGCGTGACCGAGCGTATCTGGTGTGCATGAATCTTGGCCGGCAGTGCGACGCCGAGCACCTGG
TCTGCTCCTCCTGCCGCGGCGCCCACGCCGAGGCCTGCGGCGGCCGCGCCGCCGTCCACTCCGCGCTCGCCGACAT
CTTCGCGGCCGCCGCCACCGTGCCCTGCGGCTACGAGCGCTACGGCTGCGACGCGGGCGGCGTGGTGTACCACG
AGGCGGCCGACCACCGGCGCGCGTGCCAGCACGCGCCCTGCTGCTGCCCGGACCGCGCGGGCGCGGCCGGCATC
GGGGGCTGCGGCTTCGTCGGCTCCCGCCAGGACCTGCTCGACCACATCTCCGGGCCCGACCACTCGCGCCCCATC
ATCGTCGTCCGCTACGGCCAGCCCTGGAACCTCAGCCTGCCGCTCTCGCGCCGCTGGCACATCCTCGTCGGTGAGG
AGGACAAGGCGGTGGCCGCCGCCGCGGGCGCCGACCGGCACCGCAACCTCTTCCTCGTCTCCCTCGGCGAGCGC
GGCGCCACCACGGCCGTGTCGCTGGTGTGCGTCCGGGCGGACGGCACGGCGCCGGGCGCGCCGCAGTTCGCGTG
CAAGCTCGCCGTGGAGAGCGACGGCTGCAGGCTGACCCTGGAGTCGCCGCTGGTGTGCAGCAGCTCCCTGTCCG
GCGGCCTGCCCGGCGAGGTCAAGTGCCTGCCGGTGCCCAAAGACTTTCTCTCCGGCGACAGTGTGCCCCTCAGCA
TCCACATCGAGAAGCTCCCGGTTGCCCCCGCTCCTCCTCTAGGACCAGGAGTGGCGCCGGCCTGTACCTCCCCGGT
CGCCGCCACGCCTCCTCCTTGCCCGGCGGCCACCATCCCGCCTTCCCGTCCGTCCTCCGCCGGCTCCTCCGACAACG
TCGCAGTCAAGACGGTGATCACCGATCAGAGCTACAAGAAACGGAAGTCTGCCAACCCAAGGAAGCTGTAG

ESPLVCSSSLSGGLPGEVKCLPVPKDFLSGDSVPLSI**HIEKLPVAPAPPL**GPGVAPACTSPVAATPPPCPAATIPPSRPSSA
GSSDNVAVKTVITDQSYKKRKSANPRKLX

> CDC Landmark chr3B chr3B:821846270..821847465 (+ strand) class=mRNA length=1196

ATGGGCCTCGGCGGCGGCGGCGGAGAGGGGATCCTGGCCGTGGCCGTGGTGCCCATGGAGGCCATCCCCATGG
CGGAGCCGCAGATCCTCGCCGGCGGCAAGACCGTCGGCCTGCGGGCGGACCTGCTCGACTGCCACAACTGCCGC
CTCCCCCTCAAGCCCCCCATATTCAAGGTGAGATCCGCCGGCCGCCCCTCTTCCCCAAGATTCATTTTTCCGTTTTCT
TGAATCTGGCACGGCGTGACCGAGCGTATCTGGTGTGCATGAATCTTGGCCGGCAGTGCGACGCCGAGCACCTGG
TCTGCTCCTCCTGCCGCGGCGCCCACGCCGAGGCCTGCGGCGGCCGCGCCGCCGTCCACTCCGCGCTCGCCGACAT
CTTCGCGGCCGCCGCCACCGTGCCCTGCGGCTACGAGCGCTACGGCTGCGACGCGGGCGGCGTGGTGTACCACG
AGGCGGCCGACCACCGGCGCGCGTGCCAGCACGCGCCCTGCTGCTGCCCGGACCGCGCGGGCGCGGCCGGCATC
GGGGGCTGCGGCTTCGTCGGCTCCCGCCAGGACCTGCTCGACCACATCTCCGGGCCCGACCACTCGCGCCCCATC
ATCGTCGTCCGCTACGGCCAGCCCTGGAACCTCAGCCTGCCGCTCTCGCGCCGCTGGCACATCCTCGTCGGTGAGG
AGGACAAGGCGGTGGCCGCCGCCGCGGGCGCCGACCGGCACCGCAACCTCTTCCTCGTCTCCCTCGGCGAGCGC
GGCGCCACCACGGCCGTGTCGCTGGTGTGCGTCCGGGCGGACGGCACGGCGCCGGGCGCGCCGCAGTTCGCGTG
CAAGCTCGCCGTGGAGAGCGACGGCTGCAGGCTGACCCTGGAGTCGCCGCTGGTGTGCAGCAGCTCCCTGTCCG
GCGGCCTGCCCGGCGAGGTCAAGTGCCTGCCGGTGCCCAAAGACTTTCTCTCCGGCGACAGTGTGCCCCTCAGCA
TCCACATCGAGAAGCTCCCGGTTGCCCCCGCTCCTCCTCTAGGACCAGGAGTGGCGCCGGCCTGTACCTCCCCGGT

CGCCGCCACGCCTCCTCCTTGCCCGGCGGCCACCATCCCGCCTTCCCGTCCGTCCTCCGCCGGCTCCTCCGACAACG
TCGCAGTCAAGACGGTGATCACCGATCAGAGCTACAAGAAACGGAAGTCTGCCAACCCAAGGAAGCTGTAG

ESPLVCSSSLSGGLPGEVKCLPVPKDFLSGDSVPLSI**HIEKL**PVAPA**PP**LGPGVAPACTSPVAATPPPCPAATIPPSRPSSA
GSSDNVAVKTVITDQSYKKRKSANPRKLX

Blastn score: 2695.52 (2988), E value: 0, Identity: 1494/1494 (100%), Gaps: 0/1494 (0%), Strand: + / +

> PI190962 *Triticum aestivum ssp. spelta* chr3B chr3B:807307688..807308883 (- strand) class=mRNA length=1196

ATGGGCCTCGGCGGCGGCGGCGGAGAGGGGATCCTGGCCGTGGCCGTGGTGCCCATGGAGGCCATCCCCATGG
CGGAGCCGCAGATCCTCGCCGGCGGCAAGACCGTCGGCCTGCGGGCGGACCTGCTCGACTGCCACAACTGCCGC
CTCCCCCTCAAGCCCCCCATATTCAAGGTGAGATCCGCCGGCCGCCCCTCTTCTCCAAGATTCATTTTTCCGTTTTCT
TGAATCTGGCACGGCGTGACCGAGCGTATCTGGTGTGCATGAATCTTGGCCGGCAGTGCGACGCCGAGCACCTGG
TCTGCTCCTCCTGCCGCGGCGCCCACGCCGAGGCCTGCGGCGGCCGCGCCGCCGTCCACTCCGCGCTCGCCGACAT
CTTCGCGGCCGCCGCCACCGTGCCCTGCGGCTACGAGCGCTACGGCTGCGACGCCGGCGGCGTGGTGTACCACGA
GGCCGCCGACCACCGGCGCGCGTGCCAGCACGCGCCCTGCTGCTGCCCGGACCGCGCGGGCGCGGCCGGCATCG
GGGGCTGCGGCTTCGTCGGCTCCCGCCAGGACCTGCTCGACCACATCTCCGGCCCCGACCACTCGCGCCCCATCAT
CGTCGTCCGCTACGGCCAGCCGTGGAACCTCAGCCTGCCGCTCTCGCGCCGCTGGCACATCCTCGTCGGCGAGGA
GGACAAGGCGGTGGCCGCCGCCGCGGGCGCCGACCGGCACCGCAACCTCTTCCTCGTCTCCCTCGGCGAGCGCG
GCGCCACCACGGCCGTGTCGCTGGTGTGCGTCCGGGCGGACGGCACGGCGCCGGGCGCGCCGCAGTTCGCGTGC
AAGCTCGCCGTGGAGAGCGACGGCTGCAGGCTGACCCTGGAGTCGCCTCTGGTGTGCAGCAGCTCCCTGTCCGGC
GGCCTGCCGGGCGAGGTCAAGTGCCTGCCGGTGCCCAAAGACTTCCTGTCCGGCGACAGTGTGCCCCTCAGCATC
CACATCGAGAAGCTCCCGGCTCCCCCAGCTCCTCCTCTAGGACCAGGAGTGGCGCCGGCCTGTACTTCCCCGGTCG
CCGCCACCCCTCCTCCCTGCCCGGCCGCCACCATCCCGCCTTCTCGTCCGTCCTCCGCCGGCTCCTCCGACAACGTC
GCCGTTAAGACGGTGATCACCGATCAGAGCTACAAGAAACGGAAGTCCGCCAACCCAAGGAAGCTGTAG

ESPLVCSSSLSGGLPGEVKCLPVPKDFLSGDSVPLSI**HIEKL**PAPPA**PPL**GPGVAPACTSPVAATPPPCPAATIPPSRPSSA
GSSDNVAVKTVITDQSYKKRKSANPRKLX

Blastn score: 2538.63 (2814), E value: 0, Identity: 1460/1493 (97.8%), Gaps: 3/1493 (0.2%), Strand: + / -

>**Norin 61** chr3B chr3B:823641574..823642769 (+ strand) class=mRNA length=1196

ATGGGCCTCGGCGGCGGCGGCGGAGAGGGGATCCTGGCCGTGGCCGTGGTGCCCATGGAGGCCATCCCCATGG
CGGAGCCGCAGATCCTCGCCGGCGGCAAGACCGTCGGCCTGCGGGCGGACCTGCTCGACTGCCACAACTGCCGC
CTCCCCCTCAAGCCCCCCATATTCAAGGTGAGATCCGCCGGCCGCCCCTCTTCTCCAAGATTCATTTTTCCGTTTTCT
TGAATCTGGCACGGCGTGACCGAGCGTATCTGGTGTGCATGAATCTTGGCCGGCAGTGCGACGCCGAGCACCTGG
TCTGCTCCTCCTGCCGCGGCGCCCACGCCGAGGCCTGCGGCGGCCGCGCCGCCGTCCACTCCGCGCTCGCCGACAT
CTTCGCGGCCGCCGCCACCGTGCCCTGCGGCTACGAGCGCTACGGCTGCGACGCCGGCGGCGTGGTGTACCACGA
GGCCGCCGACCACCGGCGCGCGTGCCAGCACGCGCCCTGCTGCTGCCCGGACCGCGCGGGCGCGGCCGGCATCG
GGGGCTGCGGCTTCGTCGGCTCCCGCCAGGACCTGCTCGACCACATCTCCGGCCCCGACCACTCGCGCCCCATCAT
CGTCGTCCGCTACGGCCAGCCGTGGAACCTCAGCCTGCCGCTCTCGCGCCGCTGGCACATCCTCGTCGGCGAGGA
GGACAAGGCGGTGGCCGCCGCCGCGGGCGCCGACCGGCACCGCAACCTCTTCCTCGTCTCCCTCGGCGAGCGCG
GCGCCACCACGGCCGTGTCGCTGGTGTGCGTCCGGGCGGACGGCACGGCGCCGGGCGCGCCGCAGTTCGCGTGC

AAGCTCGCCGTGGAGAGCGACGGCTGCAGGCTGACCCTGGAGTCGCCTCTGGTGTGCAGCAGCTCCCTGTCCGGC
GGCCTGCCGGGCGAGGTCAAGTGCCTGCCGGTGCCCAAAGACTTCCTGTCCGGCGACAGTGTGCCCCTCAGCATC
CACATCGAGAAGCTCCCGGCTCCCCCAGCTCCTCCTCTAGGACCAGGAGTGGCGCCGGCCTGTACTTCCCCGGTCG
CCGCCACCCCTCCTCCCTGCCCGGCCGCCACCATCCCGCCTTCTCGTCCGTCCTCCGCCGGCTCCTCCGACAACGTC
GCCGTTAAGACGGTGATCACCGATCAGAGCTACAAGAAACGGAAGTCCGCCAACCCAAGGAAGCTGTAG

ESPLVCSSSLSGGLPGEVKCLPVPKDFLSGDSVPLSI**HIEKL**PAPPA**PPL**GPGVAPACTSPVAATPPPCPAATIPPSRPSSA
GSSDNVAVKTVITDQSYKKRKSANPRKLX

Blastn score: 2538.63 (2814), E value: 0, Identity: 1460/1493 (97.8%), Gaps: 3/1493 (0.2%), Strand: + / +

>SY Mattis TraesSYM3B01G629300.1 chr3B:808881627..808882822 (- strand) class=mRNA length=1196
ATGGGCCTCGGCGGCGGCGGCGGAGAGGGGATCCTGGCCGTGGCCGTGGTGCCCATGGAGGCCATCCCCATGG
CGGAGCCGCAGATCCTCGCCGGCGGCAAGACCGTCGGCCTGCGGGCGGACCTGCTCGACTGCCACAACTGCCGC
CTCCCCCTCAAGCCCCCCATATTCAAGGTGAGATCCGCCGGCCGCCCCTCTTCTCCAAGATTCATTTTTCCGTTTTCT
TGAATCTGGCACGGCGTGACCGAGCGTATCTGGTGTGCATGAATCTTGGCCGGCAGTGCGACGCCGAGCACCTGG
TCTGCTCCTCCTGCCGCGGCGCCCACGCCGAGGCCTGCGGCGGCCGCGCCGCCGTCCACTCCGCGCTCGCCGACAT
CTTCGCGGCCGCCGCCACCGTGCCCTGCGGCTACGAGCGCTACGGCTGCGACGCCGGCGGCGTGGTGTACCACGA
GGCCGCCGACCACCGGCGCGCGTGCCAGCACGCGCCCTGCTGCTGCCCGGACCGCGCGGGCGCGGCCGGCATCG
GGGGCTGCGGCTTCGTCGGCTCCCGCCAGGACCTGCTCGACCACATCTCCGGCCCCGACCACTCGCGCCCCATCAT
CGTCGTCCGCTACGGCCAGCCGTGGAACCTCAGCCTGCCGCTCTCGCGCCGCTGGCACATCCTCGTCGGCGAGGA
GGACAAGGCGGTGGCCGCCGCCGCGGGCGCCGACCGGCACCGCAACCTCTTCCTCGTCTCCCTCGGCGAGCGCG
GCGCCACCACGGCCGTGTCGCTGGTGTGCGTCCGGGCGGACGGCACGGCGCCGGGCGCGCCGCAGTTCGCGTGC
AAGCTCGCCGTGGAGAGCGACGGCTGCAGGCTGACCCTGGAGTCGCCTCTGGTGTGCAGCAGCTCCCTGTCCGGC
GGCCTGCCGGGCGAGGTCAAGTGCCTGCCGGTGCCCAAAGACTTCCTGTCCGGCGACAGTGTGCCCCTCAGCATC
CACATCGAGAAGCTCCCGGCTCCCCCAGCTCCTCCTCTAGGACCAGGAGTGGCGCCGGCCTGTACTTCCCCGGTCG
CCGCCACCCCTCCTCCCTGCCCGGCCGCCACCATCCCGCCTTCTCGTCCGTCCTCCGCCGGCTCCTCCGACAACGTC
GCCGTTAAGACGGTGATCACCGATCAGAGCTACAAGAAACGGAAGTCCGCCAACCCAAGGAAGCTGTAG

ESPLVCSSSLSGGLPGEVKCLPVPKDFLSGDSVPLSI**HIEKL**PAPPA**PPL**GPGVAPACTSPVAATPPPCPAATIPPSRPSSA
GSSDNVAVKTVITDQSYKKRKSANPRKLX

Blastn score: 2538.63 (2814), E value: 0, Identity: 1460/1493 (97.8%), Gaps: 3/1493 (0.2%), Strand: + / -

>**Julius** TraesJUL3B01G636200.1 chr3B chr3B:830706300..830707495 (- strand) class=mRNA
length=1196

ATGGGCCTCGGCGGCGGCGGCGGAGAGGGGATCCTGGCCGTGGCCGTGGTGCCCATGGAGGCCATCCCCATGG
CGGAGCCGCAGATCCTCGCCGGCGGCAAGACCGTCGGCCTGCGGGCGGACCTGCTCGACTGCCACAACTGCCGC
CTCCCCCTCAAGCCCCCCATATTCAAGGTGAGATCCGCCGGCCGCCCCTCTTCTCCAAGATTCATTTTTCcGTTTTCTT
GAATCTGGCACGGCGTGACCGAGCGTATCTGGTGTGCATGAATCTTGGCCGGCAGTGCGACGCCGAGCACCTGGT
CTGCTCCTCCTGCCGCGGCGCCCACGCCGAGGCCTGCGGCGGCCGCGCCGCCGTCCACTCCGCGCTCGCCGACAT
CTTCGCGGCCGCCGCCACCGTGCCCTGCGGCTACGAGCGCTACGGCTGCGACGCCGGCGGCGTGGTGTACCACGA
GGCCGCCGACCACCGGCGCGCGTGCCAGCACGCGCCCTGCTGCTGCCCGGACCGCGCGGGCGCGGCCGGCATCG
GGGGCTGCGGCTTCGTCGGCTCCCGCCAGGACCTGCTCGACCACATCTCCGGCCCCGACCACTCGCGCCCCATCAT
CGTCGTCCGCTACGGCCAGCCGTGGAACCTCAGCCTGCCGCTCTCGCGCCGCTGGCACATCCTCGTCGGCGAGGA

GGACAAGGCGGTGGCCGCCGCCGCGGGCGCCGACCGGCACCGCAACCTCTTCCTCGTCTCCCTCGGCGAGCGCG
GCGCCACCACGGCCGTGTCGCTGGTGTGCGTCCGGGCGGACGGCACGGCGCCGGGCGCGCCGCAGTTCGCGTGC
AAGCTCGCCGTGGAGAGCGACGGCTGCAGGCTGACCCTGGAGTCGCCTCTGGTGTGCAGCAGCTCCCTGTCCGGC
GGCCTGCCGGGCGAGGTCAAGTGCCTGCCGGTGCCCAAAGACTTCCTGTCCGGCGACAGTGTGCCCCTCAGCATC
CACATCGAGAAGCTCCCGGCTCCCCCAGCTCCTCCTCTAGGACCAGGAGTGGCGCCGGCCTGTACTTCCCCGGTCG
CCGCCACCCCTCCTCCCTGCCCGGCCGCCACCATCCCGCCTTCTCGTCCGTCCTCCGCCGGCTCCTCCGACAACGTC
GCCGTTAAGACGGTGATCACCGATCAGAGCTACAAGAAACGGAAGTCCGCCAACCCAAGGAAGCTGTAG

ESPLVCSSSLSGGLPGEVKCLPVPKDFLSGDSVPLSI**HIEKL**PAPPA**PPL**GPGVAPACTSPVAATPPPCPAATIPPSRPSSA
GSSDNVAVKTVITDQSYKKRKSANPRKLX

Blastn score: 2538.63 (2814), E value: 0, Identity: 1460/1493 (97.8%), Gaps: 3/1493 (0.2%), Strand: + / -

>**Jagger** TraesJAG3B01G629200.1 chr3B chr3B:827852942..827854137 (+ strand) class=mRNA length=1196

ATGGGCCTCGGCGGCGGCGGCGGAGAGGGGATCCTGGCCGTGGCCGTGGTGCCCATGGAGGCCATCCCCATGG
CGGAGCCGCAGATCCTCGCCGGCGGCAAGACCgTCGGCCTGCGGGCGGACCTGCTCGACTGCCACAACTGCCGCC
TCCCCCTCAAGCCCCCCATATTCAAGGTGAGATCCGCCGGCCGCCCCTCTTCTCCAAGATTCATTTTTCCGTTTTCTT
GAATCTGGCACGGCGTGACCGAGCGTATCTGGTGTGCATGAATCTTGGCCGGCAGTGCGACGCCGAGCACCTGGT
CTGCTCCTCCTGCCGCGGCGCCCACGCCGAGGCCTGCGGCGGCCGCGCCGCCGTCCACTCCGCGCTCGCCGACAT
CTTCGCGGCCGCCGCCACCGTGCCCTGCGGCTACGAGCGCTACGGCTGCGACGCCGGCGGCGTGGTGTACCACGA
GGCCGCCGACCACCGGCGCGCGTGCCAGCACGCGCCCTGCTGCTGCCCGGACCGCGCGGGCGCGGCCGGCATCG
GGGGCTGCGGCTTCGTCGGCTCCCGCCAGGACCTGCTCGACCACATCTCCGGCCCCGACCACTCGCGCCCCATCAT
CGTCGTCCGCTACGGCCAGCCGTGGAACCTCAGCCTGCCGCTCTCGCGCCGCTGGCACATCCTCGTCGGCGAGGA
GGACAAGGCGGTGGCCGCCGCCGCGGGCGCCGACCGGCACCGCAACCTCTTCCTCGTCTCCCTCGGCGAGCGCG
GCGCCACCACGGCCGTGTCGCTGGTGTGCGTCCGGGCGGACGGCACGGCGCCGGGCGCGCCGCAGTTCGCGTGC
AAGCTCGCCGTGGAGAGCGACGGCTGCAGGCTGACCCTGGAGTCGCCTCTGGTGTGCAGCAGCTCCCTGTCCGGC
GGCCTGCCGGGCGAGGTCAAGTGCCTGCCGGTGCCCAAAGACTTCCTGTCCGGCGACAGTGTGCCCCTCAGCATC
CACATCGAGAAGCTCCCGGCTCCCCCAGCTCCTCCTCTAGGACCAGGAGTGGCGCCGGCCTGTACTTCCCCGGTCG
CCGCCACCCCTCCTCCCTGCCCGGCCGCCACCATCCCGCCTTCTCGTCCGTCCTCCGCCGGCTCCTCCGACAACGTC
GCCGTTAAGACGGTGATCACCGATCAGAGCTACAAGAAACGGAAGTCCGCCAACCCAAGGAAGCTGTAG

LESPLVCSSSLSGGLPGEVKCLPVPKDFLSGDSVPLSI**HIEKL**PAPPA**PPL**GPGVAPACTSPVAATPPPCPAATIPPSRPSSA
GSSDNVAVKTVITDQSYKKRKSANPRKL

Blastn score: 2538.63 (2814), E value: 0, Identity: 1460/1493 (97.8%), Gaps: 3/1493 (0.2%), Strand: + / +

>**Arina** TraesARI3B01G636300.1 chr3B chr3B:861871096..861872291 (- strand) class=mRNA length=1196

ATGGGCCTCGGCGGCGGCGGCGGAGAGGGGATCCTGGCCGTGGCCGTGGTGCCCATGGAGGCCATCCCCATGG
CGGAGCCGCAGATCCTCGCCGGCGGCAAGACCGTCGGCCTGCGGGCGGACCTGCTCGACTGCCACAACTGCCGC
CTCCCCCTCAAGCCCCCCATATTCAAGGTGAGATCCGCCGGCCGCCCCTCTTCTCCAAGATTCATTTTTCCGTTTTCT
TGAATCTGGCACGGCGTGACCGAGCGTATCTGGTGTGCATGAATCTTGGCCGGCAGTGCGACGCCGAGCACCTGG
TCTGCTCCTCCTGCCGCGGCGCCCACGCCGAGGCCTGCGGCGGCCGCGCCGCCGTCCACTCCGCGCTCGCCGACAT

CTTCGCGGCCGCCGCCACCGTGCCCTGCGGCTACGAGCGCTACGGCTGCGACGCCGGCGGCGTGGTGTACCACGA
GGCCGCCGACCACCGGCGCGCGTGCCAGCACGCGCCCTGCTGCTGCCCGGACCGCGCGGGCGCGGCCGGCATCG
GGGGCTGCGGCTTCGTCGGCTCCCGCCAGGACCTGCTCGACCACATCTCCGGCCCCGACCACTCGCGCCCCATCAT
CGTCGTCCGCTACGGCCAGCCGTGGAACCTCAGCCTGCCGCTCTCGCGCCGCTGGCACATCCTCGTCGGCGAGGA
GGACAAGGCGGTGGCCGCCGCCGCGGGCGCCGACCGGCACCGCAACCTCTTCCTCGTCTCCCTCGGCGAGCGCG
GCGCCACCACGGCCGTGTCGCTGGTGTGCGTCCGGGCGGACGGCACGGCGCCGGGCGCGCCGCAGTTCGCGTGC
AAGCTCGCCGTGGAGAGCGACGGCTGCAGGCTGACCCTGGAGTCGCCTCTGGTGTGCAGCAGCTCCCTGTCCGGC
GGCCTGCCGGGCGAGGTCAAGTGCCTGCCGGTGCCCAAAGACTTCCTGTCCGGCGACAGTGTGCCCCTCAGCATC
CACATCGAGAAGCTCCCGGCTCCCCCAGCTCCTCCTCTAGGACCAGGAGTGGCGCCGGCCTGTACTTCCCCGGTCG
CCGCCACCCCTCCTCCCTGCCCGGCCGCCACCATCCCGCCTTCTCGTCCGTCCTCCGCCGGCTCCTCCGACAACGTC
GCCGTTAAGACGGTGATCACCGATCAGAGCTACAAGAAACGGAAGTCCGCCAACCCAAGGAAGCTGTAG

ESPLVCSSSLSGGLPGEVKCLPVPKDFLSGDSVPLSI**HIEKL**PAPPA**PPL**GPGVAPACTSPVAATPPPCPAATIPPSRPSSA
GSSDNVAVKTVITDQSYKKRKSANPRKL

Blastn score: 2538.63 (2814), E value: 0, Identity: 1460/1493 (97.8%), Gaps: 3/1493 (0.2%), Strand: + / -
