# Supplementary material for: The wheat Seven in absentia gene is associated with increases in biomass and yield in hot climates
Source: J Exp Bot. 2021 Feb 5;72(10):3774–91. doi: 10.1093/jxb/erab044 (PMC8096608; doi:10.1093/jxb/erab044)
Supplement: erab044_suppl_Supplementary-Figure-S12 [file erab044_suppl_supplementary-figure-s12.pdf]

Supplementary Fig. S12 CLUSTAL multiple sequence alignment of *TaSINA* gene sequences of 14 wheat varieties by MUSCLE (v3.8, EMBL-EBI). Purple: rare variants. v: variants between RAC875 and Kukri haplotypes. Yellow: promoter domain specific of RAC875 haplotype. Blue: promoter domain specific of Kukri haplotype. Green: position of start and stop of CDS.

```
                                          v
Drysdale        ---ATAATGAGTTATCACTCCCTATTCGGGTGAATCACAAAAGTTTAAATTAAGAACAAT
RAC875          ---ATAATGAGTTATCACTCCCTATTCGGGTGAATCACAAAAGTTTAAATTAAGAACAAT
Norin61         ---ATAATGAGTTATCACTCCCTATTCGGGTGAATCACAAAAGTTTAAATTAAGAACAAT
Jagger          -ACATAATGAGTTATCACTCCCTATTCGGGTGAATCACAAAAGTTTAAATTAAGAACAAT
Mattis          -ACATAATGAGTTATCACTCCCTATTCGGGTGAATCACAAAAGTTTAAATTAAGAACAAT
Julius          --CATAATGAGTTATCACTCCCTATTCGGGTGAATCACAAAAGTTTAAATTAAGAACAAT
Arina           ---ATAATGAGTTATCACTCCCTATTCGGGTGAATCACAAAAGTTTAAATTAAGAACAAT
Spelta          ---ATAATGAGTTATCACTCCCTATTCGGGTGAATCACAAAAGTTTAAATTAAGAACAAT
Lancer          ---ATAATGAGTTATCACTCCCTATTCGGGTGAATCACAAAAGTTTAAATTAAGAACAAT
Gladius         ---ATAATGAGTTATCACTCCCTATTTGGGTGAATCACAAAAGTTTAAATTAAGAACAAT
Kukri           ---ATAATGAGTTATCACTCCCTATTTGGGTGAATCACAAAAGTTTAAATTAAGAACAAT
Landmark        AACATAATGAGTTATCACTCCCTATTTGGGTGAATCACAAAAGTTTAAATTAAGAACAAT
Mace            ---ATAATGAGTTATCACTCCCTATTTGGGTGAATCACAAAAGTTTAAATTAAGAACAAT
Stanley         ---ATAATGAGTTATCACTCCCTATTTGGGTGAATCACAAAAGTTTAAATTAAGAACAAT
                   *********************** *********************************

Drysdale        CCATTCCATCAAACTTTGATAATATTAGCTATTAATGTATAAAGATTAATACAATTATAG
RAC875          CCATTCCATCAAACTTTGATAATATTAGCTATTAATGTATAAAGATTAATACAATTATAG
Norin61         CCATTCCATCAAACTTTGATAATATTAGCTATTAATGTATAAAGATTAATACAATTATAG
Jagger          CCATTCCATCAAACTTTGATAATATTAGCTATTAATGTATAAAGATTAATACAATTATAG
Mattis          CCATTCCATCAAACTTTGATAATATTAGCTATTAATGTATAAAGATTAATACAATTATAG
Julius          CCATTCCATCAAACTTTGATAATATTAGCTATTAATGTATAAAGATTAATACAATTATAG
Arina           CCATTCCATCAAACTTTGATAATATTAGCTATTAATGTATAAAGATTAATACAATTATAG
Spelta          CCATTCCATCAAACTTTGATAATATTAGCTATTAATGTATAAAGATTAATACAATTATAG
Lancer          CCATTCCATCAAACTTTGATAATATTAGCTATTAATGTATAAAGATTAATACAATTATAG
Gladius         CCATTCCATCAAACTTTGATAATATTAGCTATTAATGTATAAAGATTAATACAATTATAG
Kukri           CCATTCCATCAAACTTTGATAATATTAGCTATTAATGTATAAAGATTAATACAATTATAG
Landmark        CCATTCCATCAAACTTTGATAATATTAGCTATTAATGTATAAAGATTAATACAATTATAG
Mace            CCATTCCATCAAACTTTGATAATATTAGCTATTAATGTATAAAGATTAATACAATTATAG
STANLEY         CCATTCCATCAAACTTTGATAATATTAGCTATTAATGTATAAAGATTAATACAATTATAG
                ************************************************************

                                                                    v
Drysdale        CTTTATTCGGCATGGATGCTATTCCATAGTACCATTATTCATAATATTATATAGAGTTAA
RAC875          CTTTATTCGGCATGGATGCTATTCCATAGTACCATTATTCATAATATTATATAGAGTTAA
Norin61         CTTTATTCGGCATGGATGCTATTCCATAGTACCATTATTCATAATATTATATAGAGTTAA
Jagger          CTTTATTCGGCATGGATGCTATTCCATAGTACCATTATTCATAATATTATATAGAGTTAA
Mattis          CTTTATTCGGCATGGATGCTATTCCATAGTACCATTATTCATAATATTATATAGAGTTAA
Julius          CTTTATTCGGCATGGATGCTATTCCATAGTACCATTATTCATAATATTATATAGAGTTAA
Arina           CTTTATTCGGCATGGATGCTATTCCATAGTACCATTATTCATAATATTATATAGAGTTAA
Spelta          CTTTATTCGGCATGGATGCTATTCCATAGTACCATTATTCATAATATTATATAGAGTTAA
Lancer          CTTTATTCGGCATGGATGCTATTCCATAGTACCATTATTCATAATATTATATAGAGTTAA
Gladius         CTTTATTCGGCATGGATGCTATTCCATAGTACCATTATTCATAATATTATATTGAGTTAA
Kukri           CTTTATTCGGCATGGATGCTATTCCATAGTACCATTATTCATAATATTATATTGAGTTAA
Landmark        CTTTATTCGGCATGGATGCTATTCCATAGTACCATTATTCATAATATTATATTGAGTTAA
Mace            CTTTATTCGGCATGGATGCTATTCCATAGTACCATTATTCATAATATTATATTGAGTTAA
STANLEY         CTTTATTCGGCATGGATGCTATTCCATAGTACCATTATTCATAATATTATATTGAGTTAA
                **************************************************** *******

Drysdale        ACATACTGTAGATTATCCCAAATTGTAAACAACTTTAATAATAATACTCCCTCCATTTTT
RAC875          ACATACTGTAGATTATCCCAAATTGTAAACAACTTTAATAATAATACTCCCTCCATTTTT
Norin61         ACATACTGTAGATTATCCCAAATTGTAAACAACTTTAATAATAATACTCCCTCCATTTTT
Jagger          ACATACTGTAGATTATCCCAAATTGTAAACAACTTTAATAATAATACTCCCTCCATTTTT
Mattis          ACATACTGTAGATTATCCCAAATTGTAAACAACTTTAATAATAATACTCCCTCCATTTTT
Julius          ACATACTGTAGATTATCCCAAATTGTAAACAACTTTAATAATAATACTCCCTCCATTTTT
Arina           ACATACTGTAGATTATCCCAAATTGTAAACAACTTTAATAATAATACTCCCTCCATTTTT
Spelta          ACATACTGTAGATTATCCCAAATTGTAAACAACTTTAATAATAATACTCCCTCCATTTTT
Lancer          ACATACTGTAGATTATCCC-AATTGTAAACAACTTTAATAATAATACTCCCTCCATTTTT
Gladius         ACATACTGTAGATTATCCCAAATTGTAAACAACTTTAATAATAATACTCCCTCCATTTTT
Kukri           ACATACTGTAGATTATCCCAAATTGTAAACAACTTTAATAATAATACTCCCTCCATTTTT
Landmark        ACATACTGTAGATTATCCCAAATTGTAAACAACTTTAATAATAATACTCCCTCCATTTTT
Mace            ACATACTGTAGATTATCCCAAATTGTAAACAACTTTAATAATAATACTCCCTCCATTTTT
STANLEY         ACATACTGTAGATTATCCCAAATTGTAAACAACTTTAATAATAATACTCCCTCCATTTTT
                ******************* ****************************************
```

```
Drysdale        ATATACAAGGCCACTATGGAATATACATTTTGCATATATATAAGGCCACCAACAGTAATC
RAC875          ATATACAAGGCCACTATGGAATATACATTTTGCATATATATAAGGCCACCAACAGTAATC
Norin61         ATATACAAGGCCACTATGGAATATACATTTTGCATATATATAAGGCCACCAACAGTAATC
Jagger          ATATACAAGGCCACTATGGAATATACATTTTGCATATATATAAGGCCACCAACAGTAATC
Mattis          ATATACAAGGCCACTATGGAATATACATTTTGCATATATATAAGGCCACCAACAGTAATC
Julius          ATATACAAGGCCACTATGGAATATACATTTTGCATATATATAAGGCCACCAACAGTAATC
Arina           ATATACAAGGCCACTATGGAATATACATTTTGCATATATATAAGGCCACCAACAGTAATC
Spelta          ATATACAAGGCCACTATGGAATATACATTTTGCATATATATAAGGCCACCAACAGTAATC
Lancer          ATATACAAGGCCACTATGGAATATACATTTTGCATATATATAAGGCCACCAACAGTAATC
Gladius         ATATACAAGGCCACTATGGAATATACATTTTGCATATATATAAGGCCACCAACAGTAATC
Kukri           ATATACAAGGCCACTATGGAATATACATTTTGCATATATATAAGGCCACCAACAGTAATC
Landmark        ATATACAAGGCCACTATGGAATATACATTTTGCATATATATAAGGCCACCAACAGTAATC
Mace            ATATACAAGGCCACTATGGAATATACATTTTGCATATATATAAGGCCACCAACAGTAATC
STANLEY         ATATACAAGGCCACTATGGAATATACATTTTGCATATATATAAGGCCACCAACAGTAATC
                ************************************************************

                        V                       v
Drysdale        GAGGCAAAATTAATGATATTTTCTCGTACTAACAACCTGTTTAATACTTGCATGCTTGCA
RAC875          GAGGCAAAATTAATGATATTTTCTCGTACTAACAACCTGTTTAATACTTGCATGCTTGCA
Norin61         GAGGCAAAATTAATGATATTTTCTCGTACTAACAACCTGTTTAATACTTGCATGCTTGCA
Jagger          GAGGCAAAATTAATGATATTTTCTCGTACTAACAACCTGTTTAATACTTGCATGCTTGCA
Mattis          GAGGCAAAATTAATGATATTTTCTCGTACTAACAACCTGTTTAATACTTGCATGCTTGCA
Julius          GAGGCAAAATTAATGATATTTTCTCGTACTAACAACCTGTTTAATACTTGCATGCTTGCA
Arina           GAGGCAAAATTAATGATATTTTCTCGTACTAACAACCTGTTTAATACTTGCATGCTTGCA
Spelta          GAGGCAAAATTAATGATATTTTCTCGTACTAACAACCTGTTTAATACTTGCATGCTTGCA
Lancer          GAGGCAAAATTAATGATATTTTCTCGTACTAACAACTTGTTTAATACTTGCATGCTTGCA
Gladius         GAGGCAAATTTAATGATATTTTCTCGTACTAATAACCTGTTTAATACTTGCATGCTTGCA
Kukri           GAGGCAAATTTAATGATATTTTCTCGTACTAATAACCTGTTTAATACTTGCATGCTTGCA
Landmark        GAGGCAAATTTAATGATATTTTCTCGTACTAATAACCTGTTTAATACTTGCATGCTTGCA
Mace            GAGGCAAATTTAATGATATTTTCTCGTACTAATAACCTGTTTAATACTTGCATGCTTGCA
STANLEY         GAGGCAAATTTAATGATATTTTCTCGTACTAATAACCTGTTTAATACTTGCATGCTTGCA
                ******** *********************** *** ***********************

                                                  v
Drysdale        ATCATAATGACAGTCAGCTACTTCCTCCACTCAGTTTTTTTTGCATGCATGTGGAGTATT
RAC875          ATCATAATGACAGTCAGCTACTTCCTCCACTCAGTTTTTTTTGCATGCATGTGGAGTATT
Norin61         ATCATAATGACAGTCAGCTACTTCCTCCACTCAGTTTTTTTTGCATGCATGTGGAGTATT
Jagger          ATCATAATGACAGTCAGCTACTTCCTCCACTCAGTTTTTTTTGCATGCATGTGGAGTATT
Mattis          ATCATAATGACAGTCAGCTACTTCCTCCACTCAGTTTTTTTTGCATGCATGTGGAGTATT
Julius          ATCATAATGACAGTCAGCTACTTCCTCCACTCAGTTTTTTTTGCATGCATGTGGAGTATT
Arina           ATCATAATGACAGTCAGCTACTTCCTCCACTCAGTTTTTTTTGCATGCATGTGGAGTATT
Spelta          ATCATAATGACAGTCAGCTACTTCCTCCACTCAGTTTTTTTTGCATGCATGTGGAGTATT
Lancer          ATCATAATGACAGTCAGCTACTTCCTCCACTCAG-TTTTTTTGCATGCATGTGGAGTATT
Gladius         ATCATAATGACAGTCAGCTACTTCCTCCACTCAG-TTTTTTTGCATGCATGTGGAGTATT
Kukri           ATCATAATGACAGTCAGCTACTTCCTCCACTCAG-TTTTTTTGCATGCATGTGGAGTATT
Landmark        ATCATAATGACAGTCAGCTACTTCCTCCACTCAG-TTTTTTTGCATGCATGTGGAGTATT
Mace            ATCATAATGACAGTCAGCTACTTCCTCCACTCAG-TTTTTTTGCATGCATGTGGAGTATT
STANLEY         ATCATAATGACAGTCAGCTACTTCCTCCACTCAG-TTTTTTTGCATGCATGTGGAGTATT
                ********************************** *************************

Drysdale        AATGATCCCAGTAAACAAGAAGAAAAGTTGACTTGTAAAGCAGGCATTAAATTTTACATT
RAC875          AATGATCCCAGTAAACAAGAAGAAAAGTTGACTTGTAAAGCAGGCATTAAATTTTACATT
Norin61         AATGATCCCAGTAAACAAGAAGAAAAGTTGACTTGTAAAGCAGGCATTAAATTTTACATT
Jagger          AATGATCCCAGTAAACAAGAAGAAAAGTTGACTTGTAAAGCAGGCATTAAATTTTACATT
Mattis          AATGATCCCAGTAAACAAGAAGAAAAGTTGACTTGTAAAGCAGGCATTAAATTTTACATT
Julius          AATGATCCCAGTAAACAAGAAGAAAAGTTGACTTGTAAAGCAGGCATTAAATTTTACATT
Arina           AATGATCCCAGTAAACAAGAAGAAAAGTTGACTTGTAAAGCAGGCATTAAATTTTACATT
Spelta          AATGATCCCAGTAAACAAGAAGAAAAGTTGACTTGTAAAGCAGGCATTAAATTTTACATT
Lancer          AATGATCCCAGTAAACAACAAGAAAAGTTGACTTGTAAAGCAGGCATTAAATTTTACATT
Gladius         AATGATCCCAGTAAACAAGAAGAAAAGTTGACTTGTAAAGCAGGCATTAAATTTTACATT
Kukri           AATGATCCCAGTAAACAAGAAGAAAAGTTGACTTGTAAAGCAGGCATTAAATTTTACATT
Landmark        AATGATCCCAGTAAACAAGAAGAAAAGTTGACTTGTAAAGCAGGCATTAAATTTTACATT
Mace            AATGATCCCAGTAAACAAGAAGAAAAGTTGACTTGTAAAGCAGGCATTAAATTTTACATT
STANLEY         AATGATCCCAGTAAACAAGAAGAAAAGTTGACTTGTAAAGCAGGCATTAAATTTTACATT
                ****************** *****************************************
```

```
                 v
Drysdale        GATACCTGTAATCCAAGTTTGTGGCCTTATATACAAAAATGGAGGGAGTATAAAAATCTA
RAC875          GATACCTGTAATCCAAGTTTGTGGCCTTATATACAAAAATGGAGGGAGTATAAAAATCTA
Norin61         GATACCTGTAATCCAAGTTTGTGGCCTTATATACAAAAATGGAGGGAGTATAAAAATCTA
Jagger          GATACCTGTAATCCAAGTTTGTGGCCTTATATACAAAAATGGAGGGAGTATAAAAATCTA
Mattis          GATACCTGTAATCCAAGTTTGTGGCCTTATATACAAAAATGGAGGGAGTATAAAAATCTA
Julius          GATACCTGTAATCCAAGTTTGTGGCCTTATATACAAAAATGGAGGGAGTATAAAAATCTA
Arina           GATACCTGTAATCCAAGTTTGTGGCCTTATATACAAAAATGGAGGGAGTATAAAAATCTA
Spelta          GATACCTGTAATCCAAGTTTGTGGCCTTATATACAAAAATGGAGGGAGTATAAAAATCTA
Lancer          GGTACCTGTAATCCAAGTTTGTGGCCTTATATACAAAAATGGAGGGAGTATAAAAATCTA
Gladius         GGTACCTGTAATCCAAGTTTGTGGCCTTATATACAAAAATGGAGGGAGTATAAAAATCTA
Kukri           GGTACCTGTAATCCAAGTTTGTGGCCTTATATACAAAAATGGAGGGAGTATAAAAATCTA
Landmark        GGTACCTGTAATCCAAGTTTGTGGCCTTATATACAAAAATGGAGGGAGTATAAAAATCTA
Mace            GGTACCTGTAATCCAAGTTTGTGGCCTTATATACAAAAATGGAGGGAGTATAAAAATCTA
STANLEY         GGTACCTGTAATCCAAGTTTGTGGCCTTATATACAAAAATGGAGGGAGTATAAAAATCTA
                * **********************************************************

Drysdale        GCAGCGCAAATGCGCGGATAACTGTGCTAACTACTCAATAAGAGGTTAAAAGAAGATTCA
RAC875          GCAGCGCAAATGCGCGGATAACTGTGCTAACTACTCAATAAGAGGTTAAAAGAAGATTCA
Norin61         GCAGCGCAAATGCGCGGATAACTGTGCTAACTACTCAATAAGAGGTTAAAAGAAGATTCA
Jagger          GCAGCGCAAATGCGCGGATAACTGTGCTAACTACTCAATAAGAGGTTAAAAGAAGATTCA
Mattis          GCAGCGCAAATGCGCGGATAACTGTGCTAACTACTCAATAAGAGGTTAAAAGAAGATTCA
Julius          GCAGCGCAAATGCGCGGATAACTGTGCTAACTACTCAATAAGAGGTTAAAAGAAGATTCA
Arina           GCAGCGCAAATGCGCGGATAACTGTGCTAACTACTCAATAAGAGGTTAAAAGAAGATTCA
Spelta          GCAGCGCAAATGCGCGGATAACTGTGCTAACTACTCAATAAGAGGTTAAAAGAAGATTCA
Lancer          GCAGCGCAAATGCGCGGATAACTGTGCTAACTACTCAATAAGAGGTTAAAAGAAGATTCA
Gladius         GCAGCGCAAATGCGCGGATAACTGTGCTAACTACTCAATAAGAGGTTAAAAGAAGATTCA
Kukri           GCAGCGCAAATGCGCGGATAACTGTGCTAACTACTCAATAAGAGGTTAAAAGAAGATTCA
Landmark        GCAGCGCAAATGCGCGGATAACTGTGCTAACTACTCAATAAGAGGTTAAAAGAAGATTCA
Mace            GCAGCGCAAATGCGCGGATAACTGTGCTAACTACTCAATAAGAGGTTAAAAGAAGATTCA
STANLEY         GCAGCGCAAATGCGCGGATAACTGTGCTAACTACTCAATAAGAGGTTAAAAGAAGATTCA
                ************************************************************

Drysdale        CATGCACAACTGTAGAAAACAAAAGGCCACACAACCAGTATTTAAAAAATGTACATTAAT
RAC875          CATGCACAACTGTAGAAAACAAAAGGCCACACAACCAGTATTTAAAAAATGTACATTAAT
Norin61         CATGCACAACTGTAGAAAACAAAAGGCCACACAACCAGTATTTAAAAAATGTACATTAAT
Jagger          CATGCACAACTGTAGAAAACAAAAGGCCACACAACCAGTATTTAAAAAATGTACATTAAT
Mattis          CATGCACAACTGTAGAAAACAAAAGGCCACACAACCAGTATTTAAAAAATGTACATTAAT
Julius          CATGCACAACTGTAGAAAACAAAAGGCCACACAACCAGTATTTAAAAAATGTACATTAAT
Arina           CATGCACAACTGTAGAAAACAAAAGGCCACACAACCAGTATTTAAAAAATGTACATTAAT
Spelta          CATGCACAACTGTAGAAAACAAAAGGCCACACAACCAGTATTTAAAAAATGTACATTAAT
Lancer          CATGCACAACTGTAGAAAACAAAAGGCCACACAACCAGTATTTAAAAAATGTACATTAAT
Gladius         CATGCACAACTGTAGAAAACAAAAGGCCACACAACCAGTATTTAAAAAATGTACATTAAT
Kukri           CATGCACAACTGTAGAAAACAAAAGGCCACACAACCAGTATTTAAAAAATGTACATTAAT
Landmark        CATGCACAACTGTAGAAAACAAAAGGCCACACAACCAGTATTTAAAAAATGTACATTAAT
Mace            CATGCACAACTGTAGAAAACAAAAGGCCACACAACCAGTATTTAAAAAATGTACATTAAT
STANLEY         CATGCACAACTGTAGAAAACAAAAGGCCACACAACCAGTATTTAAAAAATGTACATTAAT
                ************************************************************

Drysdale        TGGACTCCCTTCATTCCTACTTACCATGCACATATTTTTTTCTAGTTTTTCGTGATTATC
RAC875          TGGACTCCCTTCATTCCTACTTACCATGCACATATTTTTTTCTAGTTTTTCGTGATTATC
Norin61         TGGACTCCCTTCATTCCTACTTACCATGCACATATTTTTTTCTAGTTTTTCGTGATTATC
Jagger          TGGACTCCCTTCATTCCTACTTACCATGCACATATTTTTTTCTAGTTTTTCGTGATTATC
Mattis          TGGACTCCCTTCATTCCTACTTACCATGCACATATTTTTTTCTAGTTTTTCGTGATTATC
Julius          TGGACTCCCTTCATTCCTACTTACCATGCACATATTTTTTTCTAGTTTTTCGTGATTATC
Arina           TGGACTCCCTTCATTCCTACTTACCATGCACATATTTTTTTCTAGTTTTTCGTGATTATC
Spelta          TGGACTCCCTTCATTCCTACTTACCATGCACATATTTTTTTCTAGTTTTTCGTGATTATC
Lancer          TGGACTCCCTTCATTCCTACTTACCATGCACATATTTTTTTCTAGTTTTTCGTGATTATC
Gladius         TGGACTCCCTTCATTCCTACTTACCATGCACATATTTTTTTCTAGTTTTTCGTGATTATC
Kukri           TGGACTCCCTTCATTCCTACTTACCATGCACATATTTTTTTCTAGTTTTTCGTGATTATC
Landmark        TGGACTCCCTTCATTCCTACTTACCATGCACATATTTTTTTCTAGTTTTTCGTGATTATC
Mace            TGGACTCCCTTCATTCCTACTTACCATGCACATATTTTTTTCTAGTTTTTCGTGATTATC
STANLEY         TGGACTCCCTTCATTCCTACTTACCATGCACATATTTTTTTCTAGTTTTTCGTGATTATC
                ************************************************************
```

```
Drysdale        TAGGGTATTATGGTTCCCACACAGATGATTCTCTATCTTTCCAACCCTTATTTTGGGTAG
RAC875          TAGGGTATTATGGTTCCCACACAGATGATTCTCTATCTTTCCAACCCTTATTTTGGGTAG
Norin61         TAGGGTATTATGGTTCCCACACAGATGATTCTCTATCTTTCCAACCCTTATTTTGGGTAG
Jagger          TAGGGTATTATGGTTCCCACACAGATGATTCTCTATCTTTCCAACCCTTATTTTGGGTAG
Mattis          TAGGGTATTATGGTTCCCACACAGATGATTCTCTATCTTTCCAACCCTTATTTTGGGTAG
Julius          TAGGGTATTATGGTTCCCACACAGATGATTCTCTATCTTTCCAACCCTTATTTTGGGTAG
Arina           TAGGGTATTATGGTTCCCACACAGATGATTCTCTATCTTTCCAACCCTTATTTTGGGTAG
Spelta          TAGGGTATTATGGTTCCCACACAGATGATTCTCTATCTTTCCAACCCTTATTTTGGGTAG
Lancer          TAGGGTATTATGGTTCCCACACAGATGATTCTCTATCTTTCCAACCCTTATTTTGGGTAG
Gladius         TAGGGTATTATGGTTCCCACACAGATGATTCTCTATCTTTCCAACCCTTATTTTGGGTAG
Kukri           TAGGGTATTATGGTTCCCACACAGATGATTCTCTATCTTTCCAACCCTTATTTTGGGTAG
Landmark        TAGGGTATTATGGTTCCCACACAGATGATTCTCTATCTTTCCAACCCTTATTTTGGGTAG
Mace            TAGGGTATTATGGTTCCCACACAGATGATTCTCTATCTTTCCAACCCTTATTTTGGGTAG
STANLEY         TAGGGTATTATGGTTCCCACACAGATGATTCTCTATCTTTCCAACCCTTATTTTGGGTAG
                ************************************************************

                               v
Drysdale        TACTACCAATGCATATATTATCAATGTTCTTTCCATAAAATGCTCCCGTGACATTAACTA
RAC875          TACTACCAATGCATATATTATCAATGTTCTTTCCATAAAATGCTCCCGTGACATTAACTA
Norin61         TACTACCAATGCATATATTATCAATGTTCTTTCCATAAAATGCTCCCGTGACATTAACTA
Jagger          TACTACCAATGCATATATTATCAATGTTCTTTCCATAAAATGCTCCCGTGACATTAACTA
Mattis          TACTACCAATGCATATATTATCAATGTTCTTTCCATAAAATGCTCCCGTGACATTAACTA
Julius          TACTACCAATGCATATATTATCAATGTTCTTTCCATAAAATGCTCCCGTGACATTAACTA
Arina           TACTACCAATGCATATATTATCAATGTTCTTTCCATAAAATGCTCCCGTGACATTAACTA
Spelta          TACTACCAATGCATATATTATCAATGTTCTTTCCATAAAATGCTCCCGTGACATTAACTA
Lancer          TACTACCAATGCATATATTATCAATGTTCTTTCCATAAAATGCTCCCGTGACATTAACTA
Gladius         TACTACCAATGCATATATTATCAATGTTCTTCCCATAAAATGCTCCCGTGACATTAACTA
Kukri           TACTACCAATGCATATATTATCAATGTTCTTCCCATAAAATGCTCCCGTGACATTAACTA
Landmark        TACTACCAATGCATATATTATCAATGTTCTTCCCATAAAATGCTCCCGTGACATTAACTA
Mace            TACTACCAATGCATATATTATCAATGTTCTTCCCATAAAATGCTCCCGTGACATTAACTA
STANLEY         TACTACCAATGCATATATTATCAATGTTCTTCCCATAAAATGCTCCCGTGACATTAACTA
                ******************************* ****************************

Drysdale        TCCCACCATGCATGGGAGGAATTAATGCATGCATATCATTAACTATCTCAACGTCTAAAC
RAC875          TCCCACCATGCATGGAGGAAATTAATGCATGCATATCATTAACTATCTCAACGTCTAAAC
Norin61         TCCCACCATGCATGGAGGAAATTAATGCATGCATATCATTAACTATCTCAACGTCTAAAC
Jagger          TCCCACCATGCATGGAGGAAATTAATGCATGCATATCATTAACTATCTCAACGTCTAAAC
Mattis          TCCCACCATGCATGGAGGAAATTAATGCATGCATATCATTAACTATCTCAACGTCTAAAC
Julius          TCCCACCATGCATGGAGGAAATTAATGCATGCATATCATTAACTATCTCAACGTCTAAAC
Arina           TCCCACCATGCATGGAGGAAATTAATGCATGCATATCATTAACTATCTCAACGTCTAAAC
Spelta          TCCCACCATGCATGGAGGAAATTAATGCATGCATATCATTAACTATCTCAACGTCTAAAC
Lancer          TCCCACCATGCATGGAGGAAATTAATGCATGCATATCATTAACTATCTCAACGTCTAAAC
Gladius         TCCCACCATGCATGGAGGAAATTAATGCATGCATATCATTAACTATCTCAACGTCTAAAC
Kukri           TCCCACCATGCATGGAGGAAATTAATGCATGCATATCATTAACTATCTCAACGTCTAAAC
Landmark        TCCCACCATGCATGGAGGAAATTAATGCATGCATATCATTAACTATCTCAACGTCTAAAC
Mace            TCCCACCATGCATGGAGGAAATTAATGCATGCATATCATTAACTATCTCAACGTCTAAAC
STANLEY         TCCCACCATGCATGGAGGAAATTAATGCATGCATATCATTAACTATCTCAACGTCTAAAC
                ***************  * *****************************************

                       v
Drysdale        CGAAACA-GGCACCGCCAGATGCTGAGGGCAAGCTCGTCCAACTACACATCCAATTGTCA
RAC875          CGAAACAGGGCACCGCCAAATGCTGAGGGCAAGCTCGTCCAACTACACATCCAATTGTCA
Norin61         CGAAACAAGGCACCGCCAGATGCTGAGGGCAAGCTCGTCCAACTACACATCCAATTGTCA
Jagger          CGAAACAAGGCACCGCCAGATGCTGAGGGCAAGCTCGTCCAACTACACATCCAATTGTCA
Mattis          CGAAACAAGGCACCGCCAGATGCTGAGGGCAAGCTCGTCCAACTACACATCCAATTGTCA
Julius          CGAAACAAGGCACCGCCAGATGCTGAGGGCAAGCTCGTCCAACTACACATCCAATTGTCA
Arina           CGAAACAAGGCACCGCCAGATGCTGAGGGCAAGCTCGTCCAACTACACATCCAATTGTCA
Spelta          CGAAACAAGGCACCGCCAGATGCTGAGGGCAAGCTCGTCCAACTACACATCCAATTGTCA
Lancer          CGAAACAAGGCACCGCCAGATGCCGAGGGCAAGCTCGTCCAACTACACATCCAATTGTCA
Gladius         CGAAACAAGGCACCGCCAGATGCCGAGGGCAAGCTCGTCCAACTACACATCCAATTGTCA
Kukri           CGAAACAAGGCACCGCCAGATGCCGAGGGCAAGCTCGTCCAACTACACATCCAATTGTCA
Landmark        CGAAACAAGGCACCGCCAGATGCCGAGGGCAAGCTCGTCCAACTACACATCCAATTGTCA
Mace            CGAAACAAGGCACCGCCAGATGCCGAGGGCAAGCTCGTCCAACTACACATCCAATTGTCA
STANLEY         CGAAACAAGGCACCGCCAGATGCCGAGGGCAAGCTCGTCCAACTACACATCCAATTGTCA
                ******* ********** **** ************************************
```

```
                   v
Drysdale        ATGTCCACCACTCATTTATATGC-GGGCTTGAACTAAAAAA--TTAGATACTCCCTCCGT
RAC875          ATGTCCACCACTCATTTATATGCGGGGCTTGAACTAAAAAA--TTAGATACTCCCTCCGT
Norin61         ATGTCCACCACTCATTTATATGC-GGGCTTGAACTAAAAAA--TTAGATACTCCCTCCGT
Jagger          ATGTCCACCACTCATTTATATGC-GGGCTTGAACTAAAAAA--TTAGATACTCCCTCCGT
Mattis          ATGTCCACCACTCATTTATATGC-GGGCTTGAACTAAAAAA--TTAGATACTCCCTCCGT
Julius          ATGTCCACCACTCATTTATATGC-GGGCTTGAACTAAAAAA--TTAGATACTCCCTCCGT
Arina           ATGTCCACCACTCATTTATATGC-GGGCTTGAACTAAAAAA--TTAGATACTCCCTCCGT
Spelta          ATGTCCACCACTCATTTATATGC-GGGCTTGAACTAAAAAA--TTAGATACTCCCTCCGT
Lancer          ATCTCCACCACTCATTTATATGC-GGGCTTGAACTAAAAAAA-TTAGATACTCCCTCCGT
Gladius         ATCTCCACCACTCATTTATATGCGGGGCTTGAACTAAAAAAA---TGATACTCCCTCCGT
Kukri           ATCTCCACCACTCATTTATATGC-GGGCTTGAACTAAAAAAAATTAGATACTCCCTCCGT
Landmark        ATCTCCACCACTCATTTATATGC-GGGCTTGAACTAAAAAAA-TTAGATACTCCCTCCGT
Mace            ATCTCCACCACTCATTTATATGC-GGGCTTGAACTAAAAAAA-TTAGATACTCCCTCCGT
STANLEY         ATCTCCACCACTCATTTATATGC-GGGCTTGAACTAAAAAAA-TTAGATACTCCCTCCGT
                ** ******************** *****************     **************

Drysdale        CTAGGTGTGTAAGTCATCTTACGAAAACCAAATAATCCCAAAATAATTAGGCGTGGTGCA
RAC875          CTAGGTGTGTAAGTCATCTTACGAAAACCAAATAATCCCAAAATAATTAGGCGTGGTGCA
Norin61         CTAGGTGTGTAAGTCATCTTACGAAAACCAAATAATCCCAAAATAATTAGGCGTGGTGCA
Jagger          CTAGGTGTGTAAGTCATCTTACGAAAACCAAATAATCCCAAAATAATTAGGCGTGGTGCA
Mattis          CTAGGTGTGTAAGTCATCTTACGAAAACCAAATAATCCCAAAATAATTAGGCGTGGTGCA
Julius          CTAGGTGTGTAAGTCATCTTACGAAAACCAAATAATCCCAAAATAATTAGGCGTGGTGCA
Arina           CTAGGTGTGTAAGTCATCTTACGAAAACCAAATAATCCCAAAATAATTAGGCGTGGTGCA
Spelta          CTAGGTGTGTAAGTCATCTTACGAAAACCAAATAATCCCAAAATAATTAGGCGTGGTGCA
Lancer          CTAGGTGTGTAAGTCATCTTACGAAAACCAAATAATCCCAAAATAATTAGGCGTGGTGCA
Gladius         CTAGGTGTGTAAGTCATCTTACGAAAACCAAATAATCCCAAAATAATTAGGCGTGGTGCA
Kukri           CTAGGTGTGTAAGTCATCTTACGAAAACCAAATAATCCCAAAATAATTAGGCGTGGTGCA
Landmark        CTAGGTGTGTAAGTCATCTTACGAAAACCAAATAATCCCAAAATAATTAGGCGTGGTGCA
Mace            CTAGGTGTGTAAGTCATCTTACGAAAACCAAATAATCCCAAAATAATTAGGCGTGGTGCA
STANLEY         CTAGGTGTGTAAGTCATCTTACGAAAACCAAATAATCCCAAAATAATTAGGCGTGGTGCA
                ************************************************************
                              v       v
Drysdale        TTAACTTCTACCTCATTTCTTG-TTTTTGACATATCAACCAATAATAGATGAGGGTGTGC
RAC875          TTAACTTCTACCTCATTTCTTG-TTTTTGACATATCAACCAATAATAGATGAGGGTGTGC
Norin61         TTAACTTCTACCTCATTTCTTG-TTTTTGACATATCAACCAATAATAGATGAGGGTGTGC
Jagger          TTAACTTCTACCTCATTTCTTG-TTTTTGACATATCAACCAATAATAGATGAGGGTGTGC
Mattis          TTAACTTCTACCTCATTTCTTG-TTTTTGACATATCAACCAATAATAGATGAGGGTGTGC
Julius          TTAACTTCTACCTCATTTCTTG-TTTTTGACATATCAACCAATAATAGATGAGGGTGTGC
Arina           TTAACTTCTACCTCATTTCTTG-TTTTTGACATATCAACCAATAATAGATGAGGGTGTGC
Spelta          TTAACTTCTACCTCATTTCTTG-TTTTTGACATATCAACCAATAATAGATGAGGGTGTGC
Lancer          TTAACTTCTACCTCGTTTCTTGTTTTTTGACATATCAACCAATAATAGATGAGGGTGTGC
Gladius         TTAACTTCTACCTCGTTTC-TGTTTTTTGACATATCAACCAATAATAGATGAGGGTGTGC
Kukri           TTAACTTCTACCTCGTTTCTTGTTTTTTGACATATCAACCAATAATAGATGAGGGTGTGC
Landmark        TTAACTTCTACCTCGTTTCTTGTTTTTTGACATATCAACCAATAATAGATGAGGGTGTGC
Mace            TTAACTTCTACCTCGTTTCTTGTTTTTTGACATATCAACCAATAATAGATGAGGGTGTGC
STANLEY         TTAACTTCTACCTCGTTTCTTGTTTTTTGACATATCAACCAATAATAGATGAGGGTGTGC
                ************** **** ** *************************************

Drysdale        ATGCTTTTAATGACTTGAGACTATTAAACACGACATGCAGTGGTTAGTTCATTGCATGCA
RAC875          ATGCTTTTAATGACTTGAGACTATTAAACACGACATGCAGTGGTTAGTTCATTGCATGCA
Norin61         ATGCTTTTAATGACTTGAGACTATTAAACACGACATGCAGTGGTTAGTTCATTGCATGCA
Jagger          ATGCTTTTAATGACTTGAGACTATTAAACACGACATGCAGTGGTTAGTTCATTGCATGCA
Mattis          ATGCTTTTAATGACTTGAGACTATTAAACACGACATGCAGTGGTTAGTTCATTGCATGCA
Julius          ATGCTTTTAATGACTTGAGACTATTAAACACGACATGCAGTGGTTAGTTCATTGCATGCA
Arina           ATGCTTTTAATGACTTGAGACTATTAAACACGACATGCAGTGGTTAGTTCATTGCATGCA
Spelta          ATGCTTTTAATGACTTGAGACTATTAAACACGACATGCAGTGGTTAGTTCATTGCATGCA
Lancer          ATGCTTTTAATGACTTGAGACTATTAAACACGACATGCAGTGGTTAGTTCATTGCATGCA
Gladius         ATGCTTTTAATGACTTGAGACTATTAAACACGACATGCAGTGGTTAGTTCATTGCATGCA
Kukri           ATGCTTTTAATGACTTGAGACTATTAAACACGACATGCAGTGGTTAGTTCATTGCATGCA
Landmark        ATGCTTTTAATGACTTGAGACTATTAAACACGACATGCAGTGGTTAGTTCATTGCATGCA
Mace            ATGCTTTTAATGACTTGAGACTATTAAACACGACATGCAGTGGTTAGTTCATTGCATGCA
STANLEY         ATGCTTTTAATGACTTGAGACTATTAAACACGACATGCAGTGGTTAGTTCATTGCATGCA
                ************************************************************
```

```
Drysdale        ATACTATTAATTAGCAAATAAACATTAATGTCTCTCATTTTCCCCTCCTCCTTGGTCACG
RAC875          ATACTATTAATTAGCAAATAAACATTAATGTCTCTCATTTTCCCCTCCTCCTTGGTCACG
Norin61         ATACTATTAATTAGCAAATAAACATTAATGTCTCTCATTTTCCCCTCCTCCTTGGTCACG
Jagger          ATACTATTAATTAGCAAATAAACATTAATGTCTCTCATTTTCCCCTCCTCCTTGGTCACG
Mattis          ATACTATTAATTAGCAAATAAACATTAATGTCTCTCATTTTCCCCTCCTCCTTGGTCACG
Julius          ATACTATTAATTAGCAAATAAACATTAATGTCTCTCATTTTCCCCTCCTCCTTGGTCACG
Arina           ATACTATTAATTAGCAAATAAACATTAATGTCTCTCATTTTCCCCTCCTCCTTGGTCACG
Spelta          ATACTATTAATTAGCAAATAAACATTAATGTCTCTCATTTTCCCCTCCTCCTTGGTCACG
Lancer          ATACTATTAATTAGCAAATAAACATTAATGTCTCTCATTTTCCCCTCCTCCTTGGTCACG
Gladius         ATACTATTAATTAGCAAATAAACATTAATGTCTCTCATTTTCCCCTCCTCCTTGGTCACG
Kukri           ATACTATTAATTAGCAAATAAACATTAATGTCTCTCATTTTCCCCTCCTCCTTGGTCACG
Landmark        ATACTATTAATTAGCAAATAAACATTAATGTCTCTCATTTTCCCCTCCTCCTTGGTCACG
Mace            ATACTATTAATTAGCAAATAAACATTAATGTCTCTCATTTTCCCCTCCTCCTTGGTCACG
STANLEY         ATACTATTAATTAGCAAATAAACATTAATGTCTCTCATTTTCCCCTCCTCCTTGGTCACG
                ************************************************************

                                          v
Drysdale        GTGCACAGCCTAAGATGACTTACTCACGTAGACGGAGGGAGTACTCACTTCAGATGGGAA
RAC875          GTGCACAGCCTAAGATGACTTACTCACGTAGACGGAGGGAGTACTCACTTCAGATGGGAA
Norin61         GTGCACAGCCTAAGATGACTTACTCACGTAGACGGAGGGAGTACTCACTTCAGATGGGAA
Jagger          GTGCACAGCCTAAGATGACTTACTCACGTAGACGGAGGGAGTACTCACTTCAGATGGGAA
Mattis          GTGCACAGCCTAAGATGACTTACTCACGTAGACGGAGGGAGTACTCACTTCAGATGGGAA
Julius          GTGCACAGCCTAAGATGACTTACTCACGTAGACGGAGGGAGTACTCACTTCAGATGGGAA
Arina           GTGCACAGCCTAAGATGACTTACTCACGTAGACGGAGGGAGTACTCACTTCAGATGGGAA
Spelta          GTGCACAGCCTAAGATGACTTACTCACGTAGACGGAGGGAGTACTCACTTCAGATGGGAA
Lancer          GTGCACAGCCTAAGATGACTTACTCACCTAGACGGAGGGAGTACTCACTTCAGATGGGAA
Gladius         GTGCACAGCCTAAGATGACTTACTCACCTAGACGGAGGGAGTACTCACTTCAGATGGGAA
Kukri           GTGCACAGCCTAAGATGACTTACTCACCTAGACGGAGGGAGTACTCACTTCAGATGGGAA
Landmark        GTGCACAGCCTAAGATGACTTACTCACCTAGACGGAGGGAGTACTCACTTCAGATGGGAA
Mace            GTGCACAGCCTAAGATGACTTACTCACCTAGACGGAGGGAGTACTCACTTCAGATGGGAA
STANLEY         GTGCACAGCCTAAGATGACTTACTCACCTAGACGGAGGGAGTACTCACTTCAGATGGGAA
                ************************** *********************************

                              v
Drysdale        TACAAGTCGGGCACATTTTCTAGGTCTTCAATTTGACAAGCGAATAATATGCCACAAAAA
RAC875          TACAAGTCGGGCACATTTTCTAGGTCTTCAATTTGACAAGCGAATAATATGCCACAAAAA
Norin61         TACAAGTCGGGCACATTTTCTAGGTCTTCAATTTGACAAGCGAATAATATGCCACAAAAA
Jagger          TACAAGTCGGGCACATTTTCTAGGTCTTCAATTTGACAAGCGAATAATATGCCACAAAAA
Mattis          TACAAGTCGGGCACATTTTCTAGGTCTTCAATTTGACAAGCGAATAATATGCCACAAAAA
Julius          TACAAGTCGGGCACATTTTCTAGGTCTTCAATTTGACAAGCGAATAATATGCCACAAAAA
Arina           TACAAGTCGGGCACATTTTCTAGGTCTTCAATTTGACAAGCGAATAATATGCCACAAAAA
Spelta          TACAAGTCGGGCACATTTTCTAGGTCTTCAATTTGACAAGCGAATAATATGCCACAAAAA
Lancer          TACAAGTCGGGCACAGTTTCTAGGTCTTCAATTTGACAAGCGAATAATATGCCACAAAAA
Gladius         TACAAGTCGGGCACAGTTTCTAGGTCTTCAATTTGACAAGCGAATAATATGCCACAAAAA
Kukri           TACAAGTCGGGCACAGTTTCTAGGTCTTCAATTTGACAAGCGAATAATATGCCACAAAAA
Landmark        TACAAGTCGGGCACAGTTTCTAGGTCTTCAATTTGACAAGCGAATAATATGCCACAAAAA
Mace            TACAAGTCGGGCACAGTTTCTAGGTCTTCAATTTGACAAGCGAATAATATGCCACAAAAA
STANLEY         TACAAGTCGGGCACAGTTTCTAGGTCTTCAATTTGACAAGCGAATAATATGCCACAAAAA
                ************** *********************************************

Drysdale        AATGGTAAGAAACCTCACCTGGCAAGGAATATACATTTTTTTGTGGCATATAACATATATT
RAC875          AATGGTAAGAAACCTCACCTGGCAAGGAATATACATTTTTTTGTGGCATATAACATATATT
Norin61         AATGGTAAGAAACCTCACCTGGCAAGGAATATACATTTTTTTGTGGCATATAACATATATT
Jagger          AATGGTAAGAAACCTCACCTGGCAAGGAATATACATTTTTTTGTGGCATATAACATATATT
Mattis          AATGGTAAGAAACCTCACCTGGCAAGGAATATACATTTTTTTGTGGCATATAACATATATT
Julius          AATGGTAAGAAACCTCACCTGGCAAGGAATATACATTTTTTTGTGGCATATAACATATATT
Arina           AATGGTAAGAAACCTCACCTGGCAAGGAATATACATTTTTTTGTGGCATATAACATATATT
Spelta          AATGGTAAGAAACCTCACCTGGCAAGGAATATACATTTTTTTGTGGCATATAACATATATT
Lancer          AATGGTAAGAAACCTCACCTGGCAAGGAATATACATTTTTTTGTGGCATATAACATATATT
Gladius         AATGGTAAGAAACCTCACCTGGCAAGGAATATACATTTTTTTGTGGCATATAACATATATT
Kukri           AATGGTAAGAAACCTCACCTGGCAAGGAATATACATTTTTTTGTGGCATATAACATATATT
Landmark        AATGGTAAGAAACCTCACCTGGCAAGGAATATACATTTTTTTGTGGCATATAACATATATT
Mace            AATGGTAAGAAACCTCACCTGGCAAGGAATATACATTTTTTTGTGGCATATAACATATATT
STANLEY         AATGGTAAGAAACCTCACCTGGCAAGGAATATACATTTTTTTGTGGCATATAACATATATT
                ************************************************************
```

```
                                  v
Drysdale        TTGCTTGTCAAATAGATATCCTAAAAATCGGTGTCTGTGTATATTCCCACCCAAAGGGAG
RAC875          TTGCTTGTCAAATAGATATCCTAAAAATCGGTGTCTGTGTATATTCCCACCCAAAGGGAG
Norin61         TTGCTTGTCAAATAGATATCCTAAAAATCGGTGTCTGTGTATATTCCCACCCAAAGGGAG
Jagger          TTGCTTGTCAAATAGATATCCTAAAAATCGGTGTCTGTGTATATTCCCACCCAAAGGGAG
Mattis          TTGCTTGTCAAATAGATATCCTAAAAATCGGTGTCTGTGTATATTCCCACCCAAAGGGAG
Julius          TTGCTTGTCAAATAGATATCCTAAAAATCGGTGTCTGTGTATATTCCCACCCAAAGGGAG
Arina           TTGCTTGTCAAATAGATATCCTAAAAATCGGTGTCTGTGTATATTCCCACCCAAAGGGAG
Spelta          TTGCTTGTCAAATAGATATCCTAAAAATCGGTGTCTGTGTATATTCCCACCCAAAGGGAG
Lancer          TTGCTTGTCAAATAGATAACCTAAAAATCGGTGTCTGTGTATATTCCCACCCAAAGGGAG
Gladius         TTGCTTGTCAAATAGATAACCTAAAAATCGGTGTCTGTGTATATTCCCACCCAAAGGGAG
Kukri           TTGCTTGTCAAATAGATAACCTAAAAATCGGTGTCTGTGTATATTCCCACCCAAAGGGAG
Landmark        TTGCTTGTCAAATAGATAACCTAAAAATCGGTGTCTGTGTATATTCCCACCCAAAGGGAG
Mace            TTGCTTGTCAAATAGATAACCTAAAAATCGGTGTCTGTGTATATTCCCACCCAAAGGGAG
STANLEY         TTGCTTGTCAAATAGATAACCTAAAAATCGGTGTCTGTGTATATTCCCACCCAAAGGGAG
                ****************** *****************************************

                                                                        v
Drysdale        TAACAATTAGGAATTAAGAGAGCTTTTATTATGGTAGACCCTTCTTTCATTAAGATACCC
RAC875          TAACAATTAGGAATTAAGAGAGCTTTTATTATGGTAGACCCTTCTTTCATTAAGATACCC
Norin61         TAACAATTAGGAATTAAGAGAGCTTTTATTATGGTAGACCCTTCTTTCATTAAGATACCC
Jagger          TAACAATTAGGAATTAAGAGAGCTTTTATTATGGTAGACCCTTCTTTCATTAAGATACCC
Mattis          TAACAATTAGGAATTAAGAGAGCTTTTATTATGGTAGACCCTTCTTTCATTAAGATACCC
Julius          TAACAATTAGGAATTAAGAGAGCTTTTATTATGGTAGACCCTTCTTTCATTAAGATACCC
Arina           TAACAATTAGGAATTAAGAGAGCTTTTATTATGGTAGACCCTTCTTTCATTAAGATACCC
Spelta          TAACAATTAGGAATTAAGAGAGCTTTTATTATGGTAGACCCTTCTTTCATTAAGATACCC
Lancer          TAACAATTAGGAATTAAGAGAGCTTTTATTATGGTAGACCCTTCTTTCATTAAGATACCC
Gladius         TAACAATTAGGAATTAAGAGAGCTTTTATTATGGTAGACCCTTCTTTCATTAAGATGCCC
Kukri           TAACAATTAGGAATTAAGAGAGCTTTTATTATGGTAGACCCTTCTTTCATTAAGATGCCC
Landmark        TAACAATTAGGAATTAAGAGAGCTTTTATTATGGTAGACCCTTCTTTCATTAAGATGCCC
Mace            TAACAATTAGGAATTAAGAGAGCTTTTATTATGGTAGACCCTTCTTTCATTAAGATGCCC
STANLEY         TAACAATTAGGAATTAAGAGAGCTTTTATTATGGTAGACCCTTCTTTCATTAAGATGCCC
                ******************************************************** ***

Drysdale        ACGTCTAAGGACATCATCAATGTATCATTCTTAAGTCGGTAATTAGCTGGGGCCACACTA
RAC875          ACGTCTAAGGACATCATCAATGTATCATTCTTAAGTCGGTAATTAGCTGGGGCCACACTA
Norin61         ACGTCTAAGGACATCATCAATGTATCATTCTTAAGTCGGTAATTAGCTGGGGCCACACTA
Jagger          ACGTCTAAGGACATCATCAATGTATCATTCTTAAGTCGGTAATTAGCTGGGGCCACACTA
Mattis          ACGTCTAAGGACATCATCAATGTATCATTCTTAAGTCGGTAATTAGCTGGGGCCACACTA
Julius          ACGTCTAAGGACATCATCAATGTATCATTCTTAAGTCGGTAATTAGCTGGGGCCACACTA
Arina           ACGTCTAAGGACATCATCAATGTATCATTCTTAAGTCGGTAATTAGCTGGGGCCACACTA
Spelta          ACGTCTAAGGACATCATCAATGTATCATTCTTAAGTCGGTAATTAGCTGGGGCCACACTA
Lancer          ACGTCTAAGGACATCATCAATGTATCATTCTTAAGTCGGTAATTAGCTGGGGCCACACTA
Gladius         ACGTCTAAGGACATCATCAATGTATCATTCTTAAGTCGGTAATTAGCTGGGGCCACACTA
Kukri           ACGTCTAAGGACATCATCAATGTATCATTCTTAAGTCGGTAATTAGCTGGGGCCACACTA
Landmark        ACGTCTAAGGACATCATCAATGTATCATTCTTAAGTCGGTAATTAGCTGGGGCCACACTA
Mace            ACGTCTAAGGACATCATCAATGTATCATTCTTAAGTCGGTAATTAGCTGGGGCCACACTA
STANLEY         ACGTCTAAGGACATCATCAATGTATCATTCTTAAGTCGGTAATTAGCTGGGGCCACACTA
                ************************************************************

Drysdale        TGCTATAGCAAACTCGAATTCCTCCTATGCTTAATTCCAATATGGCTATGGACGTTAGGG
RAC875          TGCTATAGCAAACTCGAATTCCTCCTATGCTTAATTCCAATATGGCTATGGACGTTAGGG
Norin61         TGCTATAGCAAACTCGAATTCCTCCTATGCTTAATTCCAATATGGCTATGGACGTTAGGG
Jagger          TGCTATAGCAAACTCGAATTCCTCCTATGCTTAATTCCAATATGGCTATGGACGTTAGGG
Mattis          TGCTATAGCAAACTCGAATTCCTCCTATGCTTAATTCCAATATGGCTATGGACGTTAGGG
Julius          TGCTATAGCAAACTCGAATTCCTCCTATGCTTAATTCCAATATGGCTATGGACGTTAGGG
Arina           TGCTATAGCAAACTCGAATTCCTCCTATGCTTAATTCCAATATGGCTATGGACGTTAGGG
Spelta          TGCTATAGCAAACTCGAATTCCTCCTATGCTTAATTCCAATATGGCTATGGACGTTAGGG
Lancer          TGCTATAGCAAACTCGAATTCCTCCTATGCTTAATTCCAATATGGCTATGGACGTTAGGG
Gladius         TGCTATAGCAAACTCGAATTCCTCCTATGCTTAATTCCAATATGGCTATGGACGTTAGGG
Kukri           TGCTATAGCAAACTCGAATTCCTCCTATGCTTAATTCCAATATGGCTATGGACGTTAGGG
Landmark        TGCTATAGCAAACTCGAATTCCTCCTATGCTTAATTCCAATATGGCTATGGACGTTAGGG
Mace            TGCTATAGCAAACTCGAATTCCTCCTATGCTTAATTCCAATATGGCTATGGACGTTAGGG
STANLEY         TGCTATAGCAAACTCGAATTCCTCCTATGCTTAATTCCAATATGGCTATGGACGTTAGGG
                ************************************************************
```

```
Drysdale        TGAAGCCCACTTGCCGGTATTTTCTCTTGTAGTTTTAACCCCTCAAATCATGTGAGGGGA
RAC875          TGAAGCCCACTTGCCGGTATTTTCTCTTGTAGTTTTAACCCCTCAAATCATGTGAGGGGA
Norin61         TGAAGCCCACTTGCCGGTATTTTCTCTTGTAGTTTTAACCCCTCAAATCATGTGAGGGGA
Jagger          TGAAGCCCACTTGCCGGTATTTTCTCTTGTAGTTTTAACCCCTCAAATCATGTGAGGGGA
Mattis          TGAAGCCCACTTGCCGGTATTTTCTCTTGTAGTTTTAACCCCTCAAATCATGTGAGGGGA
Julius          TGAAGCCCACTTGCCGGTATTTTCTCTTGTAGTTTTAACCCCTCAAATCATGTGAGGGGA
Arina           TGAAGCCCACTTGCCGGTATTTTCTCTTGTAGTTTTAACCCCTCAAATCATGTGAGGGGA
Spelta          TGAAGCCCACTTGCCGGTATTTTCTCTTGTAGTTTTAACCCCTCAAATCATGTGAGGGGA
Lancer          TGAAGCCCACTTGCCGGTATTTTCTCTTGTAGTTTTAACCCCTCAAATCATGTGAGGGGA
Gladius         TGAAGCCCACTTGCCGGTATTTTCTCTTGTAGTTTTAACCCCTCAAATCATGTGAGGGGA
Kukri           TGAAGCCCACTTGCCGGTATTTTCTCTTGTAGTTTTAACCCCTCAAATCATGTGAGGGGA
Landmark        TGAAGCCCACTTGCCGGTATTTTCTCTTGTAGTTTTAACCCCTCAAATCATGTGAGGGGA
Mace            TGAAGCCCACTTGCCGGTATTTTCTCTTGTAGTTTTAACCCCTCAAATCATGTGAGGGGA
STANLEY         TGAAGCCCACTTGCCGGTATTTTCTCTTGTAGTTTTAACCCCTCAAATCATGTGAGGGGA
                ************************************************************

Drysdale        GAAAGAAATAGTTTTTGATTCCTTGTGGCATGGCCTTAGCACTATGACCGGTCTTAAAGA
RAC875          GAAAGAAATAGTTTTTGATTCCTTGTGGCATGGCCTTAGCACTATGACCGGTCTTAAAGA
Norin61         GAAAGAAATAGTTTTTGATTCCTTGTGGCATGGCCTTAGCACTATGACCGGTCTTAAAGA
Jagger          GAAAGAAATAGTTTTTGATTCCTTGTGGCATGGCCTTAGCACTATGACCGGTCTTAAAGA
Mattis          GAAAGAAATAGTTTTTGATTCCTTGTGGCATGGCCTTAGCACTATGACCGGTCTTAAAGA
Julius          GAAAGAAATAGTTTTTGATTCCTTGTGGCATGGCCTTAGCACTATGACCGGTCTTAAAGA
Arina           GAAAGAAATAGTTTTTGATTCCTTGTGGCATGGCCTTAGCACTATGACCGGTCTTAAAGA
Spelta          GAAAGAAATAGTTTTTGATTCCTTGTGGCATGGCCTTAGCACTATGACCGGTCTTAAAGA
Lancer          GAAAGAAATAGTTTTTGATTCCTTGTGGCATGGCCTTAGCACTATGACCGGTCTTAAAGA
Gladius         GAAAGAAATAGTTTTTGATTCCTTGTGGCATGGCCTTAGCACTATGACCGGTCTTAAAGA
Kukri           GAAAGAAATAGTTTTTGATTCCTTGTGGCATGGCCTTAGCACTATGACCGGTCTTAAAGA
Landmark        GAAAGAAATAGTTTTTGATTCCTTGTGGCATGGCCTTAGCACTATGACCGGTCTTAAAGA
Mace            GAAAGAAATAGTTTTTGATTCCTTGTGGCATGGCCTTAGCACTATGACCGGTCTTAAAGA
STANLEY         GAAAGAAATAGTTTTTGATTCCTTGTGGCATGGCCTTAGCACTATGACCGGTCTTAAAGA
                ************************************************************

Drysdale        TTTTTGTTGCTAGGACCCCTTCCCACAATGCTTGAGGATATGTCATTAGACCCATATTTT
RAC875          TTTTTGTTGCTAGGACCCCTTCCCACAATGCTTGAGGATATGTCATTAGACCCATATTTT
Norin61         TTTTTGTTGCTAGGACCCCTTCCCACAATGCTTGAGGATATGTCATTAGACCCATATTTT
Jagger          TTTTTGTTGCTAGGACCCCTTCCCACAATGCTTGAGGATATGTCATTAGACCCATATTTT
Mattis          TTTTTGTTGCTAGGACCCCTTCCCACAATGCTTGAGGATATGTCATTAGACCCATATTTT
Julius          TTTTTGTTGCTAGGACCCCTTCCCACAATGCTTGAGGATATGTCATTAGACCCATATTTT
Arina           TTTTTGTTGCTAGGACCCCTTCCCACAATGCTTGAGGATATGTCATTAGACCCATATTTT
Spelta          TTTTTGTTGCTAGGACCCCTTCCCACAATGCTTGAGGATATGTCATTAGACCCATATTTT
Lancer          TTTTTGTTGCTAGGACCCCTTCCCACAATGCTTGAGGATATGTCATTAGACCCATATTTT
Gladius         TTTTTGTTGCTAGGACCCCTTCCCACAATGCTTGAGGATATGTCATTAGACCCATATTTT
Kukri           TTTTTGTTGCTAGGACCCCTTCCCACAATGCTTGAGGATATGTCATTAGACCCATATTTT
Landmark        TTTTTGTTGCTAGGACCCCTTCCCACAATGCTTGAGGATATGTCATTAGACCCATATTTT
Mace            TTTTTGTTGCTAGGACCCCTTCCCACAATGCTTGAGGATATGTCATTAGACCCATATTTT
STANLEY         TTTTTGTTGCTAGGACCCCTTCCCACAATGCTTGAGGATATGTCATTAGACCCATATTTT
                ************************************************************

Drysdale        TGCAACAATGGAGACATAATATAAGATTTGATGATATATAGGTTATATTTCATTAGAGAT
RAC875          TGCAACAATGGAGACATAATATAAGATTTGATGATATATAGGTTATATTTCATTAGAGAT
Norin61         TGCAACAATGGAGACATAATATAAGATTTGATGATATATAGGTTATATTTCATTAGAGAT
Jagger          TGCAACAATGGAGACATAATATAAGATTTGATGATATATAGGTTATATTTCATTAGAGAT
Mattis          TGCAACAATGGAGACATAATATAAGATTTGATGATATATAGGTTATATTTCATTAGAGAT
Julius          TGCAACAATGGAGACATAATATAAGATTTGATGATATATAGGTTATATTTCATTAGAGAT
Arina           TGCAACAATGGAGACATAATATAAGATTTGATGATATATAGGTTATATTTCATTAGAGAT
Spelta          TGCAACAATGGAGACATAATATAAGATTTGATGATATATAGGTTATATTTCATTAGAGAT
Lancer          TGCAACAATGGAGACATAATATAAGATTTGATGATATATAGGTTATATTTCATTAGAGAT
Gladius         TGCAACAATGGAGACATAATATAAGATTTGATGATATATAGGTTATATTTCATTAGAGAT
Kukri           TGCAACAATGGAGACATAATATAAGATTTGATGATATATAGGTTATATTTCATTAGAGAT
Landmark        TGCAACAATGGAGACATAATATAAGATTTGATGATATATAGGTTATATTTCATTAGAGAT
Mace            TGCAACAATGGAGACATAATATAAGATTTGATGATATATAGGTTATATTTCATTAGAGAT
STANLEY         TGCAACAATGGAGACATAATATAAGATTTGATGATATATAGGTTATATTTCATTAGAGAT
                ************************************************************
```

```
Drysdale        GATCTCTTAGGCCTGTCTCGATGGATATTATCATAAACTATTATCATTAAATGATGCTAC
RAC875          GATCTCTTAGGCCTGTCTCGATGGATATTATCATAAACTATTATCATTAAATGATGCTAC
Norin61         GATCTCTTAGGCCTGTCTCGATGGATATTATCATAAACTATTATCATTAAATGATGCTAC
Jagger          GATCTCTTAGGCCTGTCTCGATGGATATTATCATAAACTATTATCATTAAATGATGCTAC
Mattis          GATCTCTTAGGCCTGTCTCGATGGATATTATCATAAACTATTATCATTAAATGATGCTAC
Julius          GATCTCTTAGGCCTGTCTCGATGGATATTATCATAAACTATTATCATTAAATGATGCTAC
Arina           GATCTCTTAGGCCTGTCTCGATGGATATTATCATAAACTATTATCATTAAATGATGCTAC
Spelta          GATCTCTTAGGCCTGTCTCGATGGATATTATCATAAACTATTATCATTAAATGATGCTAC
Lancer          GATCTCTTAGGCCTGTCTCGATGGATACTATCATAAACTATTATCATTAAATGATGCTAC
Gladius         GATCTCTTAGGCCTGTCTCGATGGATATTATCATAAACTATTATCATTAAATGATGCTAC
Kukri           GATCTCTTAGGCCTGTCTCGATGGATATTATCATAAACTATTATCATTAAATGATGCTAC
Landmark        GATCTCTTAGGCCTGTCTCGATGGATATTATCATAAACTATTATCATTAAATGATGCTAC
Mace            GATCTCTTAGGCCTGTCTCGATGGATATTATCATAAACTATTATCATTAAATGATGCTAC
STANLEY         GATCTCTTAGGCCTGTCTCGATGGATATTATCATAAACTATTATCATTAAATGATGCTAC
                *************************** ********************************

Drysdale        TTTGCTTACTACATTCCCATCATTTCATAGCATCATAACATATATTTTACTGGGAATAAA
RAC875          TTTGCTTACTACATTCCCATCATTTCATAGCATCATAACATATATTTTACTGGGAATAAA
Norin61         TTTGCTTACTACATTCCCATCATTTCATAGCATCATAACATATATTTTACTGGGAATAAA
Jagger          TTTGCTTACTACATTCCCATCATTTCATAGCATCATAACATATATTTTACTGGGAATAAA
Mattis          TTTGCTTACTACATTCCCATCATTTCATAGCATCATAACATATATTTTACTGGGAATAAA
Julius          TTTGCTTACTACATTCCCATCATTTCATAGCATCATAACATATATTTTACTGGGAATAAA
Arina           TTTGCTTACTACATTCCCATCATTTCATAGCATCATAACATATATTTTACTGGGAATAAA
Spelta          TTTGCTTACTACATTCCCATCATTTCATAGCATCATAACATATATTTTACTGGGAATAAA
Lancer          TTTGCTTACTACATTCCCATCATTTCATAGCATCATAACATATATTTTACTGGGAATAAA
Gladius         TTTGCTTACTACATTCCCATCATTTCATAGCATCATAACATATATTTTACTGGGAATAAA
Kukri           TTTGCTTACTACATTCCCATCATTTCATAGCATCATAACATATATTTTACTGGGAATAAA
Landmark        TTTGCTTACTACATTCCCATCATTTCATAGCATCATAACATATATTTTACTGGGAATAAA
Mace            TTTGCTTACTACATTCCCATCATTTCATAGCATCATAACATATATTTTACTGGGAATAAA
STANLEY         TTTGCTTACTACATTCCCATCATTTCATAGCATCATAACATATATTTTACTGGGAATAAA
                ************************************************************

Drysdale        CTTATATTTTATAACTTAAAATTATTATCAAAATGTGACAAAATACAAGAGGACTAGTGA
RAC875          CTTATATTTTATAACTTAAAATTATTATCAAAATGTGACAAAATACAAGAGGACTAGTGA
Norin61         CTTATATTTTGTAACTTAAAATTATTATCAAAATGTGACAAAATACAAGAGGACTAGTGA
Jagger          CTTATATTTTATAACTTAAAATTATTATCAAAATGTGACAAAATACAAGAGGACTAGTGA
Mattis          CTTATATTTTATAACTTAAAATTATTATCAAAATGTGACAAAATACAAGAGGACTAGTGA
Julius          CTTATATTTTATAACTTAAAATTATTATCAAAATGTGACAAAATACAAGAGGACTAGTGA
Arina           CTTATATTTTATAACTTAAAATTATTATCAAAATGTGACAAAATACAAGAGGACTAGTGA
Spelta          CTTATATTTTATAACTTAAAATTATTATCAAAATGTGACAAAATACAAGAGGACTAGTGA
Lancer          CTTATATTTTATAACTTAAAATTATTATCAAAATGTGACAAAATACAAGAGGACTAGTGA
Gladius         CTTATATTTTATAACTTAAAATTATTATCAAAATGTGACAAAATACAAGAGGACTAGTGA
Kukri           CTTATATTTTATAACTTAAAATTATTATCAAAATGTGACAAAATACAAGAGGACTAGTGA
Landmark        CTTATATTTTATAACTTAAAATTATTATCAAAATGTGACAAAATACAAGAGGACTAGTGA
Mace            CTTATATTTTATAACTTAAAATTATTATCAAAATGTGACAAAATACAAGAGGACTAGTGA
STANLEY         CTTATATTTTATAACTTAAAATTATTATCAAAATGTGACAAAATACAAGAGGACTAGTGA
                ********** *************************************************

Drysdale        ATCAGGACGGAGGCGGTAGGAAAAGAGACAGTGTCACGGTGGAGCATCGGGAGAGAGAGT
RAC875          ATCAGGACGGAGGCGGTAGGAAAAGAGACAGTGTCACGGTGGAGCATCGGGAGAGAGAGT
Norin61         ATCAGGACGGAGGCGGTAGGAAAAGAGACAGTGTCACGGTGGAGCATCGGGAGAGAGAGT
Jagger          ATCAGGACGGAGGCGGTAGGAAAAGAGACAGTGTCACGGTGGAGCATCGGGAGAGAGAGT
Mattis          ATCAGGACGGAGGCGGTAGGAAAAGAGACAGTGTCACGGTGGAGCATCGGGAGAGAGAGT
Julius          ATCAGGACGGAGGCGGTAGGAAAAGAGACAGTGTCACGGTGGAGCATCGGGAGAGAGAGT
Arina           ATCAGGACGGAGGCGGTAGGAAAAGAGACAGTGTCACGGTGGAGCATCGGGAGAGAGAGT
Spelta          ATCAGGACGGAGGCGGTAGGAAAAGAGACAGTGTCACGGTGGAGCATCGGGAGAGAGAGT
Lancer          ATCAGGACGGAGGCGGTAGGAAAAGAGACAGTGTCACGGTGGAGCATCGGGAGAGAGAGT
Gladius         ATCAGGACGGAGGCGGTAGGAAAAGAGACAGTGTCACGGTGGAGCATCGGGAGAGAGAGT
Kukri           ATCAGGACGGAGGCGGTAGGAAAAGAGACAGTGTCACGGTGGAGCATCGGGAGAGAGAGT
Landmark        ATCAGGACGGAGGCGGTAGGAAAAGAGACAGTGTCACGGTGGAGCATCGGGAGAGAGAGT
Mace            ATCAGGACGGAGGCGGTAGGAAAAGAGACAGTGTCACGGTGGAGCATCGGGAGAGAGAGT
STANLEY         ATCAGGACGGAGGCGGTAGGAAAAGAGACAGTGTCACGGTGGAGCATCGGGAGAGAGAGT
                ************************************************************
```

```
                                              -2622
                                                v
Drysdale        CCCAACAGTAAAAAGCCAAACTCACTGAAAAGTAGTGATTAAAAAGAAGATTCACATGCC
RAC875          CCCAACAGTAAAAAGCCAAACTCACTGAAAAGTAGTGATTAAAAAGAAGATTCACATGCC
Norin61         CCCAACAGTAAAAAGCCAAACTCACTGAAAAGTAGTGATTAAAAAGAAGATTCACATGCC
Jagger          CCCAACAGTAAAAAGCCAAACTCACTGAAAAGTAGTGATTAAAAAGAAGATTCACATGCC
Mattis          CCCAACAGTAAAAAGCCAAACTCACTGAAAAGTAGTGATTAAAAAGAAGATTCACATGCC
Julius          CCCAACAGTAAAAAGCCAAACTCACTGAAAAGTAGTGATTAAAAAGAAGATTCACATGCC
Arina           CCCAACAGTAAAAAGCCAAACTCACTGAAAAGTAGTGATTAAAAAGAAGATTCACATGCC
Spelta          CCCAACAGTAAAAAGCCAAACTCACTGAAAAGTAGTGATTAAAAAGAAGATTCACATGCC
Lancer          CCCAACAGTAAAAAGCCAAACTCACTGAAAAGTAATGATTAAAAAGAAGATTCACATGCC
Gladius         CCCAACAGTAAAAAGCCAAACTCACTGAAAAGTAATGATTAAAAAGAAGATTCACATGCC
Kukri           CCCAACAGTAAAAAGCCAAACTCACTGAAAAGTAATGATTAAAAAGAAGATTCACATGCC
Landmark        CCCAACAGTAAAAAGCCAAACTCACTGAAAAGTAATGATTAAAAAGAAGATTCACATGCC
Mace            CCCAACAGTAAAAAGCCAAACTCACTGAAAAGTAATGATTAAAAAGAAGATTCACATGCC
STANLEY         CCCAACAGTAAAAAGCCAAACTCACTGAAAAGTAATGATTAAAAAGAAGATTCACATGCC
                ********************************** *************************

Drysdale        CACTTGCAGTACTACCAAGCAAAAACACAAGGCCAATCGACCAGTAATGTATAATTAAAA
RAC875          CACTTGCAGTACTACCAAGCAAAAACACAAGGCCAATCGACCAGTAATGTATAATTAAAA
Norin61         CACTTGCAGTACTACCAAGCAAAAACACAAGGCCAATCGACCAGTAATGTATAATTAAAA
Jagger          CACTTGCAGTACTACCAAGCAAAAACACAAGGCCAATCGACCAGTAATGTATAATTAAAA
Mattis          CACTTGCAGTACTACCAAGCAAAAACACAAGGCCAATCGACCAGTAATGTATAATTAAAA
Julius          CACTTGCAGTACTACCAAGCAAAAACACAAGGCCAATCGACCAGTAATGTATAATTAAAA
Arina           CACTTGCAGTACTACCAAGCAAAAACACAAGGCCAATCGACCAGTAATGTATAATTAAAA
Spelta          CACTTGCAGTACTACCAAGCAAAAACACAAGGCCAATCGACCAGTAATGTATAATTAAAA
Lancer          CACTTGCAGTACTACCAAGCAAAAACACAAGGCCAATCGACCAGTAATGTATAATTAAAA
Gladius         CACTTGCAGTACTACCAAGCAAAAACACAAGGCCAATCGACCAGTAATGTATAATTAAAA
Kukri           CACTTGCAGTACTACCAAGCAAAAACACAAGGCCAATCGACCAGTAATGTATAATTAAAA
Landmark        CACTTGCAGTACTACCAAGCAAAAACACAAGGCCAATCGACCAGTAATGTATAATTAAAA
Mace            CACTTGCAGTACTACCAAGCAAAAACACAAGGCCAATCGACCAGTAATGTATAATTAAAA
STANLEY         CACTTGCAGTACTACCAAGCAAAAACACAAGGCCAATCGACCAGTAATGTATAATTAAAA
                ************************************************************

Drysdale        ACATTGTCACGGTTCCCCAGTTGAAATTTTTTGGGTCATGGTGTTCAACAGAGTTATTTA
RAC875          ACATTGTCACGGTTCCCCAGTTGAAATTTTTTGGGTCATGGTGTTCAACAGAGTTATTTA
Norin61         ACATTGTCACGGTTCCCCAGTTGAAATTTTTTGGGTCATGGTGTTCAACAGAGTTATTTA
Jagger          ACATTGTCACGGTTCCCCAGTTGAAATTTTTTGGGTCATGGTGTTCAACAGAGTTATTTA
Mattis          ACATTGTCACGGTTCCCCAGTTGAAATTTTTTGGGTCATGGTGTTCAACAGAGTTATTTA
Julius          ACATTGTCACGGTTCCCCAGTTGAAATTTTTTGGGTCATGGTGTTCAACAGAGTTATTTA
Arina           ACATTGTCACGGTTCCCCAGTTGAAATTTTTTGGGTCATGGTGTTCAACAGAGTTATTTA
Spelta          ACATTGTCACGGTTCCCCAGTTGAAATTTTTTGGGTCATGGTGTTCAACAGAGTTATTTA
Lancer          ACATTGTCACGGTTCCCCAGTTGAATTTTTTTGGGTCATGGTGTTCAACAGAGTTATTTA
Gladius         ACATTGTCACGGTTCCCCAGTTGAAATTTTTTGGGTCATGGTGTTCAACAGAGTTATTTA
Kukri           ACATTGTCACGGTTCCCCAGTTGAAATTTTTTGGGTCATGGTGTTCAACAGAGTTATTTA
Landmark        ACATTGTCACGGTTCCCCAGTTGAAATTTTTTGGGTCATGGTGTTCAACAGAGTTATTTA
Mace            ACATTGTCACGGTTCCCCAGTTGAAATTTTTTGGGTCATGGTGTTCAACAGAGTTATTTA
STANLEY         ACATTGTCACGGTTCCCCAGTTGAAATTTTTTGGGTCATGGTGTTCAACAGAGTTATTTA
                ************************* **********************************

Drysdale        ATGTTCTACTAGAGGGCATTAATGTGACATATCGTAGTTTAATCGCGGTGGCGTGGCAAT
RAC875          ATGTTCTACTAGAGGGCATTAATGTGACATATCGTAGTTTAATCGCGGTGGCGTGGCAAT
Norin61         ATGTTCTACTAGAGGGCATTAATGTGACATATCGTAGTTTAATCGCGGTGGCGTGGCAAT
Jagger          ATGTTCTACTAGAGGGCATTAATGTGACATATCGTAGTTTAATCGCGGTGGCGTGGCAAT
Mattis          ATGTTCTACTAGAGGGCATTAATGTGACATATCGTAGTTTAATCGCGGTGGCGTGGCAAT
Julius          ATGTTCTACTAGAGGGCATTAATGTGACATATCGTAGTTTAATCGCGGTGGCGTGGCAAT
Arina           ATGTTCTACTAGAGGGCATTAATGTGACATATCGTAGTTTAATCGCGGTGGCGTGGCAAT
Spelta          ATGTTCTACTAGAGGGCATTAATGTGACATATCGTAGTTTAATCGCGGTGGCGTGGCAAT
Lancer          ATGTTCTACTAGAGGGCATTAATGTGACATATCGTAGTTTAATCGCGGTGGCGTGGCAAT
Gladius         ATGTTCTACTAGAGGGCATTAATGTGACATATCGTAGTTTAATCGCGGTGGCGTGGCAAT
Kukri           ATGTTCTACTAGAGGGCATTAATGTGACATATCGTAGTTTAATCGCGGTGGCGTGGCAAT
Landmark        ATGTTCTACTAGAGGGCATTAATGTGACATATCGTAGTTTAATCGCGGTGGCGTGGCAAT
Mace            ATGTTCTACTAGAGGGCATTAATGTGACATATCGTAGTTTAATCGCGGTGGCGTGGCAAT
STANLEY         ATGTTCTACTAGAGGGCATTAATGTGACATATCGTAGTTTAATCGCGGTGGCGTGGCAAT
                ************************************************************
```

```
Drysdale        GACATTGGCAAGCAAACTAAACTAGCTGAGTGTCCTTGTCCTCTCCACCCACCACTAGTC
RAC875          GACATTGGCAAGCAAACTAAACTAGCTGAGTGTCCTTGTCCTCTCCACCCACCACTAGTC
Norin61         GACATTGGCAAGCAAACTAAACTAGCTGAGTGTCCTTGTCCTCTCCACCCACCACTAGTC
Jagger          GACATTGGCAAGCAAACTAAACTAGCTGAGTGTCCTTGTCCTCTCCACCCACCACTAGTC
Mattis          GACATTGGCAAGCAAACTAAACTAGCTGAGTGTCCTTGTCCTCTCCACCCACCACTAGTC
Julius          GACATTGGCAAGCAAACTAAACTAGCTGAGTGTCCTTGTCCTCTCCACCCACCACTAGTC
Arina           GACATTGGCAAGCAAACTAAACTAGCTGAGTGTCCTTGTCCTCTCCACCCACCACTAGTC
Spelta          GACATTGGCAAGCAAACTAAACTAGCTGAGTGTCCTTGTCCTCTCCACCCACCACTAGTC
Lancer          GACATTGGCAAGCAAACTAAACTAGCTGAGTGTCCTTGTCCTCTCCACCCACCACTAGTC
Gladius         GACATTGGCAAGCAAACTAAACTAGCTGAGTGTCCTTGTCCTCTCCACCCACCACTAGTC
Kukri           GACATTGGCAAGCAAACTAAACTAGCTGAGTGTCCTTGTCCTCTCCACCCACCACTAGTC
Landmark        GACATTGGCAAGCAAACTAAACTAGCTGAGTGTCCTTGTCCTCTCCACCCACCACTAGTC
Mace            GACATTGGCAAGCAAACTAAACTAGCTGAGTGTCCTTGTCCTCTCCACCCACCACTAGTC
STANLEY         GACATTGGCAAGCAAACTAAACTAGCTGAGTGTCCTTGTCCTCTCCACCCACCACTAGTC
                ************************************************************

                                           v
Drysdale        AAGCCTAATTAGCTAAGCTGCGTTGGAAAGGGCTAAAAGTGAGTCTCTCTGGACCTGGAA
RAC875          AAGCCTAATTAGCTAAGCTGCGTTGGAAAGGGCTAAAAGTGAGTCTCTCTGGACCTGGAA
Norin61         AAGCCTAATTAGCTAAGCTGCGTTGGAAAGGGCTAAAAGTGAGTCTCTCTGGACCTGGAA
Jagger          AAGCCTAATTAGCTAAGCTGCGTTGGAAAGGGCTAAAAGTGAGTCTCTCTGGACCTGGAA
Mattis          AAGCCTAATTAGCTAAGCTGCGTTGGAAAGGGCTAAAAGTGAGTCTCTCTGGACCTGGAA
Julius          AAGCCTAATTAGCTAAGCTGCGTTGGAAAGGGCTAAAAGTGAGTCTCTCTGGACCTGGAA
Arina           AAGCCTAATTAGCTAAGCTGCGTTGGAAAGGGCTAAAAGTGAGTCTCTCTGGACCTGGAA
Spelta          AAGCCTAATTAGCTAAGCTGCGTTGGAAAGGGCTAAAAGTGAGTCTCTCTGGACCTGGAA
Lancer          AAGCCTAATTAGCTAAGCTGCGTTGGAGAGGGCTAAAAGTGAGTCTCTCTGGACCTGGAA
Gladius         AAGCCTAATTAGCTAAGCTGCGTTGGAGAGGGCTAAAAGTGAGTCTCTCTGGACCTGGAA
Kukri           AAGCCTAATTAGCTAAGCTGCGTTGGAGAGGGCTAAAAGTGAGTCTCTCTGGACCTGGAA
Landmark        AAGCCTAATTAGCTAAGCTGCGTTGGAGAGGGCTAAAAGTGAGTCTCTCTGGACCTGGAA
Mace            AAGCCTAATTAGCTAAGCTGCGTTGGAGAGGGCTAAAAGTGAGTCTCTCTGGACCTGGAA
STANLEY         AAGCCTAATTAGCTAAGCTGCGTTGGAGAGGGCTAAAAGTGAGTCTCTCTGGACCTGGAA
                *************************** ********************************

                                                                -2241
                                                             v  v
Drysdale        TCCAAACATTCAAAAACGA-TTTGGTGAGAGCATCTCCAGTCGCACCCTCAACACGGCCT
RAC875          TCCAAACATTCAAAAACGATTTTGGTGAGAGCATCTCCAGTCGCACCCTCAACACGGCCT
Norin61         TCCAAACATTCAAAAACGA-TTTGGTGAGAGCATCTCCAGTCGCACCCTTAACACGGCCT
Jagger          TCCAAACATTCAAAAACGA-TTTGGTGAGAGCATCTCCAGTCGCACCCTCAACACGGCCT
Mattis          TCCAAACATTCAAAAACGA-TTTGGTGAGAGCATCTCCAGTCGCACCCTCAACACGGCCT
Julius          TCCAAACATTCAAAAACGA-TTTGGTGAGAGCATCTCCAGTCGCACCCTCAACACGGCCT
Arina           TCCAAACATTCAAAAACGA-TTTGGTGAGAGCATCTCCAGTCGCACCCTCAACACGGCCT
Spelta          TCCAAACATTCAAAAACGA-TTTGGTGAGAGCATCTCCAGTCGCACCCTCAACACGGCCT
Lancer          TCCAAACATTCAAAAACGA-TTTGGTGAGAGCATCTTCAGTCGCATCCCCAACACGGCTT
Gladius         TCCAAACATTCAAAAACGA-TTTGGTGAGAGCATCTCCAGTCGCATCCCCAACACGGCCT
Kukri           TCCAAACATTCAAAAACGA-TTTGGTGAGAGCATCTCCAGTCGCATCCCCAACACGGCCT
Landmark        TCCAAACATTCAAAAACGA-TTTGGTGAGAGCATCTCCAGTCGCATCCCCAACACGGCCT
Mace            TCCAAACATTCAAAAACGA-TTTGGTGAGAGCATCTCCAGTCGCATCCCCAACACGGCCT
STANLEY         TCCAAACATTCAAAAACGA-TTTGGTGAGAGCATCTCCAGTCGCATCCCCAACACGGCCT
                ******************* **************** ******** **  ******** *

                   v
Drysdale        TTCTGGCGATTTTTTCGTGCCGGCGCCTAAAAATGGCCCAGTCGCGTCCCCAGGAGCCCG
RAC875          TTCTGGCGATTTTTTCGTGCCGGCGCCTAAAAATGGCCCAGTCGCGTCCCCAGGAGCCCG
Norin61         TTCTGGCGATTTTTTCGTGCCGGCGCCTAAAAATGGCCCAGTCGCGTCCCCAGGAGCCCG
Jagger          TTCTGGCGATTTTTTCGTGCCGGCGCCTAAAAATGGCCCAGTCGCGTCCCCAGGAGCCCG
Mattis          TTCTGGCGATTTTTTCGTGCCGGCGCCTAAAAATGGCCCAGTCGCGTCCCCAGGAGCCCG
Julius          TTCTGGCGATTTTTTCGTGCCGGCGCCTAAAAATGGCCCAGTCGCGTCCCCAGGAGCCCG
Arina           TTCTGGCGATTTTTTCGTGCCGGCGCCTAAAAATGGCCCAGTCGCGTCCCCAGGAGCCCG
Spelta          TTCTGGCGATTTTTTCGTGCCGGCGCCTAAAAATGGCCCAGTCGCGTCCCCAGGAGCCCG
Lancer          CCCAGGCGATTTTTTCGTGCCGGCGCCTAAAAATGGCCCAGTCGCGTCCCCAGGAGCCCG
Gladius         TCCAGGCGATTTTTTCGTGCCGGCGCCTAAAAATGGCCCAGTCGCGTCCCCAGGAGCCCG
Kukri           TCCAGGCGATTTTTTCGTGCCGGCGCCTAAAAATGGCCCAGTCGCGTCCCCAGGAGCCCG
Landmark        TCCAGGCGATTTTTTCGTGCCGGCGCCTAAAAATGGCCCAGTCGCGTCCCCAGGAGCCCG
Mace            TCCAGGCGATTTTTTCGTGCCGGCGCCTAAAAATGGCCCAGTCGCGTCCCCAGGAGCCCG
STANLEY         TCCAGGCGATTTTTTCGTGCCGGCGCCTAAAAATGGCCCAGTCGCGTCCCCAGGAGCCCG
                  * ********************************************************
```

```
                                                      v     v                       v
Drysdale        TTTTTCACCGGCTTGGGCCGAAATCAGTACAGACGGACCCAGGCCGAACCCGGCGCACTG
RAC875          TTTTTCACCGGCTTGGGCCGAAATCAGTACAGACGGACCCAGGCCGAACCCGGCGCACTG
Norin61         TTTTTCACCGGCTTGGGCCGAAATCAGTACAGACGGACCCAGGCCGAACCCGGCGCACTG
Jagger          TTTTTCACCGGCTTGGGCCGAAATCAGTACAGACGGACCCAGGCCGAACCCGGCGCACTG
Mattis          TTTTTCACCGGCTTGGGCCGAAATCAGTACAGACGGACCCAGGCCGAACCCGGCGCACTG
Julius          TTTTTCACCGGCTTGGGCCGAAATCAGTACAGACGGACCCAGGCCGAACCCGGCGCACTG
Arina           TTTTTCACCGGCTTGGGCCGAAATCAGTACAGACGGACCCAGGCCGAACCCGGCGCACTG
Spelta          TTTTTCACCGGCTTGGGCCGAAATCAGTACAGACGGACCCAGGCCGAACCCGGCGCACTG
Lancer          TTTTTCACCGGTTTGGGCCGAAATCAGTACAGGCGGACCCAGGCCGAACCCGGCGCGCTG
Gladius         TTTTTCACCGGCTTGGGCCGAAATCAATACAGGCGGACCCAGGCCGAACCCGGCGCGCTG
Kukri           TTTTTCACCGGCTTGGGCCGAAATCAATACAGGCGGACCCAGGCCGAACCCGGCGCGCTG
Landmark        TTTTTCACCGGCTTGGGCCGAAATCAATACAGGCGGACCCAGGCCGAACCCGGCGCGCTG
Mace            TTTTTCACCGGCTTGGGCCGAAATCAATACAGGCGGACCCAGGCCGAACCCGGCGCGCTG
STANLEY         TTTTTCACCGGCTTGGGCCGAAATCAATACAGGCGGACCCAGGCCGAACCCGGCGCGCTG
                *********** ************** ***** *********************** ***
                                                     v
Drysdale        GGGGCGCCGGGGCGAGTGGTTTTGGCGCGAAATAGCCGCGGGCCCACCGAGTCTGCGAGA
RAC875          GGGGCGCCGGGGCGAGTGGTTTTGGCGCGAAATAGCCGCGGGCCCACCGAGTCTGCGAGA
Norin61         GGGGCGCCGGGGCGAGTGGTTTTGGCGCGAAATAGCCGCGGGCCCACCGAGTCTGCGAGA
Jagger          GGGGCGCCGGGGCGAGTGGTTTTGGCGCGAAATAGCCGCGGGCCCACCGAGTCTGCGAGA
Mattis          GGGGCGCCGGGGCGAGTGGTTTTGGCGCGAAATAGCCGCGGGCCCACCGAGTCTGCGAGA
Julius          GGGGCGCCGGGGCGAGTGGTTTTGGCGCGAAATAGCCGCGGGCCCACCGAGTCTGCGAGA
Arina           GGGGCGCCGGGGCGAGTGGTTTTGGCGCGAAATAGCCGCGGGCCCACCGAGTCTGCGAGA
Spelta          GGGGCGCCGGGGCGAGTGGTTTTGGCGCGAAATAGCCGCGGGCCCACCGAGTCTGCGAGA
Lancer          GGGGCGCCGGGGCGAGTGGTTTTGGCGCGAAATAGCCGCGGGCCCACCGAGTCTGCGAGA
Gladius         GGGGCGCCGGGGCGAGTGGTTTTGGCGCGAAATAGCCCCGGGCCCACCGAGTCTGCGAGA
Kukri           GGGGCGCCGGGGCGAGTGGTTTTGGCGCGAAATAGCCCCGGGCCCACCGAGTCTGCGAGA
Landmark        GGGGCGCCGGGGCGAGTGGTTTTGGCGCGAAATAGCCCCGGGCCCACCGAGTCTGCGAGA
Mace            GGGGCGCCGGGGCGAGTGGTTTTGGCGCGAAATAGCCCCGGGCCCACCGAGTCTGCGAGA
STANLEY         GGGGCGCCGGGGCGAGTGGTTTTGGCGCGAAATAGCCCCGGGCCCACCGAGTCTGCGAGA
                ************************************* **********************

Drysdale        CGGCCGCTTCGTCGCCCTCATCGCCTTGGTTCCCACGGGAATCAATGCCAAGACTGCCAC
RAC875          CGGCCGCTTCGTCGCCCTCATCGCCTTGGTTCCCACGGGAATCAATGCCAAGACTGCCAC
Norin61         CGGCCGCTTCGTCGCCCTCATCGCCTTGGTTCCCACGGGAATCAATGCCAAGACTGCCAC
Jagger          CGGCCGCTTCGTCGCCCTCATCGCCTTGGTTCCCACGGGAATCAATGCCAAGACTGCCAC
Mattis          CGGCCGCTTCGTCGCCCTCATCGCCTTGGTTCCCACGGGAATCAATGCCAAGACTGCCAC
Julius          CGGCCGCTTCGTCGCCCTCATCGCCTTGGTTCCCACGGGAATCAATGCCAAGACTGCCAC
Arina           CGGCCGCTTCGTCGCCCTCATCGCCTTGGTTCCCACGGGAATCAATGCCAAGACTGCCAC
Spelta          CGGCCGCTTCGTCGCCCTCATCGCCTTGGTTCCCACGGGAATCAATGCCAAGACTGCCAC
Lancer          CGGCCGCTTCGTCGCCCTCATCGCCTTGGTTCCCACGGGAATCAATGCCAAGACTGCCAC
Gladius         CGGCCGCTTCGTCGCCCTCATCGCCTTGGTTCCCACGGGAATCAATGCCAAGACTGCCAC
Kukri           CGGCCGCTTCGTCGCCCTCATCGCCTTGGTTCCCACGGGAATCAATGCCAAGACTGCCAC
Landmark        CGGCCGCTTCGTCGCCCTCATCGCCTTGGTTCCCACGGGAATCAATGCCAAGACTGCCAC
Mace            CGGCCGCTTCGTCGCCCTCATCGCCTTGGTTCCCACGGGAATCAATGCCAAGACTGCCAC
STANLEY         CGGCCGCTTCGTCGCCCTCATCGCCTTGGTTCCCACGGGAATCAATGCCAAGACTGCCAC
                ************************************************************
                       v
Drysdale        GATGCCGCCGCCGGTCAGCCTTGCCATTGATAGCTCATCACGGGCGGCGCGTCACGGGTG
RAC875          GATGCCGCCGCCGGTCAGCCTTGCCATTGATAGCTCATCACGGGCGGCGCGTCACGGGTG
Norin61         GATGCCGCCGCCGGTCAGCCTTGCCATTGATAGCTCATCACGGGCGGCGCGTCACGGGTG
Jagger          GATGCCGCCGCCGGTCAGCCTTGCCATTGATAGCTCATCACGGGCGGCGCGTCACGGGTG
Mattis          GATGCCGCCGCCGGTCAGCCTTGCCATTGATAGCTCATCACGGGCGGCGCGTCACGGGTG
Julius          GATGCCGCCGCCGGTCAGCCTTGCCATTGATAGCTCATCACGGGCGGCGCGTCACGGGTG
Arina           GATGCCGCCGCCGGTCAGCCTTGCCATTGATAGCTCATCACGGGCGGCGCGTCACGGGTG
Spelta          GATGCCGCCGCCGGTCAGCCTTGCCATTGATAGCTCATCACGGGCGGCGCGTCACGGGTG
Lancer          GATGCCGCCGCCGGTCAGCCTTGCCATTGATAGCTCATCACGGGCGGCGCGTCACGGGTG
Gladius         GATGCCGTCGCCGGTCAGCCTTGCCATTGATAGCTCATCACGGGCGGCGCGTCACGGGTG
Kukri           GATGCCGTCGCCGGTCAGCCTTGCCATTGATAGCTCATCACGGGCGGCGCGTCACGGGTG
Landmark        GATGCCGTCGCCGGTCAGCCTTGCCATTGATAGCTCATCACGGGCGGCGCGTCACGGGTG
Mace            GATGCCGTCGCCGGTCAGCCTTGCCATTGATAGCTCATCACGGGCGGCGCGTCACGGGTG
STANLEY         GATGCCGTCGCCGGTCAGCCTTGCCATTGATAGCTCATCACGGGCGGCGCGTCACGGGTG
                ******* ****************************************************
```

```
Drysdale        GCGCGGCGACGCCTCCCCTCCCGCCACGCGTACACACGGGCGCGACTATATAAGCCGGTG
RAC875          GCGCGGCGACGCCTCCCCTCCCGCCACGCGTACACACGGGCGCGACTATATAAGCCGGTG
Norin61         GCGCGGCGACGCCTCCCCTCCCGCCACGCGTACACACGGGCGCGACTATATAAGCCGGTG
Jagger          GCGCGGCGACGCCTCCCCTCCCGCCACGCGTACACACGGGCGCGACTATATAAGCCGGTG
Mattis          GCGCGGCGACGCCTCCCCTCCCGCCACGCGTACACACGGGCGCGACTATATAAGCCGGTG
Julius          GCGCGGCGACGCCTCCCCTCCCGCCACGCGTACACACGGGCGCGACTATATAAGCCGGTG
Arina           GCGCGGCGACGCCTCCCCTCCCGCCACGCGTACACACGGGCGCGACTATATAAGCCGGTG
Spelta          GCGCGGCGACGCCTCCCCTCCCGCCACGCGTACACACGGGCGCGACTATATAAGCCGGTG
Lancer          GCGCGGCAACGCCTCCCCTCCCGCCACGCGTACACACGGGCGCGACTATATAAGCCGGTG
Gladius         GCGCGGCGACGCCTCCCCTCCCGCCACGCGTACACACGGGCGCGACTATATAAGCCGGTG
Kukri           GCGCGGCGACGCCTCCCCTCCCGCCACGCGTACACACGGGCGCGACTATATAAGCCGGTG
Landmark        GCGCGGCGACGCCTCCCCTCCCGCCACGCGTACACACGGGCGCGACTATATAAGCCGGTG
Mace            GCGCGGCGACGCCTCCCCTCCCGCCACGCGTACACACGGGCGCGACTATATAAGCCGGTG
STANLEY         GCGCGGCGACGCCTCCCCTCCCGCCACGCGTACACACGGGCGCGACTATATAAGCCGGTG
                ******* ****************************************************

Drysdale        GCCTCCCTCACCTTTGGCCACACCAGCCCTCGCCGTCCATCGACCGCCGTCGAGCTCTTC
RAC875          GCCTCCCTCACCTTTGGCCACACCAGCCCTCGCCGTCCATCGACCGCCGTCGAGCTCTTC
Norin61         GCCTCCCTCACCTTTGGCCACACCAGCCCTCGCCGTCCATCGACCGCCGTCGAGCTCTTC
Jagger          GCCTCCCTCACCTTTGGCCACACCAGCCCTCGCCGTCCATCGACCGCCGTCGAGCTCTTC
Mattis          GCCTCCCTCACCTTTGGCCACACCAGCCCTCGCCGTCCATCGACCGCCGTCGAGCTCTTC
Julius          GCCTCCCTCACCTTTGGCCACACCAGCCCTCGCCGTCCATCGACCGCCGTCGAGCTCTTC
Arina           GCCTCCCTCACCTTTGGCCACACCAGCCCTCGCCGTCCATCGACCGCCGTCGAGCTCTTC
Spelta          GCCTCCCTCACCTTTGGCCACACCAGCCCTCGCCGTCCATCGACCGCCGTCGAGCTCTTC
Lancer          GCCTCCCTCACCTTTGGCCACACCAGCCCTCGCCGTCCATCGATCGCCGTCGAGCTCTTC
Gladius         GCCTCCCTCACCTTTGGCCACACCAGCCCTCGCCGTCCATCGACCGCCGTCGAGCTCTTC
Kukri           GCCTCCCTCACCTTTGGCCACACCAGCCCTCGCCGTCCATCGACCGCCGTCGAGCTCTTC
Landmark        GCCTCCCTCACCTTTGGCCACACCAGCCCTCGCCGTCCATCGACCGCCGTCGAGCTCTTC
Mace            GCCTCCCTCACCTTTGGCCACACCAGCCCTCGCCGTCCATCGACCGCCGTCGAGCTCTTC
STANLEY         GCCTCCCTCACCTTTGGCCACACCAGCCCTCGCCGTCCATCGACCGCCGTCGAGCTCTTC
                ******************************************* ****************

Drysdale        CTCTCCCGTGCGCAGCCGCGACGCTCCTTCCCTCCCTCTCCCTCACTCCCAAGCCCGGTG
RAC875          CTCTCCCGTGCGCAGCCGCGACGCTCCTTCCCTCCCTCTCCCTCACTCCCAAGCCCGGTG
Norin61         CTCTCCCGTGCGCAGCCGCGACGCTCCTTCCCTCCCTCTCCCTCACTCCCAAGCCCGGTG
Jagger          CTCTCCCGTGCGCAGCCGCGACGCTCCTTCCCTCCCTCTCCCTCACTCCCAAGCCCGGTG
Mattis          CTCTCCCGTGCGCAGCCGCGACGCTCCTTCCCTCCCTCTCCCTCACTCCCAAGCCCGGTG
Julius          CTCTCCCGTGCGCAGCCGCGACGCTCCTTCCCTCCCTCTCCCTCACTCCCAAGCCCGGTG
Arina           CTCTCCCGTGCGCAGCCGCGACGCTCCTTCCCTCCCTCTCCCTCACTCCCAAGCCCGGTG
Spelta          CTCTCCCGTGCGCAGCCGCGACGCTCCTTCCCTCCCTCTCCCTCACTCCCAAGCCCGGTG
Lancer          CTCTCCC-TGCGCAGCCGCGACGCTTCTTCCCTCCCTCTCCCTCACTCCCAAGCCCGATG
Gladius         CTCTCCCGTGCGCAGCCGCGACGCTTCTTCCCTCCCTCTCCCTCACTCCCAAGCCCGGTG
Kukri           CTCTCCCGTGCGCAGCCGCGACGCTTCTTCCCTCCCTCTCCCTCACTCCCAAGCCCGGTG
Landmark        CTCTCCCGTGCGCAGCCGCGACGCTTCTTCCCTCCCTCTCCCTCACTCCCAAGCCCGGTG
Mace            CTCTCCCGTGCGCAGCCGCGACGCTTCTTCCCTCCCTCTCCCTCACTCCCAAGCCCGGTG
STANLEY         CTCTCCCGTGCGCAGCCGCGACGCTTCTTCCCTCCCTCTCCCTCACTCCCAAGCCCGGTG
                ******* ***************** ******************************* **

Drysdale        GCCGCGCGTTTCCCCGGCGACGGCCAACGGCTTCGGTCACCGCTCGCTTCACGAGTGGGA
RAC875          GCCGCGCGTTTCCCCGGCGACGGCCAACGGCTTCGGTCACCGCTCGCTTCACGAGTGGGA
Norin61         GCCGCGCGTTTCCCCGGCGACGGCCAACGGCTTCGGTCACCGCTCGCTTCACGAGTGGGA
Jagger          GCCGCGCGTTTCCCCGGCGACGGCCAACGGCTTCGGTCACCGCTCGCTTCACGAGTGGGA
Mattis          GCCGCGCGTTTCCCCGGCGACGGCCAACGGCTTCGGTCACCGCTCGCTTCACGAGTGGGA
Julius          GCCGCGCGTTTCCCCGGCGACGGCCAACGGCTTCGGTCACCGCTCGCTTCACGAGTGGGA
Arina           GCCGCGCGTTTCCCCGGCGACGGCCAACGGCTTCGGTCACCGCTCGCTTCACGAGTGGGA
Spelta          GCCGCGCGTTTCCCCGGCGACGGCCAACGGCTTCGGTCACCGCTCGCTTCACGAGTGGGA
Lancer          GCCGCGCGTTTCCCCGGCGACGGCCAACGGCTTCGGTCACCGCTCGCTTCACGAGTGGGA
Gladius         GCCGCGCGTTTCCCCGGCGACGGCCAACGGCTTCGGTCACCGCTCGCTTCACGAGTGGGA
Kukri           GCCGCGCGTTTCCCCGGCGACGGCCAACGGCTTCGGTCACCGCTCGCTTCACGAGTGGGA
Landmark        GCCGCGCGTTTCCCCGGCGACGGCCAACGGCTTCGGTCACCGCTCGCTTCACGAGTGGGA
Mace            GCCGCGCGTTTCCCCGGCGACGGCCAACGGCTTCGGTCACCGCTCGCTTCACGAGTGGGA
STANLEY         GCCGCGCGTTTCCCCGGCGACGGCCAACGGCTTCGGTCACCGCTCGCTTCACGAGTGGGA
                ************************************************************
```

```
Drysdale        GGCGTGGCTCCTGTTCGAGGCGAACATCCCGGCGCCACCGGACATGCGCGCATGAACGAT
RAC875          GGCGTGGCTCCTGTTCGAGGCGAACATCCCGGCGCCACCGGACATGCGCGCATGAACGAT
Norin61         GGCGTGGCTCCTGTTCGAGGCGAACATCCCGGCGCCACCGGACATGCGCGCATGAACGAT
Jagger          GGCGTGGCTCCTGTTCGAGGCGAACATCCCGGCGCCACCGGACATGCGCGCATGAACGAT
Mattis          GGCGTGGCTCCTGTTCGAGGCGAACATCCCGGCGCCACCGGACATGCGCGCATGAACGAT
Julius          GGCGTGGCTCCTGTTCGAGGCGAACATCCCGGCGCCACCGGACATGCGCGCATGAACGAT
Arina           GGCGTGGCTCCTGTTCGAGGCGAACATCCCGGCGCCACCGGACATGCGCGCATGAACGAT
Spelta          GGCGTGGCTCCTGTTCGAGGCGAACATCCCGGCGCCACCGGACATGCGCGCATGAACGAT
Lancer          GGCGTGGCTCCTGTTCGAGGCGAACATCCCGGCGCCACCGGACATGCGCGCATGAACGAT
Gladius         GGCGTGGCTCCTGTTCGAGGCGAACATCCCGGCGCCACCGGACATGCGCGCATGAACGAT
Kukri           GG-GTGGCTCCTGTTCGAGGCGAACATCCCGGCGCCACCGGACATGCGCGCATGAACGAT
Landmark        GGCGTGGCTCCTGTTCGAGGCGAACATCCCGGCGCCACCGGACATGCGCGCATGAACGAT
Mace            GGCGTGGCTCCTGTTCGAGGCGAACATCCCGGCGCCACCGGACATGCGCGCATGAACGAT
STANLEY         GGCGTGGCTCCTGTTCGAGGCGAACATCCCGGCGCCACCGGACATGCGCGCATGAACGAT
                ** *********************************************************

Drysdale        GGGGTGGAGACTCGGCAACGGGGGAGTGCCCATT-CCCCCGGTGCCCGACGTCGAGGCGT
RAC875          GGGGTGGAGACTCGGCAACGGGGGAGTGCCCATT-CCCCCGGTGCCCGACGTCGAGGCGT
Norin61         GGGGTGGAGACTCGGCAACGGGGGAGTGCCCATT-CCCCCGGTGCCCGACGTCGAGGCGT
Jagger          GGGGTGGAGACTCGGCAACGGGGGAGTGCCCATT-CCCCCGGTGCCCGACGTCGAGGCGT
Mattis          GGGGTGGAGACTCGGCAACGGGGGAGTGCCCATT-CCCCCGGTGCCCGACGTCGAGGCGT
Julius          GGGGTGGAGACTCGGCAACGGGGGAGTGCCCATT-CCCCCGGTGCCCGACGTCGAGGCGT
Arina           GGGGTGGAGACTCGGCAACGGGGGAGTGCCCATT-CCCCCGGTGCCCGACGTCGAGGCGT
Spelta          GGGGTGGAGACTCGGCAACGGGGGAGTGCCCATT-CCCCCGGTGCCCGACGTCGAGGCGT
Lancer          GGGGTGGAGACTCGGCAACGGGGGAGTGCCCATT-CCCCCGGTGCCCGACGTCGAGGCGT
Gladius         GGGGTGGAGACTCGGCAACGGGGGAGTGCCCATT-CCCCCGGTGCCCGACGTCGAGGCGT
Kukri           GGGGTGGAGACTCGGCAACGGGGGAGTGCCCATTCCCCCCGGTGCCCGACGTCGAGGCGT
Landmark        GGGGTGGAGACTCGGCAACGGGGGAGTGCCCATT-CCCCCGGTGCCCGACGTCGAGGCGT
Mace            GGGGTGGAGACTCGGCAACGGGGGAGTGCCCATT-CCCCCGGTGCCCGACGTCGAGGCGT
STANLEY         GGGGTGGAGACTCGGCAACGGGGGAGTGCCCATT-CCCCCGGTGCCCGACGTCGAGGCGT
                ********************************** *************************

                                                          vv
Drysdale        GCCCCGCCTTCTTCGCCGCCGAGGTCGACCGCGTGCAAGCCT-TCTCACAGCGGAGCAAC
RAC875          GCCCCGCCTTCTTCGCCGCCGAGGTCGACCGCGTGCAAGCCT-TCTCACAGCGGAGCAAC
Norin61         GCCCCGCCTTCTTCGCCGCCGAGGTCGACCGCGTGCAAGCCT-TCTCACAGCGGAGCAAC
Jagger          GCCCCGCCTTCTTCGCCGCCGAGGTCGACCGCGTGCAAGCCT-TCTCACAGCGGAGCAAC
Mattis          GCCCCGCCTTCTTCGCCGCCGAGGTCGACCGCGTGCAAGCCT-TCTCACAGCGGAGCAAC
Julius          GCCCCGCCTTCTTCGCCGCCGAGGTCGACCGCGTGCAAGCCT-TCTCACAGCGGAGCAAC
Arina           GCCCCGCCTTCTTCGCCGCCGAGGTCGACCGCGTGCAAGCCT-TCTCACAGCGGAGCAAC
Spelta          GCCCCGCCTTCTTCGCCGCCGAGGTCGACCGCGTGCAAGCCT-TCTCACAGCGGAGCAAC
Lancer          GCCCCGCCTTCTTCGCCGCCGAGGTCGACCGCGTGCAAGCCT-TCTCACAGCGGAGCAAC
Gladius         GCCCCGCCTTCTTCGCCGCCGAGGTCGACCGCGTGCAAGCCTCCCTCACAGCGGAGCAAC
Kukri           GCCCCGCCTTCTTCGCCGCCGAGGTCGACCGCGTGCAAGCCTCCCTCACAGCGGAGCAAC
Landmark        GCCCCGCCTTCTTCGCCGCCGAGGTCGACCGCGTGCAAGCCTCCCTCACAGCGGAGCAAC
Mace            GCCCCGCCTTCTTCGCCGCCGAGGTCGACCGCGTGCAAGCCTCCCTCACAGCGGAGCAAC
STANLEY         GCCCCGCCTTCTTCGCCGCCGAGGTCGACCGCGTGCAAGCCTCCCTCACAGCGGAGCAAC
                ******************************************  ****************

                                                                         v
Drysdale        TCGCCCCCCACCAGTACGCCGTCGAAAACCACGCGGCGTGGGCGGCGTACTTCCAGCGCC
RAC875          TCGCCCCCCACCAGTACGCCGTCGAAAACCACGCGGCGTGGGCGGCGTACTTCCAGCGCC
Norin61         TCGCCCCCCACCAGTACGCCGTCGAAAACCACGCGGCGTGGGCGGCGTACTTCCAGCGCC
Jagger          TCGCCCCCCACCAGTACGCCGTCGAAAACCACGCGGCGTGGGCGGCGTACTTCCAGCGCC
Mattis          TCGCCCCCCACCAGTACGCCGTCGAAAACCACGCGGCGTGGGCGGCGTACTTCCAGCGCC
Julius          TCGCCCCCCACCAGTACGCCGTCGAAAACCACGCGGCGTGGGCGGCGTACTTCCAGCGCC
Arina           TCGCCCCCCACCAGTACGCCGTCGAAAACCACGCGGCGTGGGCGGCGTACTTCCAGCGCC
Spelta          TCGCCCCCCACCAGTACGCCGTCGAAAACCACGCGGCGTGGGCGGCGTACTTCCAGCGCC
Lancer          TCGCCCCCCACCAGTACGCCGTCGAAAACCACGCGGCGTGGGCGGCGTACTTCCAGCGCC
Gladius         TCGCCCCCCACCAGTACGCCGTCGAAAACCACGCGGCGTGGGCGGCGTACTTCCAGCACC
Kukri           TCGCCCCCCACCAGTACGCCGTCGAAAACCACGCGGCGTGGGCGGCGTACTTCCAGCACC
Landmark        TCGCCCCCCACCAGTACGCCGTCGAAAACCACGCGGCGTGGGCGGCGTACTTCCAGCACC
Mace            TCGCCCCCCACCAGTACGCCGTCGAAAACCACGCGGCGTGGGCGGCGTACTTCCAGCACC
STANLEY         TCGCCCCCCACCAGTACGCCGTCGAAAACCACGCGGCGTGGGCGGCGTACTTCCAGCACC
                ********************************************************* **
```

```
                                                                          v
Drysdale        GCCAGGAGCAAAGACTGGCGTCCACCAACGGGGCGCCGATGGTGGGCAGCGTCAAGAATA
RAC875          GCCAGGAGCAAAGACTGGCGTCCACCAACGGGGCGCCGATGGTGG-CAGCGTCAAGAATA
Norin61         GCCAGGAGCAAAGACTGGCGTCCACCAACGGGGCGCCGATGGTGGGCAGCGTCAAGAATA
Jagger          GCCAGGAGCAAAGACTGGCGTCCACCAACGGGGCGCCGATGGTGGGCAGCGTCAAGAATA
Mattis          GCCAGGAGCAAAGACTGGCGTCCACCAACGGGGCGCCGATGGTGGGCAGCGTCAAGAATA
Julius          GCCAGGAGCAAAGACTGGCGTCCACCAACGGGGCGCCGATGGTGGGCAGCGTCAAGAATA
Arina           GCCAGGAGCAAAGACTGGCGTCCACCAACGGGGCGCCGATGGTGGGCAGCGTCAAGAATA
Spelta          GCCAGGAGCAAAGACTGGCGTCCACCAACGGGGCGCCGATGGTGGGCAGCGTCAAGAATA
Lancer          GCCAGGAGCAAAGACTGGCGTCCACCAACGGGGCGCCGATGGTGGCCAGCGTCAAGAACA
Gladius         GCCAGGAGCAAAGACTGGCGTCCACCAACGGGGCGCCGATGGTGGGCAGCGTCAAGAACA
Kukri           GCCAGGAGCAAAGACTGGCGTCCACCAACGGGGCGCCGATGGTGGGCAGCGTCAAGAACA
Landmark        GCCAGGAGCAAAGACTGGCGTCCACCAACGGGGCGCCGATGGTGGGCAGCGTCAAGAACA
Mace            GCCAGGAGCAAAGACTGGCGTCCACCAACGGGGCGCCGATGGTGGGCAGCGTCAAGAACA
STANLEY         GCCAGGAGCAAAGACTGGCGTCCACCAACGGGGCGCCGATGGTGGGCAGCGTCAAGAACA
                ********************************************* ************ *
                                             v
Drysdale        GTGAGGGGCACCGCGTGTGGTGGGGCGCCCCCGAATATGCTCGACGGCGTGCTGTCGTAC
RAC875          GTGAGGGGCACCGCGTGTGGTGGGGCGCCCCCGAATATGCTCGACGGCGTGCTGTCGTAC
Norin61         GTGAGGGGCACCGCGTGTGGTGGGGCGCCCCCGAATATGCTCGACGGCGTGCTGTCGTAC
Jagger          GTGAGGGGCACCGCGTGTGGTGGGGCGCCCCCGAATATGCTCGACGGCGTGCTGTCGTAC
Mattis          GTGAGGGGCACCGCGTGTGGTGGGGCGCCCCCGAATATGCTCGACGGCGTGCTGTCGTAC
Julius          GTGAGGGGCACCGCGTGTGGTGGGGCGCCCCCGAATATGCTCGACGGCGTGCTGTCGTAC
Arina           GTGAGGGGCACCGCGTGTGGTGGGGCGCCCCCGAATATGCTCGACGGCGTGCTGTCGTAC
Spelta          GTGAGGGGCACCGCGTGTGGTGGGGCGCCCCCGAATATGCTCGACGGCGTGCTGTCGTAC
Lancer          GTGAGGGGCACCGCGTGTGGTGGGGCGCCCCCGAATATGCTCGACGGCGTGCTGTCGTAC
Gladius         GTGAGGGGCACCGCGTGTGGTGGGGCGCCACCGAATATGCTCGACGGCGTGCTGTCGTAC
Kukri           GTGAGGGGCACCGCGTGTGGTGGGGCGCCACCGAATATGCTCGACGGCGTGCTGTCGTAC
Landmark        GTGAGGGGCACCGCGTGTGGTGGGGCGCCACCGAATATGCTCGACGGCGTGCTGTCGTAC
Mace            GTGAGGGGCACCGCGTGTGGTGGGGCGCCACCGAATATGCTCGACGGCGTGCTGTCGTAC
STANLEY         GTGAGGGGCACCGCGTGTGGTGGGGCGCCACCGAATATGCTCGACGGCGTGCTGTCGTAC
                ***************************** ******************************

Drysdale        CTCGAGGGCGGTAACGAACCGTCGCTGGCATACCGTCCCATGGCGGCCGCCCCGGTCCAC
RAC875          CTCGAGGGCGGTAACGAACCGTCGCTGGCATACCGTCCCATGGCGGCCGCCCCGGTCCAC
Norin61         CTCGAGGGCGGTAACGAACCGTCGCTGGCATACCGTCCCATGGCGGCCGCCCCGGTCCAC
Jagger          CTCGAGGGCGGTAACGAACCGTCGCTGGCATACCGTCCCATGGCGGCCGCCCCGGTCCAC
Mattis          CTCGAGGGCGGTAACGAACCGTCGCTGGCATACCGTCCCATGGCGGCCGCCCCGGTCCAC
Julius          CTCGAGGGCGGTAACGAACCGTCGCTGGCATACCGTCCCATGGCGGCCGCCCCGGTCCAC
Arina           CTCGAGGGCGGTAACGAACCGTCGCTGGCATACCGTCCCATGGCGGCCGCCCCGGTCCAC
Spelta          CTCGAGGGCGGTAACGAACCGTCGCTGGCATACCGTCCCATGGCGGCCGCCCCGGTCCAC
Lancer          CTCAAGGGCGGTAACGAACCATCGCTGGCATACCGTCCCATGGCGGCCGCCCCGGTCCAC
Gladius         CTCGAGGGCGGTAACGAACCGTCGCTGGCATACCGTCCCATGGCGGCCGCCCCGGTCCAC
Kukri           CTCGAGGGCGGTAACGAACCGTCGCTGGCATACCGTCCCATGGCGGCCGCCCCGGTCCAC
Landmark        CTCGAGGGCGGTAACGAACCGTCGCTGGCATACCGTCCCATGGCGGCCGCCCCGGTACAC
Mace            CTCGAGGGCGGTAACGAACCGTCGCTGGCATACCGTCCCATGGCGGCCGCCCCGGTCCAC
STANLEY         CTCGAGGGCGGTAACGAACCGTCGCTGGCATACCGTCCCATGGCGGCCGCCCCGGTCCAC
                *** **************** *********************************** ***
                           v                        v
Drysdale        CGCCGGCACGCCGGGCAATGGATGCCCAGGAGGTTCGGTTCCTCCTCGACTTCCTCCTCG
RAC875          CGCCGGCACGCCGGGCAATGGATGCCCAGGAGGTTCGGTTCCTCCTCGACTTCCTCCTCG
Norin61         CGCCGGCACGCCGGGCAATGGATGCCCAGGAGGTTCGGTTCCTCCTCGACTTCCTCCTCG
Jagger          CGCCGGCACGCCGGGCAATGGATGCCCAGGAGGTTCGGTTCCTCCTCGACTTCCTCCTCG
Mattis          CGCCGGCACGCCGGGCAATGGATGCCCAGGAGGTTCGGTTCCTCCTCGACTTCCTCCTCG
Julius          CGCCGGCACGCCGGGCAATGGATGCCCAGGAGGTTCGGTTCCTCCTCGACTTCCTCCTCG
Arina           CGCCGGCACGCCGGGCAATGGATGCCCAGGAGGTTCGGTTCCTCCTCGACTTCCTCCTCG
Spelta          CGCCGGCACGCCGGGCAATGGATGCCCAGGAGGTTCGGTTCCTCCTCGACTTCCTCCTCG
Lancer          CGCCGGCACACCGGGCAATGGATGCCCAGGAGGTTCGGTTCCTCCTCGACTTCCTCCTCG
Gladius         CGCCGGCACGCGGGGCAATGGATGCCCAGGAGGTTCCGTTCCTCCTCGACTTCCTCCTCG
Kukri           CGCCGGCACGCGGGGCAATGGATGCCCAGGAGGTTCCGTTCCTCCTCGACTTCCTCCTCG
Landmark        CGCCGGCACGCGGGGCAATGGATGCCCAGGAGGTTCCGTTCCTCCTCGACTTCCTCCTCG
Mace            CGCCGGCACGCGGGGCAATGGATGCCCAGGAGGTTCCGTTCCTCCTCGACTTCCTCCTCG
STANLEY         CGCCGGCACGCGGGGCAATGGATGCCCAGGAGGTTCCGTTCCTCCTCGACTTCCTCCTCG
                ********* * ************************ ***********************
```

```
Drysdale        CACTCCTCCTCCCGTTCTTCTTCCCACTCCTCCGGCTCGACGACGTTGCTCGGCGTCAAG
RAC875          CACTCCTCCTCCCGTTCTTCTTCCCACTCCTCCGGCTCGACGACGTTGCTCGGCGTCAAG
Norin61         CACTCCTCCTCCCGTTCTTCTTCCCACTCCTCCGGCTCGACGACGTTGCTCGGCGTCAAG
Jagger          CACTCCTCCTCCCGTTCTTCTTCCCACTCCTCCGGCTCGACGACGTTGCTCGGCGTCAAG
Mattis          CACTCCTCCTCCCGTTCTTCTTCCCACTCCTCCGGCTCGACGACGTTGCTCGGCGTCAAG
Julius          CACTCCTCCTCCCGTTCTTCTTCCCACTCCTCCGGCTCGACGACGTTGCTCGGCGTCAAG
Arina           CACTCCTCCTCCCGTTCTTCTTCCCACTCCTCCGGCTCGACGACGTTGCTCGGCGTCAAG
Spelta          CACTCCTCCTCCCGTTCTTCTTCCCACTCCTCCGGCTCGACGACGTTGCTCGGCGTCAAG
Lancer          CACTCCTCCTCCCGTTCTTCTTCCCACTCCTCCGGCTCGACGATGTTGCTCGGCGTCAAG
Gladius         CACTCCTCCTCCCGTTCTTCTTCCCACTCCTCCGGCTCGACGACGTTGCTCGGCGTCAAG
Kukri           CACTCCTCCTCCCGTTCTTCTTCCCACTCCTCCGGCTCGACGACGTTGCTCGGCGTCAAG
Landmark        CACTCCTCCTCCCGTTCTTCTTCCCACTCCTCCGGCTCGACGACGTTGCTCGGCGTCAAG
Mace            CACTCCTCCTCCCGTTCTTCTTCCCACTCCTCCGGCTCGACGACGTTGCTCGGCGTCAAG
STANLEY         CACTCCTCCTCCCGTTCTTCTTCCCACTCCTCCGGCTCGACGACGTTGCTCGGCGTCAAG
                ******************************************* ****************

                                           v
Drysdale        GCCGAGCCCGCAGCGGAGACGCTGCTCGGCCGGCGCACTCGCAGCGTCGGCATCATCATA
RAC875          GCCGAGCCCGCAGCGGAGACGCTGCTCGGCCGGCGCACTCGCAGCGTCGGCATCATCATA
Norin61         GCCGAGCCCGCAGCGGAGACGCTGCTCGGCCGGCGCACTCGCAGCGTCGGCATCATCATA
Jagger          GCCGAGCCCGCAGCGGAGACGCTGCTCGGCCGGCGCACTCGCAGCGTCGGCATCATCATA
Mattis          GCCGAGCCCGCAGCGGAGACGCTGCTCGGCCGGCGCACTCGCAGCGTCGGCATCATCATA
Julius          GCCGAGCCCGCAGCGGAGACGCTGCTCGGCCGGCGCACTCGCAGCGTCGGCATCATCATA
Arina           GCCGAGCCCGCAGCGGAGACGCTGCTCGGCCGGCGCACTCGCAGCGTCGGCATCATCATA
Spelta          GCCGAGCCCGCAGCGGAGACGCTGCTCGGCCGGCGCACTCGCAGCGTCGGCATCATCATA
Lancer          GCCGAGCCCGCAGCGGAGACGCTGCTCGGCCGGCGCACTCGCAGCGTCGGCATCATCATA
Gladius         GCCGAGCCCGCAGCGGAGACGCTGCTCAGCCGGCGCACTCGCAGCGTCGGCATCATCATA
Kukri           GCCGAGCCCGCAGCGGAGACGCTGCTCAGCCGGCGCACTCGCAGCGTCGGCATCATCATA
Landmark        GCCGAGCCCGCAGCGGAGACGCTGCTCAGCCGGCGCACTCGCAGCGTCGGCATCATCATA
Mace            GCCGAGCCCGCAGCGGAGACGCTGCTCAGCCGGCGCACTCGCAGCGTCGGCATCATCATA
STANLEY         GCCGAGCCCGCAGCGGAGACGCTGCTCAGCCGGCGCACTCGCAGCGTCGGCATCATCATA
                *************************** ********************************

                                                                           v
Drysdale        AACGAGGGCGGCCGGCGCTCCTCCTCATCGGCTCCTCCGCGCTTCGTCAAACCAAAGACG
RAC875          AACGAGGGCGGCCGGCGCTCCTCCTCATCGGCTCCTCCGCGCTTCGTCAAACCAAAGACG
Norin61         AACGAGGGCGGCCGGCGCTCCTCCTCATCGGCTCCTCCGCGCTTCGTCAAACCAAAGACG
Jagger          AACGAGGGCGGCCGGCGCTCCTCCTCATCGGCTCCTCCGCGCTTCGTCAAACCAAAGACG
Mattis          AACGAGGGCGGCCGGCGCTCCTCCTCATCGGCTCCTCCGCGCTTCGTCAAACCAAAGACG
Julius          AACGAGGGCGGCCGGCGCTCCTCCTCATCGGCTCCTCCGCGCTTCGTCAAACCAAAGACG
Arina           AACGAGGGCGGCCGGCGCTCCTCCTCATCGGCTCCTCCGCGCTTCGTCAAACCAAAGACG
Spelta          AACGAGGGCGGCCGGCGCTCCTCCTCATCGGCTCCTCCGCGCTTCGTCAAACCAAAGACG
Lancer          AACGAGGGCGGCCGGCGCTCCTCCTCATCGGCTCCTCCGCGCTTCGTCAAACCAAAGAC-
Gladius         AACGAGGGCGGCCGGCGCTCCTCCTCATCGGCTCCTCCGCGCTTCGTCAAACCAAAGAC-
Kukri           AACGAGGGCGGCCGGCGCTCCTCCTCATCGGCTCCTCCGCGCTTCGTCAAACCAAAGAC-
Landmark        AACGAGGGCGGCCGGCGCTCCTCCTCATCGGCTCCTCCGCGCTTCGTCAAACCAAAGAC-
Mace            AACGAGGGCGGCCGGCGCTCCTCCTCATCGGCTCCTCCGCGCTTCGTCAAACCAAAGAC-
STANLEY         AACGAGGGCGGCCGGCGCTCCTCCTCATCGGCTCCTCCGCGCTTCGTCAAACCAAAGAC-
                ***********************************************************

Drysdale        GGAACCGAGGCTCGCGGCCGTGAAAACGGAGCCGGGGCTCCCCGCCGTGAAGACAGAGCA
RAC875          GGAACCGAGGCTCGCGGCCGTGAAAACGGAGCCGGGGCTCCCCGCCGTGAAGACAGAGCA
Norin61         GGAACCGAGGCTCGCGGCCGTGAAAACGGAGCCGGGGCTCCCCGCCGTGAAGACAGAGCA
Jagger          GGAACCGAGGCTCGCGGCCGTGAAAACGGAGCCGGGGCTCCCCGCCGTGAAGACAGAGCA
Mattis          GGAACCGAGGCTCGCGGCCGTGAAAACGGAGCCGGGGCTCCCCGCCGTGAAGACAGAGCA
Julius          GGAACCGAGGCTCGCGGCCGTGAAAACGGAGCCGGGGCTCCCCGCCGTGAAGACAGAGCA
Arina           GGAACCGAGGCTCGCGGCCGTGAAAACGGAGCCGGGGCTCCCCGCCGTGAAGACAGAGCA
Spelta          GGAACCGAGGCTCGCGGCCGTGAAAACGGAGCCGGGGCTCCCCGCCGTGAAGACAGAGCA
Lancer          GGAACCGAGGCTCGCGGCCGTGAAAACGGAGCCGGGGCTCCCCGCCGTGAAGACAGAGCA
Gladius         GGAACCGAGGCTCGCGGCCGTGAAAACGGAGCCGGGGCTCCCCGCCGTGAAGACAGAGCA
Kukri           GGAACCGAGGCTCGCGGCCGTGAAAACGGAGCCGGGGCTCCCCGCCGTGAAGACAGAGCA
Landmark        GGAACCGAGGCTCGCGGCCGTGAAAACGGAGCCGGGGCTCCCCGCCGTGAAGACAGAGCA
Mace            GGAACCGAGGCTCGCGGCCGTGAAAACGGAGCCGGGGCTCCCCGCCGTGAAGACAGAGCA
STANLEY         GGAACCGAGGCTCGCGGCCGTGAAAACGGAGCCGGGGCTCCCCGCCGTGAAGACAGAGCA
                ************************************************************
```

```
                                                              v
Drysdale        CGGCGACGTGGAGCTCGATGAGGAGGCGACCCTAAAATGGGCGCGCACCGACTGGCTCAA
RAC875          CGGCGACGTGGAGCTCGATGAGGAGGCGACCCTAAAATGGGCGCGCACCGACTGGCTCAA
Norin61         CGGCGACGTGGAGCTCGATGAGGAGGCGACCCTAAAATGGGCGCGCACCGACTGGCTCAA
Jagger          CGGCGACGTGGAGCTCGATGAGGAGGCGACCCTAAAATGGGCGCGCACCGACTGGCTCAA
Mattis          CGGCGACGTGGAGCTCGATGAGGAGGCGACCCTAAAATGGGCGCGCACCGACTGGCTCAA
Julius          CGGCGACGTGGAGCTCGATGAGGAGGCGACCCTAAAATGGGCGCGCACCGACTGGCTCAA
Arina           CGGCGACGTGGAGCTCGATGAGGAGGCGACCCTAAAATGGGCGCGCACCGACTGGCTCAA
Spelta          CGGCGACGTGGAGCTCGATGAGGAGGCGACCCTAAAATGGGCGCGCACCGACTGGCTCAA
Lancer          CGGCGACGTGGAGCTCGATGAGGAGGCGACCCTAAAATGGGCGCGCGCCGACTGGCTCAA
Gladius         CGGCGACGTGGAGCTCGATGAGGAGGCGACCCTAAAATGGGCGCGCGCCGACTGGCTCAA
Kukri           CGGCGACGTGGAGCTCGATGAGGAGGCGACCCTAAAATGGGCGCGCGCCGACTGGCTCAA
Landmark        CGGCGACGTGGAGCTCGATGAGGAGGCGACCCTAAAATGGGCGCGCGCCGACTGGCTCAA
Mace            CGGCGACGTGGAGCTCGATGAGGAGGCGACCCTAAAATGGGCGCGCGCCGACTGGCTCAA
STANLEY         CGGCGACGTGGAGCTCGATGAGGAGGCGACCCTAAAATGGGCGCGCGCCGACTGGCTCAA
                ********************************************** *************

Drysdale        GATGGAGTGGGAGCGCCAGTGCGCCGCCCTGCAGCACTACGAACAGCGCCGCCAGGGCCG
RAC875          GATGGAGTGGGAGCGCCAGTGCGCCGCCCTGCAGCACTACGAACAGCGCCGCCAGGGCCG
Norin61         GATGGAGTGGGAGCGCCAGTGCGCCGCCCTGCAGCACTACGAACAGCGCCGCCAGGGCCG
Jagger          GATGGAGTGGGAGCGCCAGTGCGCCGCCCTGCAGCACTACGAACAGCGCCGCCAGGGCCG
Mattis          GATGGAGTGGGAGCGCCAGTGCGCCGCCCTGCAGCACTACGAACAGCGCCGCCAGGGCCG
Julius          GATGGAGTGGGAGCGCCAGTGCGCCGCCCTGCAGCACTACGAACAGCGCCGCCAGGGCCG
Arina           GATGGAGTGGGAGCGCCAGTGCGCCGCCCTGCAGCACTACGAACAGCGCCGCCAGGGCCG
Spelta          GATGGAGTGGGAGCGCCAGTGCGCCGCCCTGCAGCACTACGAACAGCGCCGCCAGGGCCG
Lancer          GATGGAGTGGGAGCGCCAGTGCGCCGCCCTGCAGCACTACGAACAGCGCCGCCAGGGCCG
Gladius         GATGGAGTGGGAGCGCCAGTGCGCCGCCCTGCAGCACTACGAACAGCGCCGCCAGGGCCG
Kukri           GATGGAGTGGGAGCGCCAGTGCGCCGCCCTGCAGCACTACGAACAGCGCCGCCAGGGCCG
Landmark        GATGGAGTGGGAGCGCCAGTGCGCCGCCCTGCAGCACTACGAACAGCGCCGCCAGGGCCG
Mace            GATGGAGTGGGAGCGCCAGTGCGCCGCCCTGCAGCACTACGAACAGCGCCGCCAGGGCCG
STANLEY         GATGGAGTGGGAGCGCCAGTGCGCCGCCCTGCAGCACTACGAACAGCGCCGCCAGGGCCG
                ************************************************************
                     v
Drysdale        CGACGAAGGAGGCGTCGTCGTCCTCAACGACAACGACGACGACGACGCGCCGCCACCGCC
RAC875          CGACGAAGGAGGCGTCGTCGTCCTCAACGACAACGACGACGACGACGCGCCGCCACCGCC
Norin61         CGACGAAGGAGGCGTCGTCGTCCTCAACGACAACGACGACGACGACGCGCCGCCACCGCC
Jagger          CGACGAAGGAGGCGTCGTCGTCCTCAACGACAACGACGACGACGACGCGCCGCCACCGCC
Mattis          CGACGAAGGAGGCGTCGTCGTCCTCAACGACAACGACGACGACGACGCGCCGCCACCGCC
Julius          CGACGAAGGAGGCGTCGTCGTCCTCAACGACAACGACGACGACGACGCGCCGCCACCGCC
Arina           CGACGAAGGAGGCGTCGTCGTCCTCAACGACAACGACGACGACGACGCGCCGCCACCGCC
Spelta          CGACGAAGGAGGCGTCGTCGTCCTCAACGACAACGACGACGACGACGCGCCGCCACCGCC
Lancer          CGACGAAGGAGGCGTCGTCGTGCTCAACGACAACGACGACGACGACGCGCCGCCACCGCC
Gladius         CGACGAAGGAGGCGTCGTCATCCTCAACGACAACGACGACGACGACGCGCCGCCACCGCC
Kukri           CGACGAAGGAGGCGTCGTCATCCTCAACGACAACGACGACGACGACGCGCCGCCACCGCC
Landmark        CGACGAAGGAGGCGTCGTCATCCTCAACGACAACGACGACGACGACGCGCCGCCACCGCC
Mace            CGACGAAGGAGGCGTCGTCATCCTCAACGACAACGACGACGACGACGCGCCGCCACCGCC
STANLEY         CGACGAAGGAGGCGTCGTCATCCTCAACGACAACGACGACGACGACGCGCCGCCACCGCC
                ******************* * **************************************

Drysdale        ACCGGTCCGCCATGGCGACGCCGGGCAAGGGTCCAGCAGGGGCGGCCGCGTCAAGGAGGA
RAC875          ACCGGTCCGCCATGGCGACGCCGGGCAAGGGTCCAGCAGGGGCGGCCGCGTCAAGGAGGA
Norin61         ACCGGTCCGCCATGGCGACGCCGGGCAAGGGTCCAGCAGGGGCGGCCGCGTCAAGGAGGA
Jagger          ACCGGTCCGCCATGGCGACGCCGGGCAAGGGTCCAGCAGGGGCGGCCGCGTCAAGGAGGA
Mattis          ACCGGTCCGCCATGGCGACGCCGGGCAAGGGTCCAGCAGGGGCGGCCGCGTCAAGGAGGA
Julius          ACCGGTCCGCCATGGCGACGCCGGGCAAGGGTCCAGCAGGGGCGGCCGCGTCAAGGAGGA
Arina           ACCGGTCCGCCATGGCGACGCCGGGCAAGGGTCCAGCAGGGGCGGCCGCGTCAAGGAGGA
Spelta          ACCGGTCCGCCATGGCGACGCCGGGCAAGGGTCCAGCAGGGGCGGCCGCGTCAAGGAGGA
Lancer          ACCGGTCCGCCATGGCGACGCCGGGCAAGGGTCCAGCAGGGGCGGCCGCGTCAAGGAGGA
Gladius         ACCGGTCCGCCATGGCGACGCCGGGCAAGGGTCCAGCGGGGGCGGCCGCGTCAAGGAGGA
Kukri           ACCGGTCCGCCATGGCGACGCCGGGCAAGGGTCCAGCGGGGGCGGCCGCGTCAAGGAGGA
Landmark        ACCGGTCCGCCATGGCGACGCCGGGCAAGGGTCCAGCAGGGGCGGCCGCGTCAAGGAGGA
Mace            ACCGGTCCGCCATGGCGACGCCGGGCAAGGGTCCAGCAGGGGCGGCCGCGTCAAGGAGGA
STANLEY         ACCGGTCCGCCATGGCGACGCCGGGCAAGGGTCCAGCAGGGGCGGCCGCGTCAAGGAGGA
                ************************************* **********************
```

```
Drysdale        GAAGACCGCCGCCGCCGACGACGCGCCGCCACCTCCACCGGTCCGCCATGGCGACGCCGG
RAC875          GAAGACCGCCGCCGCCGACGACGCGCCGCCACCTCCACCGGTCCGCCATGGCGACGCCGG
Norin61         GAAGACCGCCGCCGCCGACGACGCGCCGCCACCTCCACCGGTCCGCCATGGCGACGCCGG
Jagger          GAAGACCGCCGCCGCCGACGACGCGCCGCCACCTCCACCGGTCCGCCATGGCGACGCCGG
Mattis          GAAGACCGCCGCCGCCGACGACGCGCCGCCACCTCCACCGGTCCGCCATGGCGACGCCGG
Julius          GAAGACCGCCGCCGCCGACGACGCGCCGCCACCTCCACCGGTCCGCCATGGCGACGCCGG
Arina           GAAGACCGCCGCCGCCGACGACGCGCCGCCACCTCCACCGGTCCGCCATGGCGACGCCGG
Spelta          GAAGACCGCCGCCGCCGACGACGCGCCGCCACCTCCACCGGTCCGCCATGGCGACGCCGG
Lancer          GAAGACCGCCGCCGCCGACGACGCGCCGCCACCTCCGCCGGTCCGCCATGGCGACGCCGG
Gladius         GAAGACCGCCGCCGACGACGACGCGCCG----CTCCACCGGTCCGCCATGGCGACGCCGG
Kukri           GAAGACCGCCGCCGACGACGACGCGCCG----CTCCACCGGTCCGCCATGGCGACGCCGG
Landmark        GAAGACCGCCGCCGACGACGACGCGCCG----CTCCACCGGTCCGCCATGGCGACGCCGG
Mace            GAAGACCGCCGCCGACGACGACGCGCCG----CTCCACCGGTCCGCCATGGCGACGCCGG
STANLEY         GAAGACCGCCGCCGACGACGACGCGCCG----CTCCACCGGTCCGCCATGGCGACGCCGG
                ************** *************    **** ***********************

                v
Drysdale        ACAAGGGTCCAGCAGGGGCGGCCGCGTAAAGGAGGAGAAGGCCGCCGCCGACGACTACGC
RAC875          ACAAGGGTCCAGCAGGGGCGGCCGCGTAAAGGAGGAGAAGGCCGCCGCCGACGACTACGC
Norin61         ACAAGGGTCCAGCAGGGGCGGCCGCGTAAAGGAGGAGAAGGCCGCCGCCGACGACTACGC
Jagger          ACAAGGGTCCAGCAGGGGCGGCCGCGTAAAGGAGGAGAAGGCCGCCGCCGACGACTACGC
Mattis          ACAAGGGTCCAGCAGGGGCGGCCGCGTAAAGGAGGAGAAGGCCGCCGCCGACGACTACGC
Julius          ACAAGGGTCCAGCAGGGGCGGCCGCGTAAAGGAGGAGAAGGCCGCCGCCGACGACTACGC
Arina           ACAAGGGTCCAGCAGGGGCGGCCGCGTAAAGGAGGAGAAGGCCGCCGCCGACGACTACGC
Spelta          ACAAGGGTCCAGCAGGGGCGGCCGCGTAAAGGAGGAGAAGGCCGCCGCCGACGACTACGC
Lancer          GCAAGGGTCCAGCAGGGGCGGCCGCGTAAAGGAGGAGAAGGCCGCCGCCAACGACTACGC
Gladius         GCAAGGGTCCAGCAGGGGCGGCCGCGTAAAGGAGGAGAAGGCCGCCGCCGACGACTACGC
Kukri           GCAAGGGTCCAGCAGGGGCGGCCGCGTAAAGGAGGAGAAGGCCGCCGCCGACGACTACGC
Landmark        GCAAGGGTCCAGCAGGGGCGGCCGCGTAAAGGAGGAGAAGGCCGCCGCCGACGACTACGC
Mace            GCAAGGGTCCAGCAGGGGCGGCCGCGTAAAGGAGGAGAAGGCCGCCGCCGACGACTACGC
STANLEY         GCAAGGGTCCAGCAGGGGCGGCCGCGTAAAGGAGGAGAAGGCCGCCGCCGACGACTACGC
                 ************************************************ **********

                                                               v
Drysdale        CGCGTTCAGCGACTTCTTCGCACCTTCGTTGTAGTTTCGGCTTTTTT-AGATAAATTTGC
RAC875          CGCGTTCAGCGACTTCTTCGCACCTTCGTTGTAGTTTCGGCTTTTTT-AGATAAATTTGC
Norin61         CGCGTTCAGCGACTTCTTCGCACCTTCGTTGTAGTTTCGGCTTTTTT-AGATAAATTTGC
Jagger          CGCGTTCAGCGACTTCTTCGCACCTTCGTTGTAGTTTCGGCTTTTTT-AGATAAATTTGC
Mattis          CGCGTTCAGCGACTTCTTCGCACCTTCGTTGTAGTTTCGGCTTTTTT-AGATAAATTTGC
Julius          CGCGTTCAGCGACTTCTTCGCACCTTCGTTGTAGTTTCGGCTTTTTT-AGATAAATTTGC
Arina           CGCGTTCAGCGACTTCTTCGCACCTTCGTTGTAGTTTCGGCTTTTTT-AGATAAATTTGC
Spelta          CGCGTTCAGCGACTTCTTCGCACCTTCGTTGTAGTTTCGGCTTTTTT-AGATAAATTTGC
Lancer          CGCGTTCAGCGACTTCTTCGCACCTTCGTTGTAGTTTCGGCTTTTTTTAGATAAATTTGC
Gladius         CGCGTTCAGCGACTTCTTCGCACCTTCGTTGTAGTTTCGGCTTTTTTGAGATAAATTTGC
Kukri           CGCGTTCAGCGACTTCTTCGCACCTTCGTTGTAGTTTCGGCTTTTTTGAGATAAATTTGC
Landmark        CGCGTTCAGCGACTTCTTCGCACCTTCGTTGTAGTTTCGGCTTTTTTGAGATAAATTTGC
Mace            CGCGTTCAGCGACTTCTTCGCACCTTCGTTGTAGTTTCGGCTTTTTTGAGATAAATTTGC
STANLEY         CGCGTTCAGCGACTTCTTCGCACCTTCGTTGTAGTTTCGGCTTTTTTGAGATAAATTTGC
                *********************************************** ************

                   v
Drysdale        TATATAAACTGTCTTCTTTGTGTAATATATGTCCAGTTTACCGAATTTAGCCGAACTTTA
RAC875          TATATAAACTGTCTTCTTTGTGTAATATATGTCCAGTTTACCGAATTTAGCCGAACTTTA
Norin61         TATATAAACTGTCTTCTTTGTGTAATATATGTCCAGTTTACCGAATTTAGCCGAACTTTA
Jagger          TATATAAACTGTCTTCTTTGTGTAATATATGTCCAGTTTACCGAATTTAGCCGAACTTTA
Mattis          TATATAAACTGTCTTCTTTGTGTAATATATGTCCAGTTTACCGAATTTAGCCGAACTTTA
Julius          TATATAAACTGTCTTCTTTGTGTAATATATGTCCAGTTTACCGAATTTAGCCGAACTTTA
Arina           TATATAAACTGTCTTCTTTGTGTAATATATGTCCAGTTTACCGAATTTAGCCGAACTTTA
Spelta          TATATAAACTGTCTTCTTTGTGTAATATATGTCCAGTTTACCGAATTTAGCCGAACTTTA
Lancer          TATGTAAACTGTCTTCTTTGTGTAATATATGTCCAGTTTACCGAATTTAGCCGAACTTTA
Gladius         TATGTAAACTGTCTTCTTTGTGTAATATATGTCCAGTTTACCGAATTTAGCCGAACTTTA
Kukri           TATGTAAACTGTCTTCTTTGTGTAATATATGTCCAGTTTACCGAATTTAGCCGAACTTTA
Landmark        TATGTAAACTGTCTTCTTTGTGTAATATATGTCCAGTTTACCGAATTTAGCCGAACTTTA
Mace            TATGTAAACTGTCTTCTTTGTGTAATATATGTCCAGTTTACCGAATTTAGCCGAACTTTA
STANLEY         TATGTAAACTGTCTTCTTTGTGTAATATATGTCCAGTTTACCGAATTTAGCCGAACTTTA
                *** ********************************************************
```

```
                       v                             v                     v
Drysdale        TTGAATGTTTTTTTTTAATTTAGACGGCGTCTGGGAATGGTCATGGGATCGGCGACTGAG
RAC875          TTGAATGTTTTTTTTTAATTTAGACGGCGTCTGGGAATGGTCATGGGATCGGCGACTGAG
Norin61         TTGAATGTTTTTTTTTAATTTAGACGGCGTCTGGGAATGGTCATGGGATCGGCGACTGAG
Jagger          TTGAATGTTTTTTTTTAATTTAGACGGCGTCTGGGAATGGTCATGGGATCGGCGACTGAG
Mattis          TTGAATGTTTTTTTTTAATTTAGACGGCGTCTGGGAATGGTCATGGGATCGGCGACTGAG
Julius          TTGAATGTTTTTTTTTAATTTAGACGGCGTCTGGGAATGGTCATGGGATCGGCGACTGAG
Arina           TTGAATGTTTTTTTTTAATTTAGACGGCGTCTGGGAATGGTCATGGGATCGGCGACTGAG
Spelta          TTGAATGTTTTTTTTTAATTTAGACGGCGTCTGGGAATGGTCATGGGATCGGCGACTGAG
Lancer          TTGAATGTTTTTTTTTAATTTAGACGGTGTCTAGGAACGGTCATGGGATCGGCGACTGAG
Gladius         TTGAATG-TTTTTTTTAATTTAGACGGCGTCTGGGAACGGTCATGGGATCGGCGACTGGG
Kukri           TTGAATG-TTTTTTTTAATTTAGACGGCGTCTGGGAACGGTCATGGGATCGGCGACTGGG
Landmark        TTGAATG-TTTTTTTTAATTTAGACGGCGTCTGGGAACGGTCATGGGATCGGCGACTGGG
Mace            TTGAATG-TTTTTTTTAATTTAGACGGCGTCTGGGAACGGTCATGGGATCGGCGACTGGG
STANLEY         TTGAATG-TTTTTTTTAATTTAGACGGCGTCTGGGAACGGTCATGGGATCGGCGACTGGG
                ******* ******************* **** **** ******************** *

                          v  vv                                      v
Drysdale        AATCCAGTTGCCCCCAGGCGGCGATTTTACGCTCCCCTTGGGGAACGGCTGGAGATGCTC
RAC875          AATCCAGTTGCCCCCAGGCGGCGATTTTACGCGCCCCTTGGGGAACGGCTGGAGATGCTC
Norin61         AATCCAGTTGCCCCCAGGCGGCGATTTTACGCGCCCCTTGGGGAACGGCTGGAGATGCTC
Jagger          AATCCAGTTGCCCCCAGGCGGCGATTTTACGCGCCCCTTGGGGAACGGCTGGAGATGCTC
Mattis          AATCCAGTTGCCCCCAGGCGGCGATTTTACGCGCCCCTTGGGGAACGGCTGGAGATGCTC
Julius          AATCCAGTTGCCCCCAGGCGGCGATTTTACGCGCCCCTTGGGGAACGGCTGGAGATGCTC
Arina           AATCCAGTTGCCCCCAGGCGGCGATTTTACGCGCCCCTTGGGGAACGGCTGGAGATGCTC
Spelta          AATCCAGTTGCCCCCAGGCGGCGATTTTACGCGCCCCTTGGGGAACGGCTGGAGATGCTC
Lancer          AATCCAGTTGCCCCCAGACGGCGATTTTACCCGCCCCTT-GGGAACGGCTGTAGATGCTC
Gladius         AATCCAGTTG-CCTTAGGCGGCGATTTTACGCGCCCCTTGGGGAACGGCTGGAGATGGTC
Kukri           AATCCAGTTG-CCTTAGGCGGCGATTTTACGCGCCCCTTGGGGAACGGCTGGAGATGGTC
Landmark        AATCCAGTTG-CCTTAGGCGGCGATTTTACGCGCCCCTTGGGGAACGGCTGGAGATGGTC
Mace            AATCCAGTTG-CCTTAGGCGGCGATTTTACGCGCCCCTTGGGGAACGGCTGGAGATGGTC
STANLEY         AATCCAGTTG-CCTTAGGCGGCGATTTTACGCGCCCCTTGGGGAACGGCTGGAGATGGTC
                ********** **  ** ************ * ****** *********** ***** **

Drysdale        TGAGTCTCCTTGATGCCCGAGCTCTCGGATGAATGGCAGATTTCTTTTGAATGTTGCAGT
RAC875          TGAGTCTCCTTGATGCCCGAGCTCTCGGATGAATGGCAGATTTCTTTTGAATGTTGCAGT
Norin61         TGAGTCTCCTTGATGCCCGAGCTCTCGGATGAATGGCAGATTTCTTTTGAATGTTGCAGT
Jagger          TGAGTCTCCTTGATGCCCGAGCTCTCGGATGAATGGCAGATTTCTTTTGAATGTTGCAGT
Mattis          TGAGTCTCCTTGATGCCCGAGCTCTCGGATGAATGGCAGATTTCTTTTGAATGTTGCAGT
Julius          TGAGTCTCCTTGATGCCCGAGCTCTCGGATGAATGGCAGATTTCTTTTGAATGTTGCAGT
Arina           TGAGTCTCCTTGATGCCCGAGCTCTCGGATGAATGGCAGATTTCTTTTGAATGTTGCAGT
Spelta          TGAGTCTCCTTGATGCCCGAGCTCTCGGATGAATGGCAGATTTCTTTTGAATGTTGCAGT
Lancer          TGAGTCTCCTTGATGCCCGAGCTCTCGGATGAATGGCAGATTTCTTTTGAATGTTGCAGT
Gladius         TGAGTCTCCTTGATGCCCGAGCTCTCGGATGAATGGCNNNNNNNNNNNNNNNNNNNNNNN
Kukri           TGAGTCTCCTTGATGCCCGAGCTCTCGGATGAATGGCAGATTTCTTTTGAATGTTGCAGT
Landmark        TGAGTCTCCTTGATGCCCGAGCTCTCGGATGAATGGCAGATTTCTTTTGAATGTTGCAGT
Mace            TGAGTCTCCTTGATGCCCGAGCTCTCGGATGAATGGCAGATTTCTTTTGAATGTTGCAGT
STANLEY         TGAGTCTCCTTGATGCCCGAGCTCTCGGATGAATGGCAGATTTCTTTTGAATGTTGCAGT
                *************************************

Drysdale        TGGGTGAATGCGGTTAGGTTAGGTGGATGGTAAAAAGTAAAAACAACTTCTATTCCCAAA
RAC875          TGGGTGAATGCGGTTAGGTTAGGTGGATGGTAAAAAGTAAAAACAACTTCTATTCCCAAA
Norin61         TGGGTGAATGCGGTTAGGTTAGGTGGATGGTAAAAAGTAAAAACAACTTCTATTCCCAAA
Jagger          TGGGTGAATGCGGTTAGGTTAGGTGGATGGTAAAAAGTAAAAACAACTTCTATTCCCAAA
Mattis          TGGGTGAATGCGGTTAGGTTAGGTGGATGGTAAAAAGTAAAAACAACTTCTATTCCCAAA
Julius          TGGGTGAATGCGGTTAGGTTAGGTGGATGGTAAAAAGTAAAAACAACTTCTATTCCCAAA
Arina           TGGGTGAATGCGGTTAGGTTAGGTGGATGGTAAAAAGTAAAAACAACTTCTATTCCCAAA
Spelta          TGGGTGAATGCGGTTAGGTTAGGTGGATGGTAAAAAGTAAAAACAACTTCTATTCCCAAA
Lancer          TGGGTGAATGCGGTTAGGTTAGGTGGATGGTAAAAAGTAAAAACAACTTCTATTCCCAAA
Gladius         NNNNNNNNNNNNNNNNNNNNNNNNNNNNNNNNNNNNNNNNNNNNNNNNNNNNNNNNNNNN
Kukri           TGGGTGAATGCGGTTAGGTTAGGTGGATGGTAAAAAGTAAAAACAACTTCTATTCCCAAA
Landmark        TGGGTGAATGCGGTTAGGTTAGGTGGATGGTAAAAAGTAAAAACAACTTCTATTCCCAAA
Mace            TGGGTGAATGCGGTTAGGTTAGGTGGATGGTAAAAAGTAAAAACAACTTCTATTCCCAAA
STANLEY         TGGGTGAATGCGGTTAGGTTAGGTGGATGGTAAAAAGTAAAAACAACTTCTATTCCCAAA
```

```
Drysdale        GTCTGAACCCGTCAAAATCTGGCTATGGGCACGGCCCCATCTCATCAGAGACCCCACCAG
RAC875          GTCTGAACCCGTCAAAATCTGGCTATGGGCACGGCCCCATCTCATCAGAGACCCCACCAG
Norin61         GTCTGAACCCGTCAAAATCTGGCTATGGGCACGGCCCCATCTCATCAGAGACCCCACCAG
Jagger          GTCTGAACCCGTCAAAATCTGGCTATGGGCACGGCCCCATCTCATCAGAGACCCCACCAG
Mattis          GTCTGAACCCGTCAAAATCTGGCTATGGGCACGGCCCCATCTCATCAGAGACCCCACCAG
Julius          GTCTGAACCCGTCAAAATCTGGCTATGGGCACGGCCCCATCTCATCAGAGACCCCACCAG
Arina           GTCTGAACCCGTCAAAATCTGGCTATGGGCACGGCCCCATCTCATCAGAGACCCCACCAG
Spelta          GTCTGAACCCGTCAAAATCTGGCTATGGGCACGGCCCCATCTCATCAGAGACCCCACCAG
Lancer          GTCTGAACCCGTCAAAATCTGGCTATGGGCACGGCCCCATCTCATCAGAGACCCCACCAG
Gladius         NNNNNNNNNNNNNNNNNNNNNNNNNNNNNNNNNNNNNNNNNNNNNNNNNNNNNNNNNNNN
Kukri           GTCTGAACCCGTCAAAATCTGGCTATGGGCACGGCCCCATCTCATCAGAGACCCCACCAG
Landmark        GTCTGAACCCGTCAAAATCTGGCTATGGGCACGGCCCCATCTCATCAGAGACCCCACCAG
Mace            GTCTGAACCCGTCAAAATCTGGCTATGGGCACGGCCCCATCTCATCAGAGACCCCACCAG
STANLEY         GTCTGAACCCGTCAAAATCTGGCTATGGGCACGGCCCCATCTCATCAGAGACCCCACCAG

Drysdale        ACGTCCTGTGACGGGGCTCGCACCCGATACCATTTTGATCGGATCCATACCCCTTAACCC
RAC875          ACCTCCTGTGACGGGGCTCGCACCCGATACCATCTTGATCGGATCCATACCCCTTAACCC
Norin61         ACCTCCTGTGACGGGGCTCGCACCCGATACCATCTTGATCGGATCCATACCCCTTAACCC
Jagger          ACCTCCTGTGACGGGGCTCGCACCCGATACCATCTTGATCGGATCCATACCCCTTAACCC
Mattis          ACCTCCTGTGACGGGGCTCGCACCCGATACCATCTTGATCGGATCCATACCCCTTAACCC
Julius          ACCTCCTGTGACGGGGCTCGCACCCGATACCATCTTGATCGGATCCATACCCCTTAACCC
Arina           ACCTCCTGTGACGGGGCTCGCACCCGATACCATCTTGATCGGATCCATACCCCTTAACCC
Spelta          ACCTCCTGTGACGGGGCTCGCACCCGATACCATCTTGATCGGATCCATACCCCTTAACCC
Lancer          ACCTCCTGTGACGGGGCTCGCACCCGATACCATCTTGATCGGATCCATACCCCTTAACCC
Gladius         NNNNNNNNNNNNNNNNNNNNNNNNNNNNNNNNNNNNNNNNNNNNNNNNNNNNNNNNNNNN
Kukri           ACCTCCTGTGACGGGGCTCGCACCCGATACCATCTTGATCGGATCCATACCCCTTAACCC
Landmark        ACCTCCTGTGACGGGGCTCGCACCCGATACCATCTTGATCGGATCCATACCCCTTAACCC
Mace            ACCTCCTGTGACGGGGCTCGCACCCGATACCATCTTGATCGGATCCATACCCCTTAACCC
STANLEY         ACCTCCTGTGACGGGGCTCGCACCCGATACCATCTTGATCGGATCCATACCCCTTAACCC

Drysdale        CCTTGACCTCACCTTCGCTCACAAGCGCCAGTACACAGAGCAGAGAGCCGCCCGCCTGAG
RAC875          CCTTGACCTCACCTTCGCTCACAAGCGCCAGTACACAGAGCAGAGAGCCGCCCGCCTGAG
Norin61         CCTTGACCTCACCTTCGCTCACAAGCGCCAGTACACAGAGCAGAGAGCCGCCCGCCTGAG
Jagger          CCTTGACCTCACCTTCGCTCACAAGCGCCAGTACACAGAGCAGAGAGCCGCCCGCCTGAG
Mattis          CCTTGACCTCACCTTCGCTCACAAGCGCCAGTACACAGAGCAGAGAGCCGCCCGCCTGAG
Julius          CCTTGACCTCACCTTCGCTCACAAGCGCCAGTACACAGAGCAGAGAGCCGCCCGCCTGAG
Arina           CCTTGACCTCACCTTCGCTCACAAGCGCCAGTACACAGAGCAGAGAGCCGCCCGCCTGAG
Spelta          CCTTGACCTCACCTTCGCTCACAAGCGCCAGTACACAGAGCAGAGAGCCGCCCGCCTGAG
Lancer          CCTTGACCTCACCTTCGCTCACAAGCGCCAGTACACAGAGCAGAGAGCCGCCCGCCTGAG
Gladius         NNNNNNNNNNNNNNNNNNNNNNNNNNNNNNNNNNNNNNNNNNNNNNNNNNNNNNNNNNNN
Kukri           CCTTGACCTCACCTTCGCTCACAAGCGCCAGTACACAGAGCAGAGAGCCGCCCGCCTGAG
Landmark        CCTTGACCTCACCTTCGCTCACAAGCGCCAGTACACAGAGCAGAGAGCCGCCCGCCTGAG
Mace            CCTTGACCTCACCTTCGCTCACAAGCGCCAGTACACAGAGCAGAGAGCCGCCCGCCTGAG
STANLEY         CCTTGACCTCACCTTCGCTCACAAGCGCCAGTACACAGAGCAGAGAGCCGCCCGCCTGAG

Drysdale        TGCTACCCGCCGCTGCTGGTGCCAACAGACAG----CCGCCCGCCCGCCCGCCCGCCTGA
RAC875          TGCTACCCGCCGCTGCTGGTGCCAACAGACAG----CCGCCCGCCCGCCCGCCCGCCTGA
Norin61         TGCTACCCGCCGCTGCTGGTGCCAACAGACAG----CCGCCCGCCCGCCCGCCCGCCTGA
Jagger          TGCTACCCGCCGCTGCTGGTGCCAACAGACAG----CCGCCCGCCCGCCCGCCCGCCTGA
Mattis          TGCTACCCGCCGCTGCTGGTGCCAACAGACAG----CCGCCCGCCCGCCCGCCCGCCTGA
Julius          TGCTACCCGCCGCTGCTGGTGCCAACAGACAG----CCGCCCGCCCGCCCGCCCGCCTGA
Arina           TGCTACCCGCCGCTGCTGGTGCCAACAGACAG----CCGCCCGCCCGCCCGCCCGCCTGA
Spelta          TGCTACCCGCCGCTGCTGGTGCCAACAGACAG----CCGCCCGCCCGCCCGCCCGCCTGA
Lancer          TGCTACCCGCCGCTGCTGGTGCCAACAGAGAGCCGCCCGCCCGCCCGCCCGCCCGCCTGA
Gladius         NNNNNNNNNNNNNNNNNNNNNNNNNNNNNNNNNNNNNNNNNNNNNNNNNNNNNNNNNNNN
Kukri           TGCTACCCGCCGCTGCTGGTGCCAACAGAGAG----CCGCCCGCCCGCCCGCCCGCCTGA
Landmark        TGCTACCCGCCGCTGCTGGTGCCAACAGAGAG----CCGCCCGCCCGCCCGCCCGCCTGA
Mace            TGCTACCCGCCGCTGCTGGTGCCAACAGAGAG----CCGCCCGCCCGCCCGCCCGCCTGA
STANLEY         TGCTACCCGCCGCTGCTGGTGCCAACAGAGAG----CCGCCCGCCCGCCCGCCCGCCTGA
```

```
                                              +1
Drysdale        CTGAATCTTGGTGGGGGAGGAGAGGAGAGATGGGCCTCGGCGGCGGCGGAGAGGGGA
RAC875          CTGAATCTTGGTGGGGGAGGAGAGGAGAGATGGGCCTCGGCGGCGGCGGAGAGGGGA
Norin61         CTGAATCTTGGTGGGGGAGGAGAGGAGAGATGGGCCTCGGCGGCGGCGGAGAGGGGA
Jagger          CTGAATCTTGGTGGGGGAGGAGAGGAGAGATGGGCCTCGGCGGCGGCGGAGAGGGGA
Mattis          CTGAATCTTGGTGGGGGAGGAGAGGAGAGATGGGCCTCGGCGGCGGCGGAGAGGGGA
Julius          CTGAATCTTGGTGGGGGAGGAGAGGAGAGATGGGCCTCGGCGGCGGCGGAGAGGGGA
Arina           CTGAATCTTGGTGGGGGAGGAGAGGAGAGATGGGCCTCGGCGGCGGCGGAGAGGGGA
Spelta          CTGAATCTTGGTGGGGGAGGAGAGGAGAGATGGGCCTCGGCGGCGGCGGAGAGGGGA
Lancer          CTGAATCTTGGTGGGGGAGGAGAGGAGAGATGGGCCTCGGCGGCGGCGGAGAGGGGA
Gladius         NNNNNNNNNNNNNNNNNNNNNNNNNNNNNNNNNNNNNNNNNNNNNNNNNNNNNNNNNNNN
Kukri           CTGAATCTTGGTGGGGGAGGAGAGGAGAGATGGGCCTCGGCGGCGGCGGAGAGGGGA
Landmark        CTGAATCTTGGTGGGGGAGGAGAGGAGAGATGGGCCTCGGCGGCGGCGGAGAGGGGA
Mace            CTGAATCTTGGTGGGGGAGGAGAGGAGAGATGGGCCTCGGCGGCGGCGGAGAGGGGA
STANLEY         CTGAATCTTGGTGGGGGAGGAGAGGAGAGATGGGCCTCGGCGGCGGCGGAGAGGGGA


Drysdale        TCCTGGCCGTGGCCGTGGTGCCCATGGAGGCCATCCCCATGGCGGAGCCGCAGATCCTCG
RAC875          TCCTGGCCGTGGCCGTGGTGCCCATGGAGGCCATCCCCATGGCGGAGCCGCAGATCCTCG
Norin61         TCCTGGCCGTGGCCGTGGTGCCCATGGAGGCCATCCCCATGGCGGAGCCGCAGATCCTCG
Jagger          TCCTGGCCGTGGCCGTGGTGCCCATGGAGGCCATCCCCATGGCGGAGCCGCAGATCCTCG
Mattis          TCCTGGCCGTGGCCGTGGTGCCCATGGAGGCCATCCCCATGGCGGAGCCGCAGATCCTCG
Julius          TCCTGGCCGTGGCCGTGGTGCCCATGGAGGCCATCCCCATGGCGGAGCCGCAGATCCTCG
Arina           TCCTGGCCGTGGCCGTGGTGCCCATGGAGGCCATCCCCATGGCGGAGCCGCAGATCCTCG
Spelta          TCCTGGCCGTGGCCGTGGTGCCCATGGAGGCCATCCCCATGGCGGAGCCGCAGATCCTCG
Lancer          TCCTGGCCGTGGCCGTGGTGCCCATGGAGGCCATCCCCATGGCGGAGCCGCAGATCCTCG
Gladius         NNNNNNNNNNNNNNNNNNNNNNNNNNNNNNNNNNNNNNNNNNNNNNNNNNNNNNNNNNNN
Kukri           TCCTGGCCGTGGCCGTGGTGCCCATGGAGGCCATCCCCATGGCGGAGCCGCAGATCCTCG
Landmark        TCCTGGCCGTGGCCGTGGTGCCCATGGAGGCCATCCCCATGGCGGAGCCGCAGATCCTCG
Mace            TCCTGGCCGTGGCCGTGGTGCCCATGGAGGCCATCCCCATGGCGGAGCCGCAGATCCTCG
STANLEY         TCCTGGCCGTGGCCGTGGTGCCCATGGAGGCCATCCCCATGGCGGAGCCGCAGATCCTCG


Drysdale        CCGGCGGCAAGACCGTCGGCCTGCGGGCGGACCTGCTCGACTGCCACAACTGCCGCCTCC
RAC875          CCGGCGGCAAGACCGTCGGCCTGCGGGCGGACCTGCTCGACTGCCACAACTGCCGCCTCC
Norin61         CCGGCGGCAAGACCGTCGGCCTGCGGGCGGACCTGCTCGACTGCCACAACTGCCGCCTCC
Jagger          CCGGCGGCAAGACCGTCGGCCTGCGGGCGGACCTGCTCGACTGCCACAACTGCCGCCTCC
Mattis          CCGGCGGCAAGACCGTCGGCCTGCGGGCGGACCTGCTCGACTGCCACAACTGCCGCCTCC
Julius          CCGGCGGCAAGACCGTCGGCCTGCGGGCGGACCTGCTCGACTGCCACAACTGCCGCCTCC
Arina           CCGGCGGCAAGACCGTCGGCCTGCGGGCGGACCTGCTCGACTGCCACAACTGCCGCCTCC
Spelta          CCGGCGGCAAGACCGTCGGCCTGCGGGCGGACCTGCTCGACTGCCACAACTGCCGCCTCC
Lancer          CCGGCGGCAAGACCGTCGGCCTGCGGGCGGACCTGCTCGACTGCCACAACTGCCGCCTCC
Gladius         NNNNNNNNNAGACCGTCGGCCTGCGGGCGGACCTGCTCGACTGCCACAACTGCCGCCTCC
Kukri           CCGGCGGCAAGACCGTCGGCCTGCGGGCGGACCTGCTCGACTGCCACAACTGCCGCCTCC
Landmark        CCGGCGGCAAGACCGTCGGCCTGCGGGCGGACCTGCTCGACTGCCACAACTGCCGCCTCC
Mace            CCGGCGGCAAGACCGTCGGCCTGCGGGCGGACCTGCTCGACTGCCACAACTGCCGCCTCC
STANLEY         CCGGCGGCAAGACCGTCGGCCTGCGGGCGGACCTGCTCGACTGCCACAACTGCCGCCTCC
                         ***************************************************
                                                                 v
Drysdale        CCCTCAAGCCCCCCATATTCAAGGTGAGATCCGCCGGCCGCCCCTCTTCTCCAAGATTCA
RAC875          CCCTCAAGCCCCCCATATTCAAGGTGAGATCCGCCGGCCGCCCCTCTTCTCCAAGATTCA
Norin61         CCCTCAAGCCCCCCATATTCAAGGTGAGATCCGCCGGCCGCCCCTCTTCTCCAAGATTCA
Jagger          CCCTCAAGCCCCCCATATTCAAGGTGAGATCCGCCGGCCGCCCCTCTTCTCCAAGATTCA
Mattis          CCCTCAAGCCCCCCATATTCAAGGTGAGATCCGCCGGCCGCCCCTCTTCTCCAAGATTCA
Julius          CCCTCAAGCCCCCCATATTCAAGGTGAGATCCGCCGGCCGCCCCTCTTCTCCAAGATTCA
Arina           CCCTCAAGCCCCCCATATTCAAGGTGAGATCCGCCGGCCGCCCCTCTTCTCCAAGATTCA
Spelta          CCCTCAAGCCCCCCATATTCAAGGTGAGATCCGCCGGCCGCCCCTCTTCTCCAAGATTCA
Lancer          CCCTCAAGCCCCCCATATTCAAGGTGAGATCCGCCGGCCGCCCCTCTTCTCCAAGATTCA
Gladius         CCCTCAAGCCCCCCATATTCAAGGTGAGATCCGCCGGCCGCCCCTCTTCCCCAAGATTCA
Kukri           CCCTCAAGCCCCCCATATTCAAGGTGAGATCCGCCGGCCGCCCCTCTTCCCCAAGATTCA
Landmark        CCCTCAAGCCCCCCATATTCAAGGTGAGATCCGCCGGCCGCCCCTCTTCCCCAAGATTCA
Mace            CCCTCAAGCCCCCCATATTCAAGGTGAGATCCGCCGGCCGCCCCTCTTCCCCAAGATTCA
STANLEY         CCCTCAAGCCCCCCATATTCAAGGTGAGATCCGCCGGCCGCCCCTCTTCCCCAAGATTCA
                ************************************************* **********
```

```
Drysdale        TTTTTCCGTTTTCTTGAATCTGGCACGGCGTGACCGAGCGTATCTGGTGTGCATGAATCT
RAC875          TTTTTCCGTTTTCTTGAATCTGGCACGGCGTGACCGAGCGTATCTGGTGTGCATGAATCT
Norin61         TTTTTCCGTTTTCTTGAATCTGGCACGGCGTGACCGAGCGTATCTGGTGTGCATGAATCT
Jagger          TTTTTCCGTTTTCTTGAATCTGGCACGGCGTGACCGAGCGTATCTGGTGTGCATGAATCT
Mattis          TTTTTCCGTTTTCTTGAATCTGGCACGGCGTGACCGAGCGTATCTGGTGTGCATGAATCT
Julius          TTTTTCCGTTTTCTTGAATCTGGCACGGCGTGACCGAGCGTATCTGGTGTGCATGAATCT
Arina           TTTTTCCGTTTTCTTGAATCTGGCACGGCGTGACCGAGCGTATCTGGTGTGCATGAATCT
Spelta          TTTTTCCGTTTTCTTGAATCTGGCACGGCGTGACCGAGCGTATCTGGTGTGCATGAATCT
Lancer          TTTTTCCGTTTTCTTGAATCTGGCACGGCGTGACCGAGCGTATCTGGTGTGCATGAATCT
Gladius         TTTTTCCGTTTTCTTGAATCTGGCACGGCGTGACCGAGCGTATCTGGTGTGCATGAATCT
Kukri           TTTTTCCGTTTTCTTGAATCTGGCACGGCGTGACCGAGCGTATCTGGTGTGCATGAATCT
Landmark        TTTTTCCGTTTTCTTGAATCTGGCACGGCGTGACCGAGCGTATCTGGTGTGCATGAATCT
Mace            TTTTTCCGTTTTCTTGAATCTGGCACGGCGTGACCGAGCGTATCTGGTGTGCATGAATCT
STANLEY         TTTTTCCGTTTTCTTGAATCTGGCACGGCGTGACCGAGCGTATCTGGTGTGCATGAATCT
                ************************************************************

Drysdale        TGGCCGGCAGTGCGACGCCGAGCACCTGGTCTGCTCCTCCTGCCGCGGCGCCCACGCCGA
RAC875          TGGCCGGCAGTGCGACGCCGAGCACCTGGTCTGCTCCTCCTGCCGCGGCGCCCACGCCGA
Norin61         TGGCCGGCAGTGCGACGCCGAGCACCTGGTCTGCTCCTCCTGCCGCGGCGCCCACGCCGA
Jagger          TGGCCGGCAGTGCGACGCCGAGCACCTGGTCTGCTCCTCCTGCCGCGGCGCCCACGCCGA
Mattis          TGGCCGGCAGTGCGACGCCGAGCACCTGGTCTGCTCCTCCTGCCGCGGCGCCCACGCCGA
Julius          TGGCCGGCAGTGCGACGCCGAGCACCTGGTCTGCTCCTCCTGCCGCGGCGCCCACGCCGA
Arina           TGGCCGGCAGTGCGACGCCGAGCACCTGGTCTGCTCCTCCTGCCGCGGCGCCCACGCCGA
Spelta          TGGCCGGCAGTGCGACGCCGAGCACCTGGTCTGCTCCTCCTGCCGCGGCGCCCACGCCGA
Lancer          TGGCCGGCAGTGCGACGCCGAGCACCTGGTCTGCTCCTCCTGCCGCGGCGCCCACGCCGA
Gladius         TGGCCGGCAGTGCGACGCCGAGCACCTGGTCTGCTCCTCCTGCCGCGGCGCCCACGCCGA
Kukri           TGGCCGGCAGTGCGACGCCGAGCACCTGGTCTGCTCCTCCTGCCGCGGCGCCCACGCCGA
Landmark        TGGCCGGCAGTGCGACGCCGAGCACCTGGTCTGCTCCTCCTGCCGCGGCGCCCACGCCGA
Mace            TGGCCGGCAGTGCGACGCCGAGCACCTGGTCTGCTCCTCCTGCCGCGGCGCCCACGCCGA
STANLEY         TGGCCGGCAGTGCGACGCCGAGCACCTGGTCTGCTCCTCCTGCCGCGGCGCCCACGCCGA
                ************************************************************

Drysdale        GGCCTGCGGCGGCCGCGCCGCCGTCCACTCCGCGCTCGCCGACATCTTCGCGGCCGCCGC
RAC875          GGCCTGCGGCGGCCGCGCCGCCGTCCACTCCGCGCTCGCCGACATCTTCGCGGCCGCCGC
Norin61         GGCCTGCGGCGGCCGCGCCGCCGTCCACTCCGCGCTCGCCGACATCTTCGCGGCCGCCGC
Jagger          GGCCTGCGGCGGCCGCGCCGCCGTCCACTCCGCGCTCGCCGACATCTTCGCGGCCGCCGC
Mattis          GGCCTGCGGCGGCCGCGCCGCCGTCCACTCCGCGCTCGCCGACATCTTCGCGGCCGCCGC
Julius          GGCCTGCGGCGGCCGCGCCGCCGTCCACTCCGCGCTCGCCGACATCTTCGCGGCCGCCGC
Arina           GGCCTGCGGCGGCCGCGCCGCCGTCCACTCCGCGCTCGCCGACATCTTCGCGGCCGCCGC
Spelta          GGCCTGCGGCGGCCGCGCCGCCGTCCACTCCGCGCTCGCCGACATCTTCGCGGCCGCCGC
Lancer          GGCCTGCGGCGGCCGCGCCGCCGTCCACTCCGCGCTCGCCGACATCTTCGCGGCCGCCGC
Gladius         GGCCTGCGGCGGCCGCGCCGCCGTCCACTCCGCGCTCGCCGACATCTTCGCGGCCGCCGC
Kukri           GGCCTGCGGCGGCCGCGCCGCCGTCCACTCCGCGCTCGCCGACATCTTCGCGGCCGCCGC
Landmark        GGCCTGCGGCGGCCGCGCCGCCGTCCACTCCGCGCTCGCCGACATCTTCGCGGCCGCCGC
Mace            GGCCTGCGGCGGCCGCGCCGCCGTCCACTCCGCGCTCGCCGACATCTTCGCGGCCGCCGC
STANLEY         GGCCTGCGGCGGCCGCGCCGCCGTCCACTCCGCGCTCGCCGACATCTTCGCGGCCGCCGC
                ************************************************************

                                                       v
Drysdale        CACCGTGCCCTGCGGCTACGAGCGCTACGGCTGCGACGCCGGCGGCGTGGTGTACCACGA
RAC875          CACCGTGCCCTGCGGCTACGAGCGCTACGGCTGCGACGCCGGCGGCGTGGTGTACCACGA
Norin61         CACCGTGCCCTGCGGCTACGAGCGCTACGGCTGCGACGCCGGCGGCGTGGTGTACCACGA
Jagger          CACCGTGCCCTGCGGCTACGAGCGCTACGGCTGCGACGCCGGCGGCGTGGTGTACCACGA
Mattis          CACCGTGCCCTGCGGCTACGAGCGCTACGGCTGCGACGCCGGCGGCGTGGTGTACCACGA
Julius          CACCGTGCCCTGCGGCTACGAGCGCTACGGCTGCGACGCCGGCGGCGTGGTGTACCACGA
Arina           CACCGTGCCCTGCGGCTACGAGCGCTACGGCTGCGACGCCGGCGGCGTGGTGTACCACGA
Spelta          CACCGTGCCCTGCGGCTACGAGCGCTACGGCTGCGACGCCGGCGGCGTGGTGTACCACGA
Lancer          CACCGTGCCCTGCGGCTACGAGCGCTACGGCTGCGACGCGGGCGGCGTGGTGTACCACGA
Gladius         CACCGTGCCCTGCGGCTACGAGCGCTACGGCTGCGACGCGGGCGGCGTGGTGTACCACGA
Kukri           CACCGTGCCCTGCGGCTACGAGCGCTACGGCTGCGACGCGGGCGGCGTGGTGTACCACGA
Landmark        CACCGTGCCCTGCGGCTACGAGCGCTACGGCTGCGACGCGGGCGGCGTGGTGTACCACGA
Mace            CACCGTGCCCTGCGGCTACGAGCGCTACGGCTGCGACGCGGGCGGCGTGGTGTACCACGA
STANLEY         CACCGTGCCCTGCGGCTACGAGCGCTACGGCTGCGACGCGGGCGGCGTGGTGTACCACGA
                *************************************** ********************
```

```
                     v
Drysdale          GGCCGCCGACCACCGGCGCGCGTGCCAGCACGCGCCCTGCTGCTGCCCGGACCGCGCGGG
RAC875            GGCCGCCGACCACCGGCGCGCGTGCCAGCACGCGCCCTGCTGCTGCCCGGACCGCGCGGG
Norin61           GGCCGCCGACCACCGGCGCGCGTGCCAGCACGCGCCCTGCTGCTGCCCGGACCGCGCGGG
Jagger            GGCCGCCGACCACCGGCGCGCGTGCCAGCACGCGCCCTGCTGCTGCCCGGACCGCGCGGG
Mattis            GGCCGCCGACCACCGGCGCGCGTGCCAGCACGCGCCCTGCTGCTGCCCGGACCGCGCGGG
Julius            GGCCGCCGACCACCGGCGCGCGTGCCAGCACGCGCCCTGCTGCTGCCCGGACCGCGCGGG
Arina             GGCCGCCGACCACCGGCGCGCGTGCCAGCACGCGCCCTGCTGCTGCCCGGACCGCGCGGG
Spelta            GGCCGCCGACCACCGGCGCGCGTGCCAGCACGCGCCCTGCTGCTGCCCGGACCGCGCGGG
Lancer            GGCGGCCGACCACCGGCGCGCGTGCCAGCACGCGCCCTGCTGCTGCCCGGACCGCGCGGG
Gladius           GGCGGCCGACCACCGGCGCGCGTGCCAGCACGCGCCCTGCTGCTGCCCGGACCGCGCGGG
Kukri             GGCGGCCGACCACCGGCGCGCGTGCCAGCACGCGCCCTGCTGCTGCCCGGACCGCGCGGG
Landmark          GGCGGCCGACCACCGGCGCGCGTGCCAGCACGCGCCCTGCTGCTGCCCGGACCGCGCGGG
Mace              GGCGGCCGACCACCGGCGCGCGTGCCAGCACGCGCCCTGCTGCTGCCCGGACCGCGCGGG
STANLEY           GGCGGCCGACCACCGGCGCGCGTGCCAGCACGCGCCCTGCTGCTGCCCGGACCGCGCGGG
                  *** ********************************************************

Drysdale          CGCGGCCGGCATCGGGGGCTGCGGCTTCGTCGGCTCCCGCCAGGACCTGCTCGACCACAT
RAC875            CGCGGCCGGCATCGGGGGCTGCGGCTTCGTCGGCTCCCGCCAGGACCTGCTCGACCACAT
Norin61           CGCGGCCGGCATCGGGGGCTGCGGCTTCGTCGGCTCCCGCCAGGACCTGCTCGACCACAT
Jagger            CGCGGCCGGCATCGGGGGCTGCGGCTTCGTCGGCTCCCGCCAGGACCTGCTCGACCACAT
Mattis            CGCGGCCGGCATCGGGGGCTGCGGCTTCGTCGGCTCCCGCCAGGACCTGCTCGACCACAT
Julius            CGCGGCCGGCATCGGGGGCTGCGGCTTCGTCGGCTCCCGCCAGGACCTGCTCGACCACAT
Arina             CGCGGCCGGCATCGGGGGCTGCGGCTTCGTCGGCTCCCGCCAGGACCTGCTCGACCACAT
Spelta            CGCGGCCGGCATCGGGGGCTGCGGCTTCGTCGGCTCCCGCCAGGACCTGCTCGACCACAT
Lancer            CGCGGCCGGCATCGGGGGCTGCGGCTTCGTCGGCTCCCGCCAGGACCTGCTCGACCACAT
Gladius           CGCGGCCGGCATCGGGGGCTGCGGCTTCGTCGGCTCCCGCCAGGACCTGCTCGACCACAT
Kukri             CGCGGCCGGCATCGGGGGCTGCGGCTTCGTCGGCTCCCGCCAGGACCTGCTCGACCACAT
Landmark          CGCGGCCGGCATCGGGGGCTGCGGCTTCGTCGGCTCCCGCCAGGACCTGCTCGACCACAT
Mace              CGCGGCCGGCATCGGGGGCTGCGGCTTCGTCGGCTCCCGCCAGGACCTGCTCGACCACAT
STANLEY           CGCGGCCGGCATCGGGGGCTGCGGCTTCGTCGGCTCCCGCCAGGACCTGCTCGACCACAT
                  ************************************************************
                        v
Drysdale          CTCCGGCCCCGACCACTCGCGCCCCATCATCGTCGTCCGCTACGGCCAGCCGTGGAACCT
RAC875            CTCCGGCCCCGACCACTCGCGCCCCATCATCGTCGTCCGCTACGGCCAGCCGTGGAACCT
Norin61           CTCCGGCCCCGACCACTCGCGCCCCATCATCGTCGTCCGCTACGGCCAGCCGTGGAACCT
Jagger            CTCCGGCCCCGACCACTCGCGCCCCATCATCGTCGTCCGCTACGGCCAGCCGTGGAACCT
Mattis            CTCCGGCCCCGACCACTCGCGCCCCATCATCGTCGTCCGCTACGGCCAGCCGTGGAACCT
Julius            CTCCGGCCCCGACCACTCGCGCCCCATCATCGTCGTCCGCTACGGCCAGCCGTGGAACCT
Arina             CTCCGGCCCCGACCACTCGCGCCCCATCATCGTCGTCCGCTACGGCCAGCCGTGGAACCT
Spelta            CTCCGGCCCCGACCACTCGCGCCCCATCATCGTCGTCCGCTACGGCCAGCCGTGGAACCT
Lancer            CTCCGGGCCCGACCACTCGCGCCCCATCATCGTCGTCCGCTACGGCCAGCCCTGGAACCT
Gladius           CTCCGGGCCCGACCACTCGCGCCCCATCATCGTCGTCCGCTACGGCCAGCCCTGGAACCT
Kukri             CTCCGGGCCCGACCACTCGCGCCCCATCATCGTCGTCCGCTACGGCCAGCCCTGGAACCT
Landmark          CTCCGGGCCCGACCACTCGCGCCCCATCATCGTCGTCCGCTACGGCCAGCCCTGGAACCT
Mace              CTCCGGGCCCGACCACTCGCGCCCCATCATCGTCGTCCGCTACGGCCAGCCCTGGAACCT
STANLEY           CTCCGGGCCCGACCACTCGCGCCCCATCATCGTCGTCCGCTACGGCCAGCCCTGGAACCT
                  ****** ******************************************** ********
                                                         v
Drysdale          CAGCCTGCCGCTCTCGCGCCGCTGGCACATCCTCGTCGGCGAGGAGGACAAGGCGGTGGC
RAC875            CAGCCTGCCGCTCTCGCGCCGCTGGCACATCCTCGTCGGCGAGGAGGACAAGGCGGTGGC
Norin61           CAGCCTGCCGCTCTCGCGCCGCTGGCACATCCTCGTCGGCGAGGAGGACAAGGCGGTGGC
Jagger            CAGCCTGCCGCTCTCGCGCCGCTGGCACATCCTCGTCGGCGAGGAGGACAAGGCGGTGGC
Mattis            CAGCCTGCCGCTCTCGCGCCGCTGGCACATCCTCGTCGGCGAGGAGGACAAGGCGGTGGC
Julius            CAGCCTGCCGCTCTCGCGCCGCTGGCACATCCTCGTCGGCGAGGAGGACAAGGCGGTGGC
Arina             CAGCCTGCCGCTCTCGCGCCGCTGGCACATCCTCGTCGGCGAGGAGGACAAGGCGGTGGC
Spelta            CAGCCTGCCGCTCTCGCGCCGCTGGCACATCCTCGTCGGCGAGGAGGACAAGGCGGTGGC
Lancer            CAGCCTGCCGCTCTCGCGCCGCTGGCACATCCTCGTCGGCGAGGAGGACAAGGCGGTGGC
Gladius           CAGCCTGCCGCTCTCGCGCCGCTGGCACATCCTCGTCGGTGAGGAGGACAAGGCGGTGGC
Kukri             CAGCCTGCCGCTCTCGCGCCGCTGGCACATCCTCGTCGGTGAGGAGGACAAGGCGGTGGC
Landmark          CAGCCTGCCGCTCTCGCGCCGCTGGCACATCCTCGTCGGTGAGGAGGACAAGGCGGTGGC
Mace              CAGCCTGCCGCTCTCGCGCCGCTGGCACATCCTCGTCGGTGAGGAGGACAAGGCGGTGGC
STANLEY           CAGCCTGCCGCTCTCGCGCCGCTGGCACATCCTCGTCGGTGAGGAGGACAAGGCGGTGGC
                  *************************************** ********************
```

```
Drysdale        CGCCGCCGCGGGCGCCGACCGGCACCGCAACCTCTTCCTCGTCTCCCTCGGCGAGCGCGG
RAC875          CGCCGCCGCGGGCGCCGACCGGCACCGCAACCTCTTCCTCGTCTCCCTCGGCGAGCGCGG
Norin61         CGCCGCCGCGGGCGCCGACCGGCACCGCAACCTCTTCCTCGTCTCCCTCGGCGAGCGCGG
Jagger          CGCCGCCGCGGGCGCCGACCGGCACCGCAACCTCTTCCTCGTCTCCCTCGGCGAGCGCGG
Mattis          CGCCGCCGCGGGCGCCGACCGGCACCGCAACCTCTTCCTCGTCTCCCTCGGCGAGCGCGG
Julius          CGCCGCCGCGGGCGCCGACCGGCACCGCAACCTCTTCCTCGTCTCCCTCGGCGAGCGCGG
Arina           CGCCGCCGCGGGCGCCGACCGGCACCGCAACCTCTTCCTCGTCTCCCTCGGCGAGCGCGG
Spelta          CGCCGCCGCGGGCGCCGACCGGCACCGCAACCTCTTCCTCGTCTCCCTCGGCGAGCGCGG
Lancer          CGCCGCCGCGGGCGCCGACCGGCACCGCAACCTCTTCCTCGTCTCCCTCGGCGAGCGCGG
Gladius         CGCCGCCGCGGGCGCCGACCGGCACCGCAACCTCTTCCTCGTCTCCCTCGGCGAGCGCGG
Kukri           CGCCGCCGCGGGCGCCGACCGGCACCGCAACCTCTTCCTCGTCTCCCTCGGCGAGCGCGG
Landmark        CGCCGCCGCGGGCGCCGACCGGCACCGCAACCTCTTCCTCGTCTCCCTCGGCGAGCGCGG
Mace            CGCCGCCGCGGGCGCCGACCGGCACCGCAACCTCTTCCTCGTCTCCCTCGGCGAGCGCGG
STANLEY         CGCCGCCGCGGGCGCCGACCGGCACCGCAACCTCTTCCTCGTCTCCCTCGGCGAGCGCGG
                ************************************************************

Drysdale        CGCCACCACGGCCGTGTCGCTGGTGTGCGTCCGGGCGGACGGCACGGCGCCGGGCGCGCC
RAC875          CGCCACCACGGCCGTGTCGCTGGTGTGCGTCCGGGCGGACGGCACGGCGCCGGGCGCGCC
Norin61         CGCCACCACGGCCGTGTCGCTGGTGTGCGTCCGGGCGGACGGCACGGCGCCGGGCGCGCC
Jagger          CGCCACCACGGCCGTGTCGCTGGTGTGCGTCCGGGCGGACGGCACGGCGCCGGGCGCGCC
Mattis          CGCCACCACGGCCGTGTCGCTGGTGTGCGTCCGGGCGGACGGCACGGCGCCGGGCGCGCC
Julius          CGCCACCACGGCCGTGTCGCTGGTGTGCGTCCGGGCGGACGGCACGGCGCCGGGCGCGCC
Arina           CGCCACCACGGCCGTGTCGCTGGTGTGCGTCCGGGCGGACGGCACGGCGCCGGGCGCGCC
Spelta          CGCCACCACGGCCGTGTCGCTGGTGTGCGTCCGGGCGGACGGCACGGCGCCGGGCGCGCC
Lancer          CGCCACCACGGCCGTGTCGCTGGTGTGCGTCCGGGCGGACGGCACGGCGCCGGGCGCGCC
Gladius         CGCCACCACGGCCGTGTCGCTGGTGTGCGTCCGGGCGGACGGCACGGCGCCGGGCGCGCC
Kukri           CGCCACCACGGCCGTGTCGCTGGTGTGCGTCCGGGCGGACGGCACGGCGCCGGGCGCGCC
Landmark        CGCCACCACGGCCGTGTCGCTGGTGTGCGTCCGGGCGGACGGCACGGCGCCGGGCGCGCC
Mace            CGCCACCACGGCCGTGTCGCTGGTGTGCGTCCGGGCGGACGGCACGGCGCCGGGCGCGCC
STANLEY         CGCCACCACGGCCGTGTCGCTGGTGTGCGTCCGGGCGGACGGCACGGCGCCGGGCGCGCC
                ************************************************************

Drysdale        GCAGTTCGCGTGCAAGCTCGCCGTGGAGAGCGACGGCTGCAGGCTGACCCTGGAGTCGCC
RAC875          GCAGTTCGCGTGCAAGCTCGCCGTGGAGAGCGACGGCTGCAGGCTGACCCTGGAGTCGCC
Norin61         GCAGTTCGCGTGCAAGCTCGCCGTGGAGAGCGACGGCTGCAGGCTGACCCTGGAGTCGCC
Jagger          GCAGTTCGCGTGCAAGCTCGCCGTGGAGAGCGACGGCTGCAGGCTGACCCTGGAGTCGCC
Mattis          GCAGTTCGCGTGCAAGCTCGCCGTGGAGAGCGACGGCTGCAGGCTGACCCTGGAGTCGCC
Julius          GCAGTTCGCGTGCAAGCTCGCCGTGGAGAGCGACGGCTGCAGGCTGACCCTGGAGTCGCC
Arina           GCAGTTCGCGTGCAAGCTCGCCGTGGAGAGCGACGGCTGCAGGCTGACCCTGGAGTCGCC
Spelta          GCAGTTCGCGTGCAAGCTCGCCGTGGAGAGCGACGGCTGCAGGCTGACCCTGGAGTCGCC
Lancer          GCAGTTCGCGTGCAAGCTCGCCGTGGAGAGCGACGGCTGCAGGCTGACCCTGGAGTCGCC
Gladius         GCAGTTCGCGTGCAAGCTCGCCGTGGAGAGCGACGGCTGCAGGCTGACCC-GGAGTCGCC
Kukri           GCAGTTCGCGTGCAAGCTCGCCGTGGAGAGCGACGGCTGCAGGCTGACCCTGGAGTCGCC
Landmark        GCAGTTCGCGTGCAAGCTCGCCGTGGAGAGCGACGGCTGCAGGCTGACCCTGGAGTCGCC
Mace            GCAGTTCGCGTGCAAGCTCGCCGTGGAGAGCGACGGCTGCAGGCTGACCCTGGAGTCGCC
STANLEY         GCAGTTCGCGTGCAAGCTCGCCGTGGAGAGCGACGGCTGCAGGCTGACCCTGGAGTCGCC
                ************************************************** *********

                V                                   v
Drysdale        TCTGGTGTGCAGCAGCTCCCTGTCCGGCGGCCTGCCGGGCGAGGTCAAGTGCCTGCCGGT
RAC875          TCTGGTGTGCAGCAGCTCCCTGTCCGGCGGCCTGCCGGGCGAGGTCAAGTGCCTGCCGGT
Norin61         TCTGGTGTGCAGCAGCTCCCTGTCCGGCGGCCTGCCGGGCGAGGTCAAGTGCCTGCCGGT
Jagger          TCTGGTGTGCAGCAGCTCCCTGTCCGGCGGCCTGCCGGGCGAGGTCAAGTGCCTGCCGGT
Mattis          TCTGGTGTGCAGCAGCTCCCTGTCCGGCGGCCTGCCGGGCGAGGTCAAGTGCCTGCCGGT
Julius          TCTGGTGTGCAGCAGCTCCCTGTCCGGCGGCCTGCCGGGCGAGGTCAAGTGCCTGCCGGT
Arina           TCTGGTGTGCAGCAGCTCCCTGTCCGGCGGCCTGCCGGGCGAGGTCAAGTGCCTGCCGGT
Spelta          TCTGGTGTGCAGCAGCTCCCTGTCCGGCGGCCTGCCGGGCGAGGTCAAGTGCCTGCCGGT
Lancer          GCTGGTGTGCAGCAGCTCCCTGTCCGGCGGCCTGCCCGGCGAGGTCAAGTGCCTGCCGGT
Gladius         GCTGGTGTGCAGCAGCTCCCTGTCCGGCGGCCTGCCCGGCGAGGTCAAGTGCCTGCCGGT
Kukri           GCTGGTGTGCAGCAGCTCCCTGTCCGGCGGCCTGCCCGGCGAGGTCAAGTGCCTGCCGGT
Landmark        GCTGGTGTGCAGCAGCTCCCTGTCCGGCGGCCTGCCCGGCGAGGTCAAGTGCCTGCCGGT
Mace            GCTGGTGTGCAGCAGCTCCCTGTCCGGCGGCCTGCCCGGCGAGGTCAAGTGCCTGCCGGT
STANLEY         GCTGGTGTGCAGCAGCTCCCTGTCCGGCGGCCTGCCCGGCGAGGTCAAGTGCCTGCCGGT
                 *********************************** ***********************
```

```
                            v  v
Drysdale        GCCCAAAGACTTCCTGTCCGGCGACAGTGTGCCCCTCAGCATCCACATCGAGAAGCTCCC
RAC875          GCCCAAAGACTTCCTGTCCGGCGACAGTGTGCCCCTCAGCATCCACATCGAGAAGCTCCC
Norin61         GCCCAAAGACTTCCTGTCCGGCGACAGTGTGCCCCTCAGCATCCACATCGAGAAGCTCCC
Jagger          GCCCAAAGACTTCCTGTCCGGCGACAGTGTGCCCCTCAGCATCCACATCGAGAAGCTCCC
Mattis          GCCCAAAGACTTCCTGTCCGGCGACAGTGTGCCCCTCAGCATCCACATCGAGAAGCTCCC
Julius          GCCCAAAGACTTCCTGTCCGGCGACAGTGTGCCCCTCAGCATCCACATCGAGAAGCTCCC
Arina           GCCCAAAGACTTCCTGTCCGGCGACAGTGTGCCCCTCAGCATCCACATCGAGAAGCTCCC
Spelta          GCCCAAAGACTTCCTGTCCGGCGACAGTGTGCCCCTCAGCATCCACATCGAGAAGCTCCC
Lancer          GCCCAAAGACTTTCTCTCCGGCGACAGTGTGCCCCTCAGCATCCACATCGAGAAGCTCCC
Gladius         GCCCAAAGACTTTCTCTCCGGCGACAGTGTGCCCCTCAGCATCCACATCGAGAAGCTCCC
Kukri           GCCCAAAGACTTTCTCTCCGGCGACAGTGTGCCCCTCAGCATCCACATCGAGAAGCTCCC
Landmark        GCCCAAAGACTTTCTCTCCGGCGACAGTGTGCCCCTCAGCATCCACATCGAGAAGCTCCC
Mace            GCCCAAAGACTTTCTCTCCGGCGACAGTGTGCCCCTCAGCATCCACATCGAGAAGCTCCC
STANLEY         GCCCAAAGACTTTCTCTCCGGCGACAGTGTGCCCCTCAGCATCCACATCGAGAAGCTCCC
                ************ ** ********************************************
                  v v    v                                      v
Drysdale        GGCTCCCCCAGCTCCTCCTCTAGGACCAGGAGTGGCGCCGGCCTGTACTTCCCCGGTCGC
RAC875          GGCTCCCCCAGCTCCTCCTCTAGGACCAGGAGTGGCGCCGGCCTGTACTTCCCCGGTCGC
Norin61         GGCTCCCCCAGCTCCTCCTCTAGGACCAGGAGTGGCGCCGGCCTGTACTTCCCCGGTCGC
Jagger          GGCTCCCCCAGCTCCTCCTCTAGGACCAGGAGTGGCGCCGGCCTGTACTTCCCCGGTCGC
Mattis          GGCTCCCCCAGCTCCTCCTCTAGGACCAGGAGTGGCGCCGGCCTGTACTTCCCCGGTCGC
Julius          GGCTCCCCCAGCTCCTCCTCTAGGACCAGGAGTGGCGCCGGCCTGTACTTCCCCGGTCGC
Arina           GGCTCCCCCAGCTCCTCCTCTAGGACCAGGAGTGGCGCCGGCCTGTACTTCCCCGGTCGC
Spelta          GGCTCCCCCAGCTCCTCCTCTAGGACCAGGAGTGGCGCCGGCCTGTACTTCCCCGGTCGC
Lancer          GGTTGCCCCCGCTCCTCCTCTAGGACCAGGAGTGGCGCCGGCCTGTACCTCCCCGGTCGC
Gladius         GGTTGCCCCCGCTCCTCCTCTAGGACCAGGAGTGGCGCCGGCCTGTACCTCCCCGGTCGC
Kukri           GGTTGCCCCCGCTCCTCCTCTAGGACCAGGAGTGGCGCCGGCCTGTACCTCCCCGGTCGC
Landmark        GGTTGCCCCCGCTCCTCCTCTAGGACCAGGAGTGGCGCCGGCCTGTACCTCCCCGGTCGC
Mace            GGTTGCCCCCGCTCCTCCTCTAGGACCAGGAGTGGCGCCGGCCTGTACCTCCCCGGTCGC
STANLEY         GGTTGCCCCCGCTCCTCCTCTAGGACCAGGAGTGGCGCCGGCCTGTACCTCCCCGGTCGC
                ** * **** ************************************** ***********
                      V        v        v                 v
Drysdale        CGCCACCCCTCCTCCCTGCCCGGCCGCCACCATCCCGCCTTCTCGTCCGTCCTCCGCCGG
RAC875          CGCCACCCCTCCTCCCTGCCCGGCCGCCACCATCCCGCCTTCTCGTCCGTCCTCCGCCGG
Norin61         CGCCACCCCTCCTCCCTGCCCGGCCGCCACCATCCCGCCTTCTCGTCCGTCCTCCGCCGG
Jagger          CGCCACCCCTCCTCCCTGCCCGGCCGCCACCATCCCGCCTTCTCGTCCGTCCTCCGCCGG
Mattis          CGCCACCCCTCCTCCCTGCCCGGCCGCCACCATCCCGCCTTCTCGTCCGTCCTCCGCCGG
Julius          CGCCACCCCTCCTCCCTGCCCGGCCGCCACCATCCCGCCTTCTCGTCCGTCCTCCGCCGG
Arina           CGCCACCCCTCCTCCCTGCCCGGCCGCCACCATCCCGCCTTCTCGTCCGTCCTCCGCCGG
Spelta          CGCCACCCCTCCTCCCTGCCCGGCCGCCACCATCCCGCCTTCTCGTCCGTCCTCCGCCGG
Lancer          CGCCACGCCTCCTCCTTGCCCGGCGGCCACCATCCCGCCTTCCCGTCCGTCCTCCGCCGG
Gladius         CGCCACGCCTCCTCCTTGCCCGGCGGCCACCATCCCGCCTTCCCGTCCGTCCTCCGCCGG
Kukri           CGCCACGCCTCCTCCTTGCCCGGCGGCCACCATCCCGCCTTCCCGTCCGTCCTCCGCCGG
Landmark        CGCCACGCCTCCTCCTTGCCCGGCGGCCACCATCCCGCCTTCCCGTCCGTCCTCCGCCGG
Mace            CGCCACGCCTCCTCCTTGCCCGGCGGCCACCATCCCGCCTTCCCGTCCGTCCTCCGCCGG
STANLEY         CGCCACGCCTCCTCCTTGCCCGGCGGCCACCATCCCGCCTTCCCGTCCGTCCTCCGCCGG
                ****** ******** ******** ***************** *****************
                                     v
Drysdale        CTCCTCCGACA-------------------------------------------------
RAC875          CTCCTCCGAC--------------------------------------------------
Norin61         CTCCTCCGACAACGTCGCCGTTAAGACGGTGATCACCGATCAGAGCTACAAGAAACGGAA
Jagger          CTCCTCCGACAACGTCGCCGTTAAGACGGTGATCACCGATCAGAGCTACAAGAAACGGAA
Mattis          CTCCTCCGACAACGTCGCCGTTAAGACGGTGATCACCGATCAGAGCTACAAGAAACGGAA
Julius          CTCCTCCGACAACGTCGCCGTTAAGACGGTGATCACCGATCAGAGCTACAAGAAACGGAA
Arina           CTCCTCCGACAACGTCGCCGTTAAGACGGTGATCACCGATCAGAGCTACAAGAAACGGAA
Spelta          CTCCTCCGACAACGTCGCCGTTAAGACGGTGATCACCGATCAGAGCTACAAGAAACGGAA
Lancer          CTCCTCCGACAACGTCGCAGTCAAGACGGTGATCACCGATCAGAGCTACAAGAAACGGAA
Gladius         CT----------------------------------------------------------
Kukri           CTCCTCCGACAA------------------------------------------------
Landmark        CTCCTCCGACAACGTCGCAGTCAAGACGGTGATCACCGATCAGAGCTACAAGAAACGGAA
Mace            CTCCTCCGACAACGTCGCAGTCAAGACGGTGATCACCGATCAGAGCTACAAGAAACGGAA
STANLEY         CTCCTCCGACAACGTCGCAGTCAAGACGGTGATCACCGATCAGAGCTACAAGAAACGGAA
                **
```

```
                                          Stop
Drysdale          ------------------------------------------------------------
RAC875            ------------------------------------------------------------
Norin61           GTCCGCCAACCCAAGGAAGCTGTAGGGAGGACGCCGATGGGCTGGAGTAGC-ACGATGAT
Jagger            GTCCGCCAACCCAAGGAAGCTGTAGGGAGGACGCCGATGGGCTGGAGTAGC-ACGATGAT
Mattis            GTCCGCCAACCCAAGGAAGCTGTAGGGAGGACGCCGATGGGCTGGAGTAGC-ACGATGAT
Julius            GTCCGCCAACCCAAGGAAGCTGTAGGGAGGACGCCGATGGGCTGGAGTAGC-ACGATGAT
Arina             GTCCGCCAACCCAAGGAAGCTGTAGGGAGGACGCCGATGGGCTGGAGTAGC-ACGATGAT
Spelta            GTCCGCCAACCCAAGGAAGCTGTAGGGAGGACGCCGATGGGCTGGAGTAGC-ACGATGAT
Lancer            GTCTGCCAACCCAAGGAAGCTGTAGGGAGGACGCTGATGGTCTGGGTCGACTACGATGAT
Gladius           ------------------------------------------------------------
Kukri             ------------------------------------------------------------
Landmark          GTCTGCCAACCCAAGGAAGCTGTAGGGAGGACGCTGATGGTCTGGGTCGACTACGATGAT
Mace              GTCTGCCAACCCAAGGAAGCTGTAGGGAGGACGCTGATGGTCTGGGTCGACTACGATGAT
STANLEY           GTCTGCCAACCCAAGGAAGCTGTAGGGAGGACGCTGATGGTCTGGGTCGACTACGATGAT


Drysdale          ------------------------------------------------------------
RAC875            ------------------------------------------------------------
Norin61           GAATGGTGGACTAGTAGTATGCTTAGCAATAAGCTACGTAGTATTTTGTAATGTGCAATC
Jagger            GAATGGTGGACTAGTAGTATGCTTAGCAATAAGCTACGTAGTATTTTGTAATGTGCAATC
Mattis            GAATGGTGGACTAGTAGTATGCTTAGCAATAAGCTACGTAGTATTTTGTAATGTGCAATC
Julius            GAATGGTGGACTAGTAGTATGCTTAGCAATAAGCTACGTAGTATTTTGTAATGTGCAATC
Arina             GAATGGTGGACTAGTAGTATGCTTAGCAATAAGCTACGTAGTATTTTGTAATGTGCAATC
Spelta            GAATGGTGGACTAGTAGTATGCTTAGCAATAAGCTACGTAGTATTTTGTAATGTGCAATC
Lancer            GAACGGCCGACTAGTAGTATGCTTAGCAATAAGCTACGTAGTATTTTGTAATGTGCAATC
Gladius           ------------------------------------------------------------
Kukri             ------------------------------------------------------------
Landmark          GAACGGCCGACTAGTAGTATGCTTAGCAATAAGCTACGTAGTATTTTGTAATGTGCAATC
Mace              GAACGGCCGACTAGTAGTATGCTTAGCAATAAGCTACGTAGTATTTTGTAATGTGCAATC
STANLEY           GAACGGCCGACTAGTAGTATGCTTAGCAATAAGCTACGTAGTATTTTGTAATGTGCAATC


Drysdale          ------------------------------------------------------------
RAC875            ------------------------------------------------------------
Norin61           AGTGTTCTGTTTCTTTGTTAATCTGATGATGAAGTCCATGGTTTTCAGTCTGAAATAACC
Jagger            AGTGTTCTGTTTCTTTGTTAATCTGATGATGAAGTCCATGGTTTTCAGTCTGAAATAACC
Mattis            AGTGTTCTGTTTCTTTGTTAATCTGATGATGAAGTCCATGGTTTTCAGTCTGAAATAACC
Julius            AGTGTTCTGTTTCTTTGTTAATCTGATGATGAAGTCCATGGTTTTCAGTCTGAAATAACC
Arina             AGTGTTCTGTTTCTTTGTTAATCTGATGATGAAGTCCATGGTTTTCAGTCTGAAATAACC
Spelta            AGTGTTCTGTTTCTTTGTTAATCTGATGATGAAGTCCATGGTTTTCAGTCTGAAATAACC
Lancer            AGCGTTCTGTTTCTTTGTTAATCTGATGATGAAGTCCATGGTTTTCAGTCTGAAATAACC
Gladius           ------------------------------------------------------------
Kukri             ------------------------------------------------------------
Landmark          AGCGTTCTGTTTCTTTGTTAATCTGATGATGAAGTCCATGGTTTTCAGTCTGAAATAACC
Mace              AGCGTTCTGTTTCTTTGTTAATCTGATGATGAAGTCCATGGTTTTCAGTCTGAAATAACC
STANLEY           AGCGTTCTGTTTCTTTGTTAATCTGATGATGAAGTCCATGGTTTTCAGTCTGAAATAACC


Drysdale          ------------------------------------------------------------
RAC875            ------------------------------------------------------------
Norin61           AGACACCGTGCATTCCTTTTGTTCAAGCCTGCCTGCATATTTTGTTGTAGTTACTGATGA
Jagger            AGACACCGTGCATTCCTTTTGTTCAAGCCTGCCTGCATATTTTGTTGTAGTTACTGATGA
Mattis            AGACACCGTGCATTCCTTTTGTTCAAGCCTGCCTGCATATTTTGTTGTAGTTACTGATGA
Julius            AGACACCGTGCATTCCTTTTGTTCAAGCCTGCCTGCATATTTTGTTGTAGTTACTGATGA
Arina             AGACACCGTGCATTCCTTTTGTTCAAGCCTGCCTGCATATTTTGTTGTAGTTACTGATGA
Spelta            AGACACCGTGCATTCCTTTTGTTCAAGCCTGCCTGCATATTTTGTTGTAGTTACTGATGA
Lancer            AGACACCGTGCATTCC-----------------------TTTTGTTGTAGTTAC---TGA
Gladius           ------------------------------------------------------------
Kukri             ------------------------------------------------------------
Landmark          AGACACCGTGCATTCC-----------------------TTTTGTTGTAGTTAC---TGA
Mace              AGACACCGTGCATTCC-----------------------TTTTGTTGTAGTTAC---TGA
STANLEY           AGACACCGTGCATTCC-----------------------TTTTGTTGTAGTTAC---TGA
```
